# Global, regional, and national burden of meningitis among children, 1990–2021: An analysis of the global burden of disease study 2021

**DOI:** 10.1371/journal.pone.0326992

**Published:** 2025-06-24

**Authors:** Jun Li, Xiang Li, Shunhong Luo, Haishen Zhou

**Affiliations:** 1 The Second Xiangya Hospital, Central South University, Changsha, Hunan, P. R. China; 2 Department of Obstetrics and Gynecology, The Second Xiangya Hospital, Central South University, Changsha, Hunan, P. R. China; 3 Department of Orthopedics, Hunan University of Medicine General Hospital, Huaihua, Hunan, P. R. China; 4 The People’s Hospital of Baoan Shenzhen, Shenzhen, P. R. China; Shiraz University of Medical Sciences, ISLAMIC REPUBLIC OF IRAN

## Abstract

**Background:**

Childhood meningitis remains a serious and life-threatening infectious disease with considerable risk of long-term sequelae. This study aimed to provide a comprehensive evaluation of the global burden and temporal trends of childhood meningitis from 1990 to 2021.

**Methods:**

We extracted data from the Global Burden of Disease (GBD) Study 2021 to estimate incident cases, incidence rates, deaths, and mortality rates of childhood meningitis at global, regional, and national levels. We further assessed the etiological composition of childhood meningitis across different age groups and sociodemographic index (SDI) regions. Additionally, cross-country inequality analyses were conducted to measure the distributional inequality of childhood meningitis burden across countries.

**Results:**

In 2021, childhood meningitis accounted for approximately 1.33 million incident cases and 112,000 deaths worldwide. The global incidence rate of childhood meningitis decreased by 59.8%, from 164.80 per 100,000 in 1990 to 66.24 per 100,000 in 2021. The global mortality rate also decreased by 69.18%, from 18.12 per 100,000 in 1990 to 5.59 per 100,000 in 2021. In 2021, the top three etiologies responsible for global childhood meningitis deaths were Streptococcus pneumoniae (17.01%), Neisseria meningitidis (14.03%), and Klebsiella pneumoniae (12.11%). Notably, the leading etiologies of neonatal meningitis deaths were Group B Streptococcus (22.70%), Klebsiella pneumoniae (16.97%), and viral etiologies (15.35%). Cross-country inequality analysis revealed that low SDI regions bore a disproportionate burden.

**Conclusions:**

Despite significant progress over the past three decades, childhood meningitis remains a major public health challenge, particularly in resource-limited settings. Concerted efforts are required to reduce the unacceptable health inequities associated with this devastating disease.

## Introduction

Meningitis, an inflammation of the protective membranes covering the brain and spinal cord, remains a major global health concern, particularly among children [1,2]. Despite significant advances in medical science and public health initiatives, childhood meningitis continues to impose an alarming burden. Additionally, there are substantial regional and national disparities in incidence and mortality rates [3–5]. According to the Global Burden of Disease (GBD) study, approximately 1.28 million meningitis cases were reported globally in 2019, with over 60% of cases occurring in children [6]. In 2021, the World Health Organization (WHO) launched its first global roadmap, outlining a vision and strategy to eliminate meningitis epidemics by 2030 [7]. To achieve this ambitious goal, targeted interventions to mitigate the global disease burden of childhood meningitis must be prioritized, which demands a systematic evaluation of its impact on child health worldwide.

The etiology of childhood meningitis is multifactorial, involving various pathogens such as bacteria, viruses, and fungi [8–10]. If not diagnosed and treated promptly, affected children face a considerable risk of long-term sequelae such as hearing loss, cognitive impairment, developmental delay, and motor deficits [11,12]. Therefore, understanding the distribution of these pathogens and their associated burden is crucial for developing targeted interventions and improving health outcomes [13]. Geographical disparities in meningitis incidence and mortality reflect differences in socioeconomic status, healthcare accessibility, and vaccination coverage [14]. Regions with limited healthcare infrastructure and lower immunization rates remain particularly vulnerable to meningitis outbreaks, highlighting the need for focused public health strategies. Additionally, examining the age distribution of meningitis cases reveals critical demographic patterns that can guide prevention and treatment efforts. To date, however, no study has comprehensively evaluated the global burden of childhood meningitis.

This study aims to fill this gap by providing a comprehensive overview of the trends in childhood meningitis incidence and mortality from 1990 to 2021, utilizing data from the GBD 2021 database. By analyzing these trends at the global, regional, and national levels, we seek to elucidate the burden of childhood meningitis and its contributing etiologies. Furthermore, we explore the distribution of disease burden by age and sociodemographic index (SDI). This research will deepen the understanding of the challenges posed by childhood meningitis and inform public health policies to effectively reduce its impact on vulnerable populations.

## Methods

### Overview and data sources

Data on the burden of childhood meningitis, including incidence and mortality, were obtained from the Global Burden of Disease (GBD) 2021 database (https://vizhub.healthdata.org/gbd-results/). The GBD 2021 provides a comprehensive epidemiological assessment for 371 diseases and injuries, along with 88 risk factors, covering 204 countries or territories from 1990 to 2021 [15,16]. GBD 2021 classifies all risk factors into a hierarchical system consisting of four levels, where level 1 represents the broadest risk categories (including environmental and occupational, behavioral, and metabolic risks), and level 4 denotes the most granular level within the hierarchy [16]. GBD 2021 is a high-quality epidemiological database that has been extensively utilized for describing disease burden, forecasting trends, and analyzing health inequalities. In this study, children (0–14 years) were categorized into three distinct age groups: 0–4 years, 5–9 years, and 10–14 years. Considering the notably high incidence and mortality rates of neonatal meningitis, a separate analysis was conducted for neonates (0–27 days), which were further subdivided into early neonatal stage (0–6 days) and late neonatal stage (7–27 days).

The GBD 2021 also estimates the socio-demographic index (SDI) for different countries and regions in different years, classifying them into five levels based on the SDI quintile. The SDI with its quintile can be accessed from the Institute for Health Metrics and Evaluation (https://ghdx.healthdata.org/search/site/SDI). The SDI is a comprehensive index reflecting developmental status, incorporating three dimensions: total fertility rate among females under 25 years, mean education levels for individuals over 15 years, and per capita income [15]. SDI values range from 0 to 1, with higher values indicating better socioeconomic conditions. The Ethics Review Committee of Xiangya Second Hospital, Central South University, determined that ethical approval was waived for this study, as it utilized publicly available data with no identifiable personal information.

### Descriptive analysis

A descriptive analysis was conducted to comprehensively assess the incident cases, incidence rate, deaths, and mortality rate for childhood meningitis at the global, regional, and national levels from 1990 to 2021. This study also investigated the etiological composition of childhood meningitis burden across different age groups and SDI regions. Additionally, associations between the SDI and incidence and mortality rates were analyzed.

### Trend analysis

To quantify temporal trends in the burden of childhood meningitis, the estimated annual percent change (EAPC) was calculated. Both the EAPC and its 95% confidence interval (CI) were derived using a linear regression model to estimate the annual percentage change over specific periods [17]. Briefly, the model assumes a linear relationship between the logarithm of the rate and time, expressed as: y = α + βx + ε, where y refers to ln(Rate), x is the calendar year, and β is the regression coefficient. Then the EAPC can be calculated as: EAPC = 100 * [exp(β)-1]. An EAPC and the lower boundary of 95% CI greater than zero indicate an increasing trend, whereas values below zero signify a decreasing trend.

### Cross-country inequality analysis

The slope inequality index (SII) and concentration index were employed to measure absolute and relative distributional inequality in the childhood meningitis burden across countries. The SII is an index that reflects the absolute inequalities in health indicators between the most dominant and least dominant subgroups within a population. The SII is calculated by regressing national death rates on an SDI-associated relative position scale [18]. The concentration index is derived from the numerical integration of the area under the Lorenz curve which was fitted using the cumulative proportions of deaths and the cumulative population distribution ranked by SDI [19]. A negative SII or concentration index indicates that higher SDI values are associated with lower death rates, and vice versa. The absolute value of the SII or concentration index reflects the degree of inequality, with larger values signifying greater inequality levels.

All statistical analyses and plotting were conducted using R Studio (version 2023.06.2 + 561) and R software (version R-4.3.3). A P-value of less than 0.05 was considered statistically significant.

## Results

### Incidence of childhood meningitis at global, regional, and national levels

**Fig 1:**
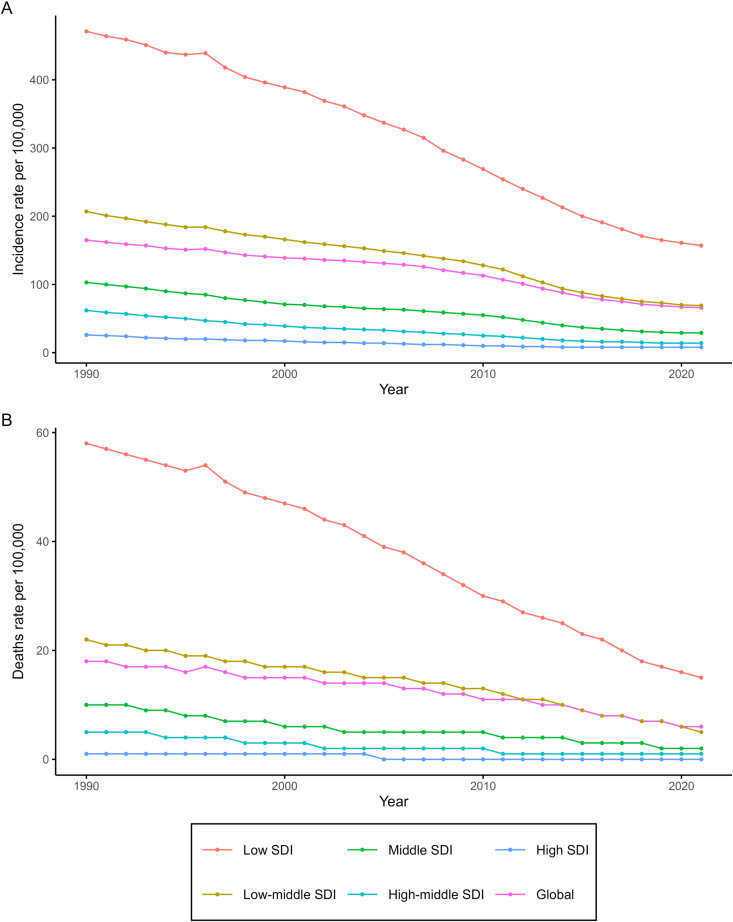
Trends in childhood meningitis incidence rates(A) and death rates(B) by SDI regions, 1990-2021.

Globally, in 2021, the incident cases of childhood meningitis were estimated at 1.33 million (95% UI: 1.11 to 1.58 million), representing a 53.5% decrease (95% UI: −55.65% to −51.08%) from 2.87 million (95% UI: 2.30 to 3.49 million) in 1990. The global incidence rate also declined by 59.8% (95% UI: −61.66% to −57.71%), from 164.80 (95% UI: 132.10 to 200.46) per 100,000 in 1990 to 66.24 (95% UI: 55.03 to 78.41) per 100,000 in 2021, with an EAPC of −2.96% (95% CI: −3.27% to −2.65%) (Fig 1 and S1 Table in S1 File).

At the regional level, Western Sub-Saharan Africa (220.05 [95% UI: 184.27 to 260.17] per 100,000), Eastern Sub-Saharan Africa (132.44 [95% UI: 111.83 to 155.42] per 100,000), and Central Sub-Saharan Africa (119.72 [95% UI: 101.50 to 140.02] per 100,000) had the highest incidence rates of childhood meningitis (S1 Table in S1 File). From 1990 to 2021, incidence rates showed declined trends in all 21 GBD regions, with the largest decreases observed in East Asia (EAPC: −6.75% [95% CI: −6.96% to −6.54%]), High-income North America (EAPC: −6.27% [95% CI: −6.95% to −5.59%]), and Andean Latin America (EAPC: −5.05% [95% CI: −5.21% to −4.80%]) (S1 Table in S1 File).

**Fig 2:**
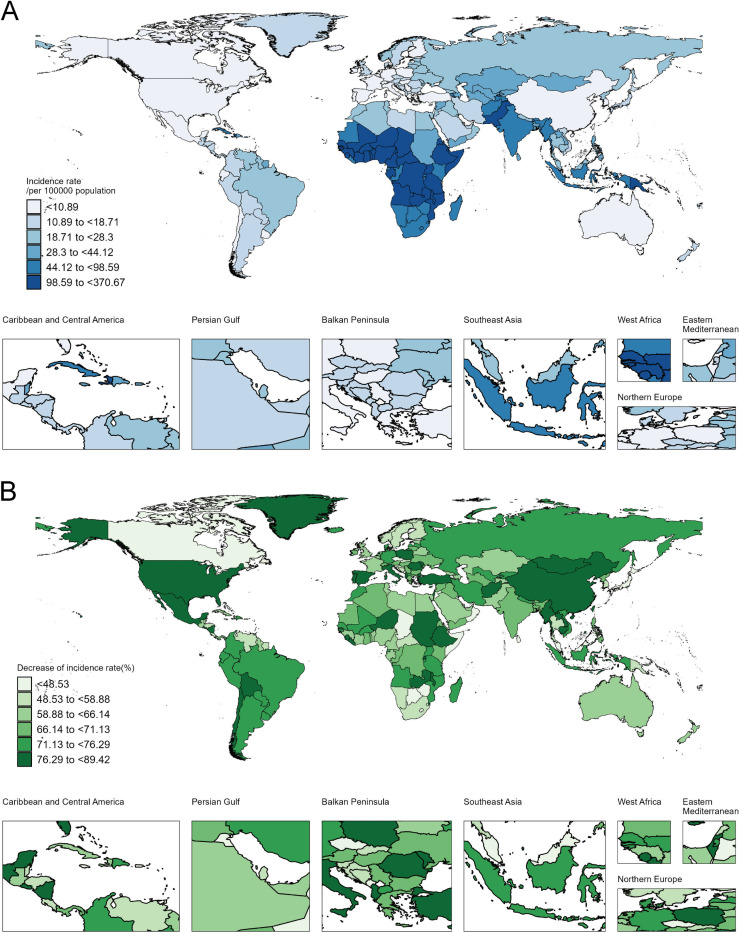
The incidence attributable to childhood meningitis in 204 countries and territories. (A), The incidence rate of childhood meningitis in 2021. (B), The changes in incidence rate from 1990 to 2021.

There were substantial cross-country variations in the incidence of childhood meningitis (Fig 2). In 2021, the highest incidence rates were reported in South Sudan (370.67 per 100,000), Chad (304.39 per 100,000), and Guinea (280.70 per 100,000) (S2 Table in S1 File). From 1990 to 2021, the fastest declines in incidence rates were observed in Turkey (EAPC: −7.35% [95% CI: −7.77% to −6.93%]), Poland (EAPC: −7.2% [95% CI: −7.54% to −6.86%]), and China (EAPC: −6.91% [95% CI: −7.14% to −6.68%]).

### Fatal burden of childhood meningitis at global, regional, and national levels

In 2021, the global estimated deaths attributed to childhood meningitis were 112,373 (95% UI: 80,908–154,126), representing a 64.35% (95% UI: −72.68% to −52.42%) decrease from 315,176 (95% UI: 270,536–372,951) in 1990. The global mortality rate of childhood meningitis also decreased by 69.18% (95% UI: −76.38% to −58.87%), from 18.12 (95% UI: 15.56 to 21.4) per 100,000 in 1990 to 5.59 (95% UI: 4.02 to 7.66) per 100,000 in 2021, with an EAPC of −3.4% (95% CI: −3.76% to −3.04%) (Fig 1 and S3 Table in S1 File).

Regionally, Western Sub-Saharan Africa (21.99 [95% UI:13.35 to 34.59] per 100,000), Eastern Sub-Saharan Africa (11.66 [95% UI: 8.27 to 16.27] per 100,000), and Central Sub-Saharan Africa (9.74 [95% UI: 6.61 to 16.99] per 100,000) had the highest mortality rates. From 1990 to 2021, mortality rates declined in all 21 GBD regions (S3 Table in S1 File). The most significant decreases in mortality rates were observed in East Asia (EAPC: −8.26% [95% CI: −8.69% to −7.83%]), Tropical Latin America (EAPC: −8.19% [95% CI: −8.59% to −7.8%]), and Andean Latin America (EAPC: −7.12% [95% CI: −7.33% to −6.92%]).

Cross-country variability in childhood meningitis mortality remained substantial (S1 Fig in S1 File). In 2021, the countries with the highest mortality rates were South Sudan (36.56 [95% UI: 25.1 to 50.32] per 100,000), Chad (30.98 [95% UI: 20.58 to 44.7] per 100,000), and Niger (28.14 [95% UI: 17.75 to 47.13] per 100,000) (S4 Table in S1 File). From 1990 to 2021, the fastest declines in mortality rates were observed in Saudi Arabia (EAPC: −10.02% [95% CI: −10.37% to −9.68%]), Kyrgyzstan (EAPC: −9.48% [95% CI: −10.04% to −8.92%]), and Turkey (EAPC: −9.39% [95% CI: −9.85% to −8.92%]).

### Global burden of impairments attributed to childhood meningitis

The GBD 2021 study evaluated the burden of impairments attributable to childhood meningitis (see S5 Table in S1 File). In 2021, developmental intellectual disability was the most prevalent impairment linked to childhood meningitis, with 552,778 prevalent cases reported. This total comprises 287,727 cases of borderline intellectual disability, 195,258 cases of mild intellectual disability, 46,878 cases of moderate intellectual disability, and 22,914 cases of severe intellectual disability.

In 2021, the prevalence rates for four major impairments attributed to childhood meningitis were as follows: developmental intellectual disability (27.48 [95% UI: 19.92 to 37.68] per 100,000), hearing loss (12.41 [95% UI: 9.47 to 16.06] per 100,000), epilepsy (5.54 [95% UI: 4.63 to 6.53] per 100,000), and blindness and vision loss (1.97 [95% UI: 1.45 to 2.55] per 100,000). Notably, from 1990 to 2021, prevalence rates of these four impairments have shown a decline, with epilepsy experiencing the most rapid decrease (EAPC: −3.69% [95% CI: −4.02% to −3.36%]).

### Etiological composition of childhood meningitis at the global and regional levels

In 2021, the top three etiologies responsible for global childhood meningitis deaths were Streptococcus pneumoniae (17.01% [95% UI: 15.78% to 18.21%]), Neisseria meningitidis (14.03% [95% UI: 12.97% to 15.03%]), and Klebsiella pneumoniae (12.11% [95% UI: 10% to 14.56%]) (S6 Table in S1 File). In 1990, Neisseria meningitidis was the predominant cause, accounting for 19.95% (95% UI: 18.72% to 21.17%) of childhood meningitis deaths (S7 Table in S1 File). From 1990 to 2021, mortality rates declined most substantially for childhood meningitis caused by Haemophilus influenzae (S8 Table in S1 File).

The etiological composition of childhood meningitis deaths varied considerably across GBD regions in 2021 (S2 Fig in S1 File). High-SDI regions reported the highest proportion of deaths attributed to Klebsiella pneumoniae (15.49%), whereas in the other four SDI quintiles, Streptococcus pneumoniae was the leading cause (S6 Table in S1 File). At the GBD regional level, Western Sub-Saharan Africa had the highest mortality rates for each etiology among the 21 GBD regions (S2 Fig in S1 File). Furthermore, Streptococcus pneumoniae was the leading cause of childhood meningitis death in 11 GBD regions, Klebsiella pneumoniae in 8 GBD regions, and Neisseria meningitidis in 2 GBD regions (S6 Table in S1 File).

### Burden and etiological composition of childhood meningitis by age group

In 2021, 1.02 (95% UI: 0.84 to 1.22) million incident cases of meningitis occurred globally in children younger than 5 years, accounting for 76.20% of the total childhood meningitis cases (S9 Table in S1 File). Notably, incidence rates of meningitis decreased with age: 1185.02 (95% UI: 965.44 to 1,469.36) per 100,000 in neonates, 154.31(95% UI: 127.44 to 185.38) per 100,000 in children younger than 5 years, and 18.93 (95% UI: 12.42 to 27.17) per 100,000 in children aged 10–14 years.

**Fig 3:**
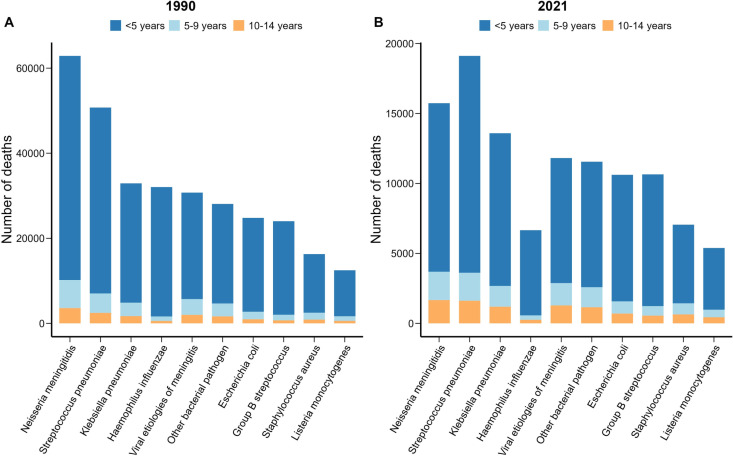
Global number of meningitis deaths by etiology and age group in 1990(A) and 2021(B).

Children younger than 5 years accounted for 81.1% of childhood meningitis deaths in 2021(S10 Table in S1 File). Among children younger than 5 years, the top three etiologies responsible for meningitis deaths were Streptococcus pneumoniae (17.01%), Neisseria meningitidis (13.23%), and Klebsiella pneumoniae (12.11%) (S11 Table in S1 File). Notably, the leading etiologies of neonatal meningitis deaths were Group B Streptococcus (22.70%), Klebsiella pneumoniae (16.97%), and viral etiologies (15.35%) (Fig 3 and S11 Table in S1 File). For children 5–9 and 10–14 years, the top three etiologies were Neisseria meningitidis, Streptococcus pneumoniae, and viral etiologies (Fig 3 and S11 Table in S1 File).

### Neonatal meningitis and attributable risk factors

In 2021, there were an estimated 115,483 (95%UI: 94,084–143,192) incident cases of neonatal meningitis globally, with 50,529 (95% UI: 40,973–62,597) occurring in early neonates and 64,953 (95% UI: 53,089–80,529) in late neonates. The global incidence rate of neonatal meningitis decreased from 2,287.34 (95% UI: 1,766.1 to 2,920.33) per 100,000 in 1990–1,185.02 (95% UI: 965.44 to 1,469.36) per 100,000 in 2021, with an EAPC of −2.39% (95% CI: −2.68% to −2.10%). Regionally, Western Sub-Saharan Africa had the highest neonatal meningitis incidence rate in 2021, at 3,609.58 (95% UI: 2,924.35 to 4,442.7) per 100,000 (S12 Table in S1 File). Incidence rates declined across all 21 GBD regions between 1990 and 2021, with East Asia exhibiting the largest decrease (EAPC: −7.11% [95% CI: −7.33% to −6.9%]) (S12 Table in S1 File). Considerable heterogeneity in neonatal meningitis incidence was observed across countries in 2021, ranging from 24.03 (95% UI: 18.2 to 31.75) per 100,000 in Andorra to 6,786.3 (95% UI: 5,449.79 to 8,543.01) per 100,000 in South Sudan (S13 Table in S1 File).

In 2021, there were an estimated 12,639 (95%UI: 9,819–16,643) deaths due to neonatal meningitis worldwide, including 6,849 (95% UI: 5,261–9,318) among early neonates (0−6 days) and 5,790 (95% UI: 4,475–7,771) among late neonates (7−27 days). The global neonatal meningitis mortality rate decreased from 303.44 (95% UI: 262.15 to 390.81) per 100,000 in 1990 to 129.69 (95% UI: 100.76 to 170.78) per 100,000 in 2021, with an EAPC of −2.8% (95% CI: −3.16% to −2.44%). At the regional level, Western Sub-Saharan Africa had the highest mortality rate in 2021, at 391.58 (296.95 to 530.94) per 100,000 (S14 Table in S1 File). Mortality rates declined across all 21 GBD regions from 1990 to 2021 (S14 Table in S1 File), with East Asia demonstrating the largest decline (EAPC: −6.91% [95% CI: −7.34% to −6.48%]). In 2021, notable disparities in neonatal meningitis mortality were observed at the country level, ranging from 0.12 (95% UI: 0.06 to 0.20) per 100,000 in Andorra to 717.41 (95% UI: 439.05 to 1,129.55) per 100,000 in South Sudan (S13 Table in S1 File).

**Fig 4:**
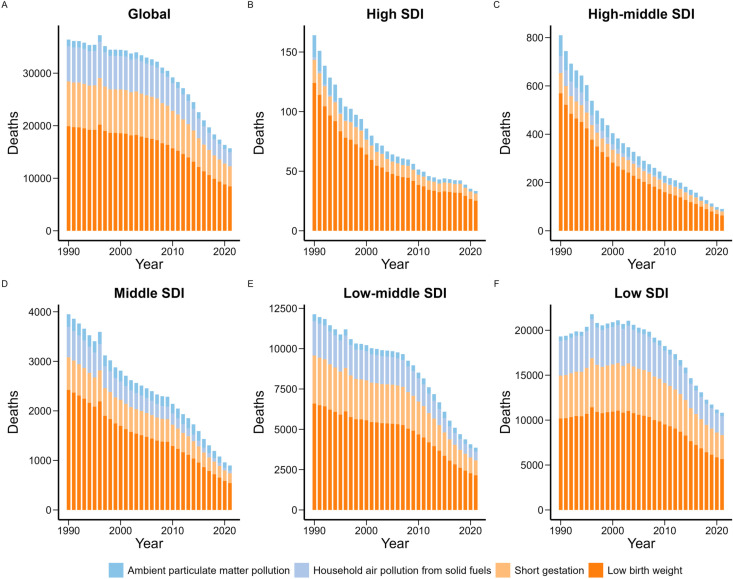
Level 4 risk factors contributing to neonatal meningitis-related death globally and across different regions, 1990-2021. (A). Global, (B). High SDI, (C). high-middle SDI, (D). Middle SDI, (E). Low-middle SDI, (F). Low SDI.

The GBD 2021 only analyzed the risk factors of neonatal meningitis (S15 Table in S1 File). In 2021, four level 4 risk factors were identified for neonatal meningitis mortality, including low birth weight, short gestation, ambient particulate matter pollution, and household air pollution from solid fuels. Among these, low birth weight was the most significant contributor globally and across regions with different SDI levels (Fig 4). Furthermore, the contribution of low birth weight to neonatal meningitis mortality increased with increasing SDI levels (S3 Fig in S1 File).

### Relationship between the burden of childhood meningitis and SDI

In 2021, low-SDI and low-middle-SDI regions accounted for 84.38% of the global childhood meningitis cases and 89.13% of the deaths. In 2021, The incidence rate of childhood meningitis was 157.43 (95% UI: 132.02 to 184.47) per 100,000 in low SDI regions, markedly higher than the rate of 8.02 (95% UI: 5.92 to 10.85) per 100,000 observed in high SDI regions (S1 Table in S1 File). Similarly, the mortality rate in low SDI regions was 14.91 (95% UI: 10.09 to 22.26) per 100,000–70 times greater than the rate in high SDI regions (0.21 [95% UI: 0.19 to 0.22] per 100,000). Further analysis confirmed that both incidence and mortality rates were significantly lower in high-SDI countries compared to low-SDI countries (S4 Fig in S1 File). Spearman rank correlation analysis revealed a strong negative correlation between SDI and both incidence rates (R = −0.7387, P < 0.0001), as well as mortality rates (R = −0.829, P < 0.0001).

### Health inequality analysis

**Fig 5:**
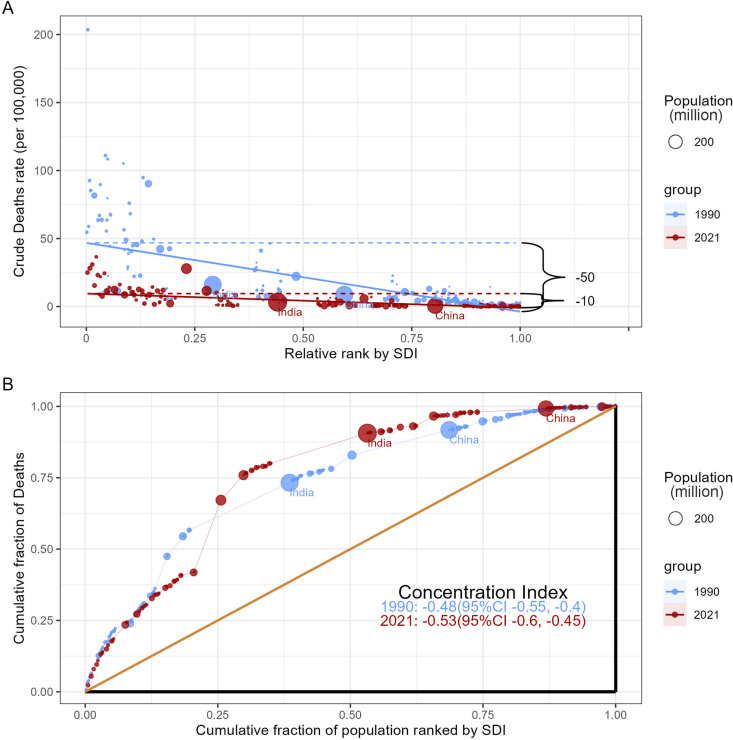
Health inequality regression curves(A) and concentration curves (B) for the death of childhood meningitis worldwide, 1990 and 2021.

To further elucidate the relationship between childhood meningitis burden and SDI, we conducted a health inequality analysis. The results showed significant absolute and relative SDI-related inequalities in the global burden of childhood meningitis (Fig 5). Notably, deaths were disproportionately concentrated in countries with lower SDI. As indicated by the slope index of inequality, the gap in the death rate of childhood meningitis between the highest and lowest SDI countries decreased from 50.5 (95% CI: 47.3 to 53.6) per 100,000 population in 1990 to 10.4 (95% CI: 9.4 to 11.4) per 100,000 population in 2021. Additionally, the concentration index was −0.48 (95% CI: −0.55 to −0.4) in 1990 and −0.53 (95% CI: −0.6 to −0.45) in 2021, indicating a relative imbalance in the distribution of childhood meningitis burden across countries of different SDI levels.

## Discussion

Understanding the long-term trends of childhood meningitis is crucial for supporting clinical practice, monitoring epidemic strains with pandemic potential, and guiding healthcare service planning as well as the development of relevant drugs and vaccines [10]. This study provides the most up-to-date estimates of the burden and trends of childhood meningitis at global, regional, and national levels. Additionally, this study also presents a comprehensive assessment of the etiology composition of childhood meningitis. In 2021, approximately 1.33 million incident cases and 112,000 deaths due to meningitis were reported among children worldwide. From 1990 to 2021, both the global incidence and mortality rates of childhood meningitis have shown encouraging declines. However, the cross-country inequality analysis reveals a significant disparity, with low SDI countries disproportionately bearing the burden. Notably, children under 5 years of age, especially newborns, are particularly vulnerable to meningitis.

From 1990 to 2021, the global incidence of childhood meningitis decreased by 59.8%, and mortality decreased by 69.18%. This trend is largely attributed to the global promotion of vaccination programs, widespread use of antibiotics, and improvements in healthcare infrastructure worldwide. For example, the introduction of highly effective conjugate vaccines against Haemophilus influenzae type b (Hib), Streptococcus pneumoniae, and Neisseria meningitidis has significantly reduced the burden of meningitis caused by these pathogens [20,21]. According to WHO data, by 2021, global coverage of the third dose of Hib vaccine (Hib3) reached 71%, and the third dose of pneumococcal conjugate vaccine (PCV3) coverage attained 51% [22]. In the countries of the African meningitis belt, the incidence of suspected meningitis cases decreased by 57% among populations vaccinated with MenAfriVac (meningococcal group A conjugate vaccine) [23]. Additionally, the faster decrease in mortality relative to incidence further reflects advancements in the clinical management of affected children. However, as a typical preventable disease, childhood meningitis exhibited significantly higher incidence and mortality rates in underdeveloped regions such as Western Sub-Saharan Africa and Eastern Sub-Saharan Africa. This finding underscores the urgent need to strengthen medical services and preventive measures in these areas. In addition, Climatic factors in these regions also contribute to the high burden of childhood meningitis [24].

Consistent with previous research, the incidence and mortality rates of meningitis decreased with age from infancy to early adolescence [6,25]. Children under 5 years of age accounted for 76.2% of all cases and 81.1% of deaths among children, indicating that this age group bears the greatest burden of meningitis. Notably, the neonatal period exhibited especially higher incidence and mortality rates, likely attributed to the immaturity and compromised function of the immune system in newborns. Preterm birth and low birth weight are also recognized as risk factors for high meningitis morbidity and mortality [26–28]. A multicenter study from Turkey reported that the mortality rate of meningitis in preterm infants was three times higher than in full-term infants [29]. Additionally, the birthweight of non-surviving infants was significantly lower than that of surviving infants (1020g vs. 2210g; P < 0.001). The absence of specific clinical manifestations coupled with high rates of negative blood cultures makes the diagnosis of meningitis in newborns more complicated than in other age groups [30,31]. Delayed diagnosis and therapeutic interventions can lead to severe and long-term consequences, particularly for newborns during critical phases of neurodevelopment. Therefore, increased attention and funding for neonatal meningitis in high-burden regions are urgently needed.

The etiology of meningitis is diverse, with bacterial and viral pathogens being the leading infectious causes [6]. Early determination of the causative pathogens during hospital visits is vital to reduce unnecessary antibiotic use [8]. In 2021, the leading etiologies of childhood meningitis deaths globally were Streptococcus pneumoniae (17.01%), Neisseria meningitidis (14.03%), and Klebsiella pneumoniae (12.11%). Compared to 1990, the contribution of Neisseria meningitidis has significantly declined, whereas the proportions attributed to Streptococcus pneumoniae and Klebsiella pneumoniae have relatively increased. These shifts may reflect changes in vaccination strategies and evolving patterns of antibiotic resistance [32]. Notably, the highest proportion of Klebsiella pneumoniae-related deaths occurred in high-SDI regions, possibly linked to the prevalence of multidrug-resistant strains in healthcare settings there [33]. A study in Europe reported that 33% of countries documented carbapenem resistance percentages of Klebsiella pneumoniae reaching 25% in 2021, while 42% of countries reported third-generation cephalosporin resistance percentages exceeding 50% [34]. Notably, Group B Streptococcus (GBS) was the leading cause of neonatal meningitis-related deaths, exhibiting mortality rates significantly higher than other age groups. Neonatal GBS infections are classified into early-onset disease (EOD) and late-onset disease (LOD) based on age of onset [31]. Currently, prenatal screening and intrapartum antibiotic prophylaxis are the most commonly used methods for preventing neonatal GBS infections [35]. However, intrapartum antibiotic prophylaxis does not mitigate the risk of LOD. Therefore, the development of maternal vaccines targeting GBS may represent an important strategy for preventing both EOD and LOD [36].

Globally, the decline in the incidence and mortality rates of childhood meningitis is encouraging. However, our cross-country inequality analysis highlights a pronounced disparity in burden distribution, with low SDI countries and regions disproportionately affected. Given the constrained healthcare resources in these high-burden settings, policymakers in low-income countries must prioritize cost-effective interventions that maximize health benefits and align with their national strategic health plans [37]. Therefore, focusing on the primary prevention of childhood meningitis remains the most effective strategy to reduce morbidity and mortality in low- and middle-income countries. Strengthening immunization efforts, expanding access to vaccines, and developing new vaccines are critical components of meningitis prevention and control efforts [38]. Humanitarian and philanthropic organizations—including global health partnerships such as Gavi, the Vaccine Alliance—play a crucial role in supporting vaccine delivery and building local capacity, which are essential for alleviating the childhood meningitis burden in resource-limited settings. Additionally, robust national surveillance systems are needed to monitor vaccine effectiveness and generate accurate estimates of meningitis-related deaths and disease burden [39]. Meanwhile, raising public awareness of meningitis, developing affordable and reliable diagnostic methods, and providing comprehensive support and rehabilitation for children affected by meningitis sequelae represent important complementary strategies to further reduce the childhood meningitis burden [40,41].

### Limitations

The methodological updates in GBD 2021 have allowed us to comprehensively evaluate the burden of meningitis among children at global, regional, and national levels. However, our study still has several limitations like other similar GBD studies. First, the quality and availability of raw data vary considerably across countries, especially in underdeveloped regions, where inadequate health information systems may lead to incomplete and biased data. Second, the GBD study primarily relied on the modeling process for estimation, consequently, the choice of model and parameter settings may significantly affect the results. Third, GBD 2021 only assessed risk factors for neonatal meningitis and did not extend this analysis to all childhood age groups, but this would be an important subject of future research.

## Conclusions

Despite significant progress over the past three decades, childhood meningitis remains a major public health challenge, particularly in resource-limited settings. The leading etiologies of childhood meningitis globally were Streptococcus pneumoniae, Neisseria meningitidis, and Klebsiella pneumoniae. Targeted efforts are needed to improve vaccination coverage, enhance early detection and diagnosis, and strengthen meningitis surveillance.

## Supporting information

S1 File **S1 Fig.** The deaths attributable to childhood meningitis in 204 countries and territories. A, The death rate of childhood meningitis in 2021. B, The changes in death rate from 1990 to 2021. **S2 Fig.** Death rate of childhood meningitis by etiology and regions in 2021. **S3 Fig.** Deaths from neonatal meningitis attributed to level 4 risk factors: Number (A) and Percentage (B), in 2021. **S4 Fig.** The correlation between the SDI and incidence rate(A) and death rate(B) of childhood meningitis in 204 countries in 2021. **S1 Table.** Incidence of childhood meningitis in 1990 and 2021, with EAPC from 1990 to 2021. **S2 Table.** Incidence of childhood meningitis in 204 countries, 1990 and 2021, with EAPC from 1990 and 2021. **S3 Table.** Deaths of meningitis in 1990 and 2021, with EAPC from 1990 and 2021. **S4 Table.** Deaths of childhood meningitis in 204 countries, 1990 and 2021, with EAPC from 1990 to 2021. **S5 Table.** Prevalence of impairments attributed to childhood meningitis in 1990 and 2021. **S6 Table.** Etiology proportions (%) of childhood meningitis deaths by region in 2021. **S7 Table.** Etiology proportions (%) of childhood meningitis deaths by region in 1990. **S8 Table.** Global death counts and mortality rates of childhood meningitis by etiology, 1990 and 2021, with changes from 1990 to 2021. **S9 Table.** Incidence of childhood meningitis by age groups, 1990 and 2021, with EAPC from 1990 and 2021. S10 Table. Deaths of childhood meningitis by age group, 1990 and 2021, with EAPC from 1990 and 2021. S11 Table. Etiology proportions (%) of childhood meningitis deaths by age group, 2021. S12 Table. Incidence of meningitis among neonates (0–27 days) in 1990 and 2021, with EAPC from 1990 to 2021. S13 Table. Deaths of meningitis among neonates (0–27 days) in 1990 and 2021, with EAPC from 1990 to 2021. S14 Table. Incidence and death rate of meningitis among neonates in 204 countries, 1990 and 2021, with EAPC from 1990 to 2021. S15 Table. Death of childhood meningitis attributable to risk factors, in 1990 and 2021, with EAPC from 1990 to 2021).
(PDF)
