# Supplementary material for: Global, regional, and national burden of meningitis among children, 1990–2021: An analysis of the global burden of disease study 2021
Source: PLoS One. 2025 Jun 24;20(6):e0326992. doi: 10.1371/journal.pone.0326992 (PMC12186954; doi:10.1371/journal.pone.0326992)
Supplement: S1 File — S1 Fig. The deaths attributable to childhood meningitis in 204 countries and territories. A, The death rate of childhood meningitis in 2021. B, The changes in death rate from 1990 to 2021. S2 Fig. Death rate of childhood meningitis by etiology and regions in 2021. S3 Fig. Deaths from neonatal meningitis attributed to level 4 risk factors: Number (A) and Percentage (B), in 2021. S4 Fig. The correlation between the SDI and incidence rate(A) and death rate(B) of childhood meningitis in 204 countries in 2021. S1 Table. Incidence of childhood meningitis in 1990 and 2021, with EAPC from 1990 to 2021. S2 Table. Incidence of childhood meningitis in 204 countries, 1990 and 2021, with EAPC from 1990 and 2021. S3 Table. Deaths of meningitis in 1990 and 2021, with EAPC from 1990 and 2021. S4 Table. Deaths of childhood meningitis in 204 countries, 1990 and 2021, with EAPC from 1990 to 2021. S5 Table. Prevalence of impairments attributed to childhood meningitis in 1990 and 2021. S6 Table. Etiology proportions (%) of childhood meningitis deaths by region in 2021. S7 Table. Etiology proportions (%) of childhood meningitis deaths by region in 1990. S8 Table. Global death counts and mortality rates of childhood meningitis by etiology, 1990 and 2021, with changes from 1990 to 2021. S9 Table. Incidence of childhood meningitis by age groups, 1990 and 2021, with EAPC from 1990 and 2021. S10 Table. Deaths of childhood meningitis by age group, 1990 and 2021, with EAPC from 1990 and 2021. S11 Table. Etiology proportions (%) of childhood meningitis deaths by age group, 2021. S12 Table. Incidence of meningitis among neonates (0–27 days) in 1990 and 2021, with EAPC from 1990 to 2021. S13 Table. Deaths of meningitis among neonates (0–27 days) in 1990 and 2021, with EAPC from 1990 to 2021. S14 Table. Incidence and death rate of meningitis among neonates in 204 countries, 1990 and 2021, with EAPC from 1990 to 2021. S15 Table. Death of childhood meningitis attributable to risk factors, in 1990 and [file pone.0326992.s001.pdf]

Supplemental material

Global, regional, and national burden of meningitis among children, 1990–2021: An analysis of the global burden of disease study 2021

Supplemental Figures

S1 Fig. The deaths attributable to childhood meningitis in 204 countries and territories. A, The death rate of childhood meningitis in 2021. B, The changes in death rate from 1990 to 2021.

S2 Fig. Death rate of childhood meningitis by etiology and regions in 2021.

S3 Fig. Deaths from neonatal meningitis attributed to level 4 risk factors: Number (A) and Percentage (B), in 2021.

S4 Fig. The correlation between the SDI and incidence rate(A) and death rate(B) of childhood meningitis in 204 countries in 2021.

Supplemental Tables

S1 Table. Incidence of childhood meningitis in 1990 and 2021, with EAPC from 1990 to 2021.

S2 Table. Incidence of childhood meningitis in 204 countries, 1990 and 2021, with EAPC from 1990 and 2021.

S3 Table. Deaths of meningitis in 1990 and 2021, with EAPC from 1990 and 2021.

S4 Table. Deaths of childhood meningitis in 204 countries, 1990 and 2021, with EAPC from 1990 to 2021.

S5 Table. Prevalence of impairments attributed to childhood meningitis in 1990 and 2021.

S6 Table. Etiology proportions (%) of childhood meningitis deaths by region in 2021.

S7 Table. Etiology proportions (%) of childhood meningitis deaths by region in 1990.

S8 Table. Global death counts and mortality rates of childhood meningitis by etiology, 1990 and 2021, with changes from 1990 to 2021.

S9 Table. Incidence of childhood meningitis by age groups, 1990 and 2021, with EAPC from 1990 and 2021.

S10 Table. Deaths of childhood meningitis by age group, 1990 and 2021, with EAPC from 1990 and 2021.

S11 Table. Etiology proportions (%) of childhood meningitis deaths by age group, 2021.

S12 Table. Incidence of meningitis among neonates (0-27 days) in 1990 and 2021, with EAPC from 1990 to 2021.

S13 Table. Deaths of meningitis among neonates (0-27 days) in 1990 and 2021, with EAPC from 1990 to 2021.

S14 Table. Incidence and death rate of meningitis among neonates in 204 countries, 1990 and 2021, with EAPC from 1990 to 2021.

S15 Table. Death of childhood meningitis attributable to risk factors, in 1990 and 2021, with EAPC from 1990 to 2021

Supplemental Figures.

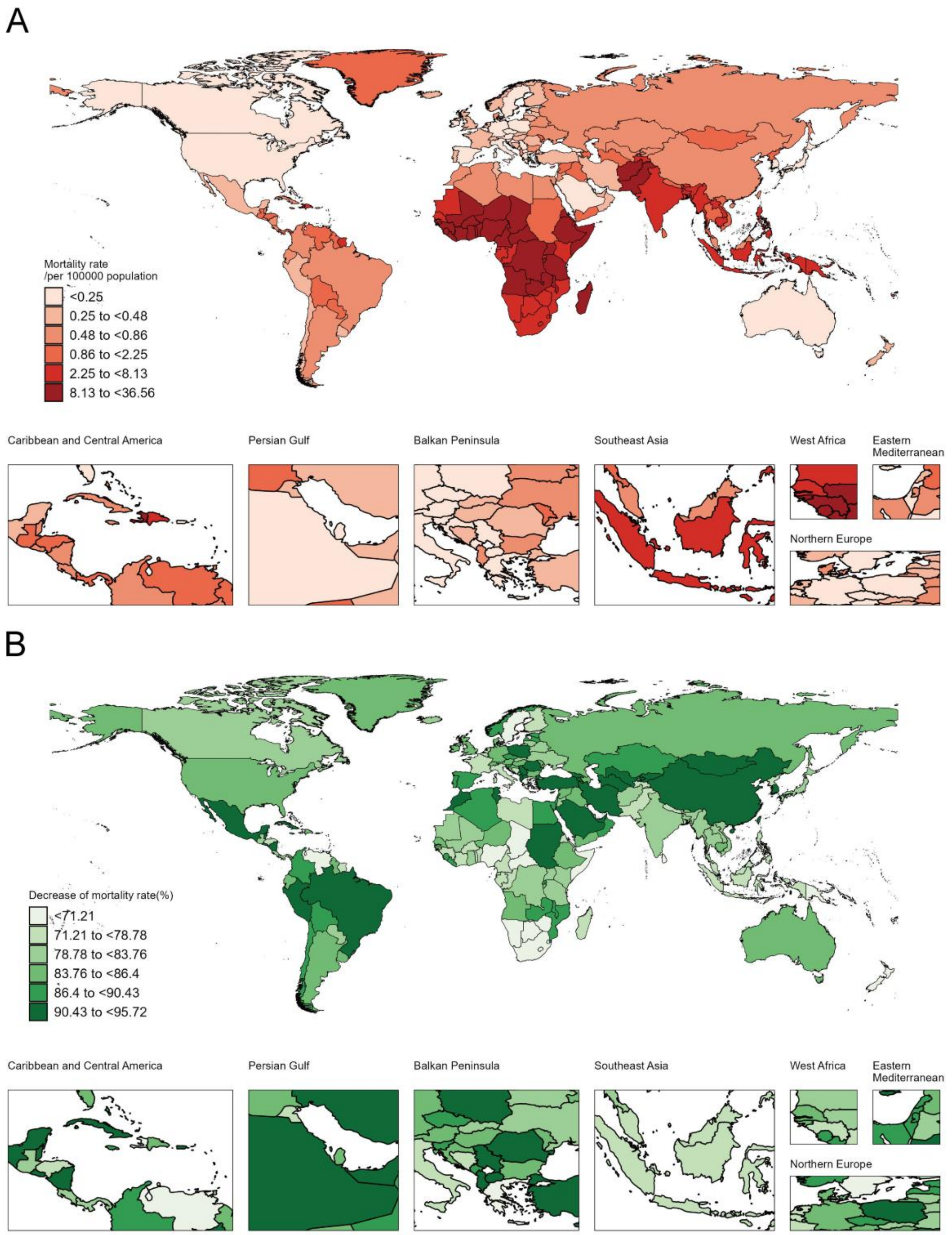

S1 Fig. The deaths attributable to childhood meningitis in 204 countries and territories.

A, The death rate of childhood meningitis in 2021. B, The changes in death rate from 1990 to 2021.

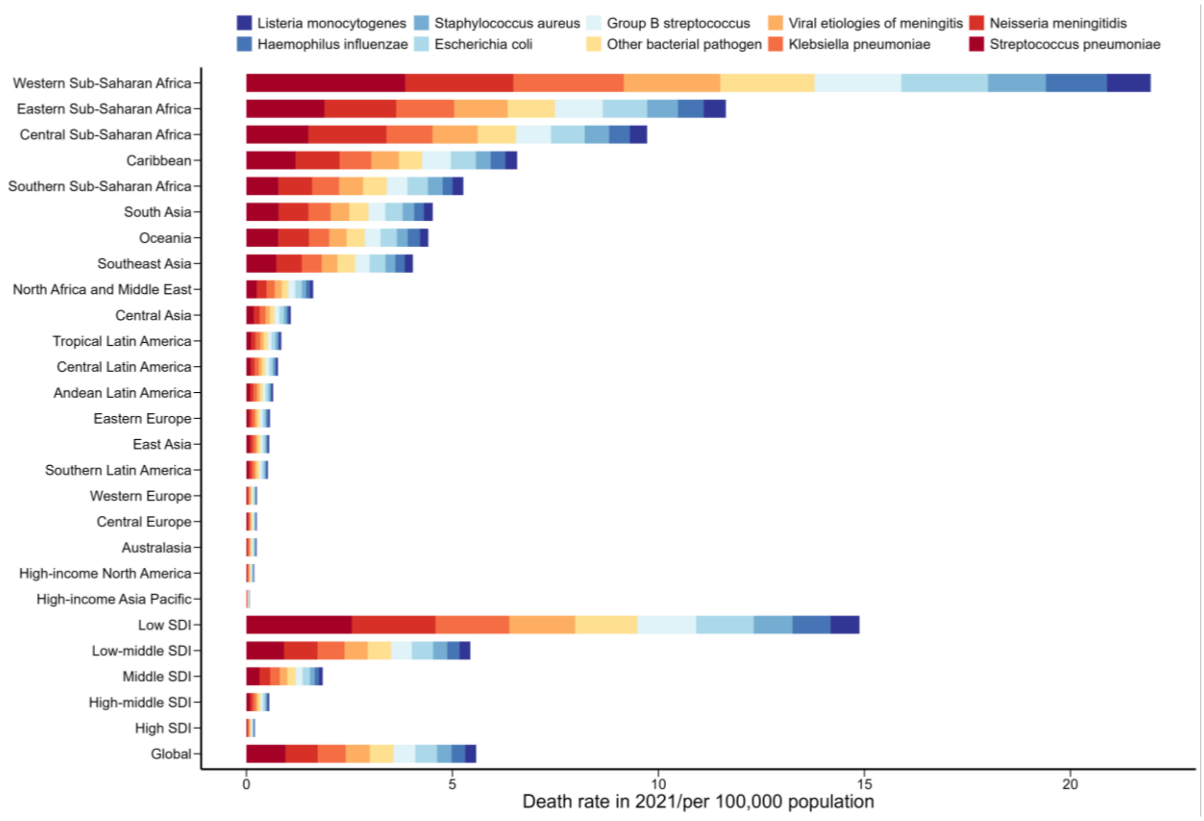

S2 Fig. Death rate of childhood meningitis by Etiology and regions in 2021.

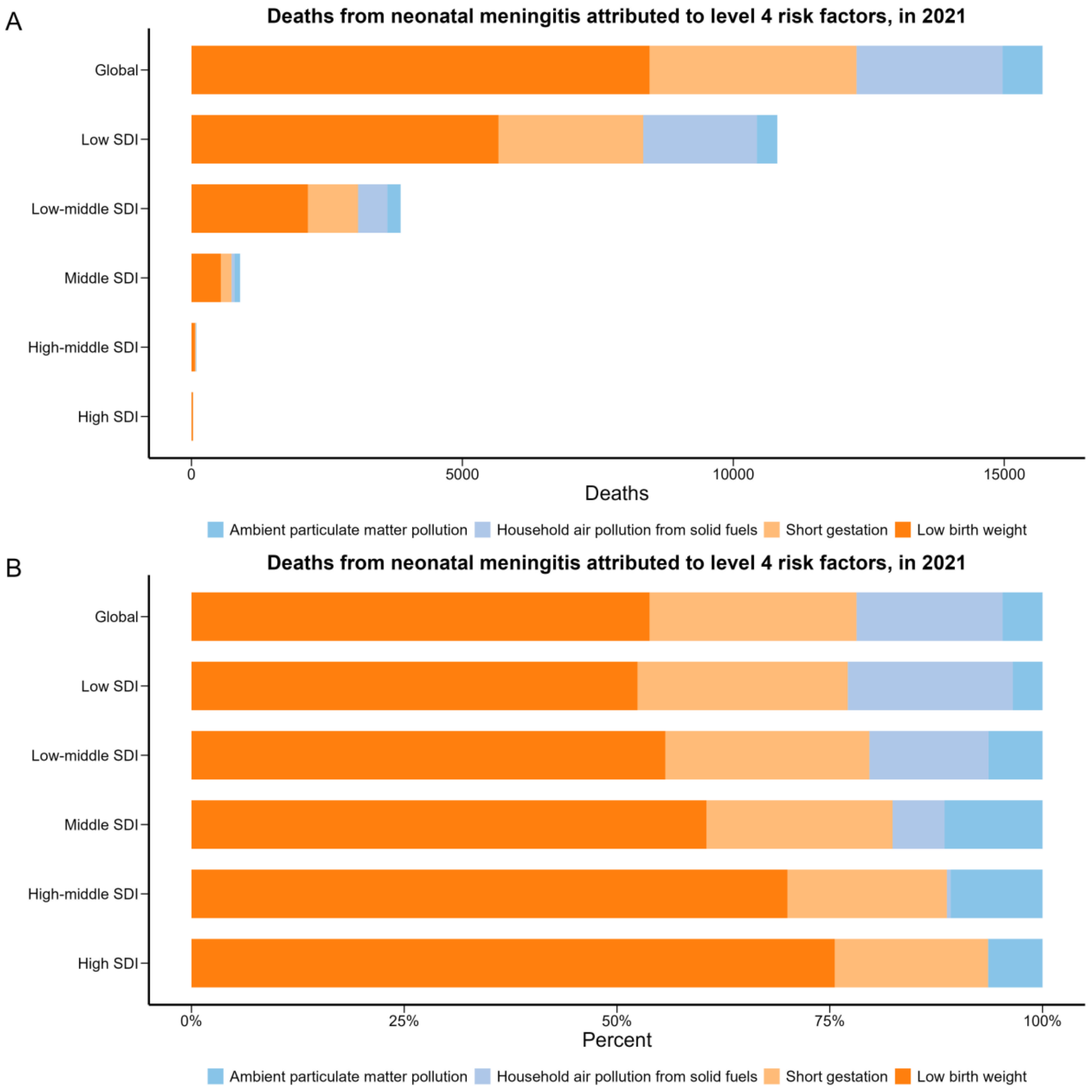

S3 Fig. Deaths from neonatal meningitis attributed to level 4 risk factors: Number (A) and Percentage (B), in 2021.

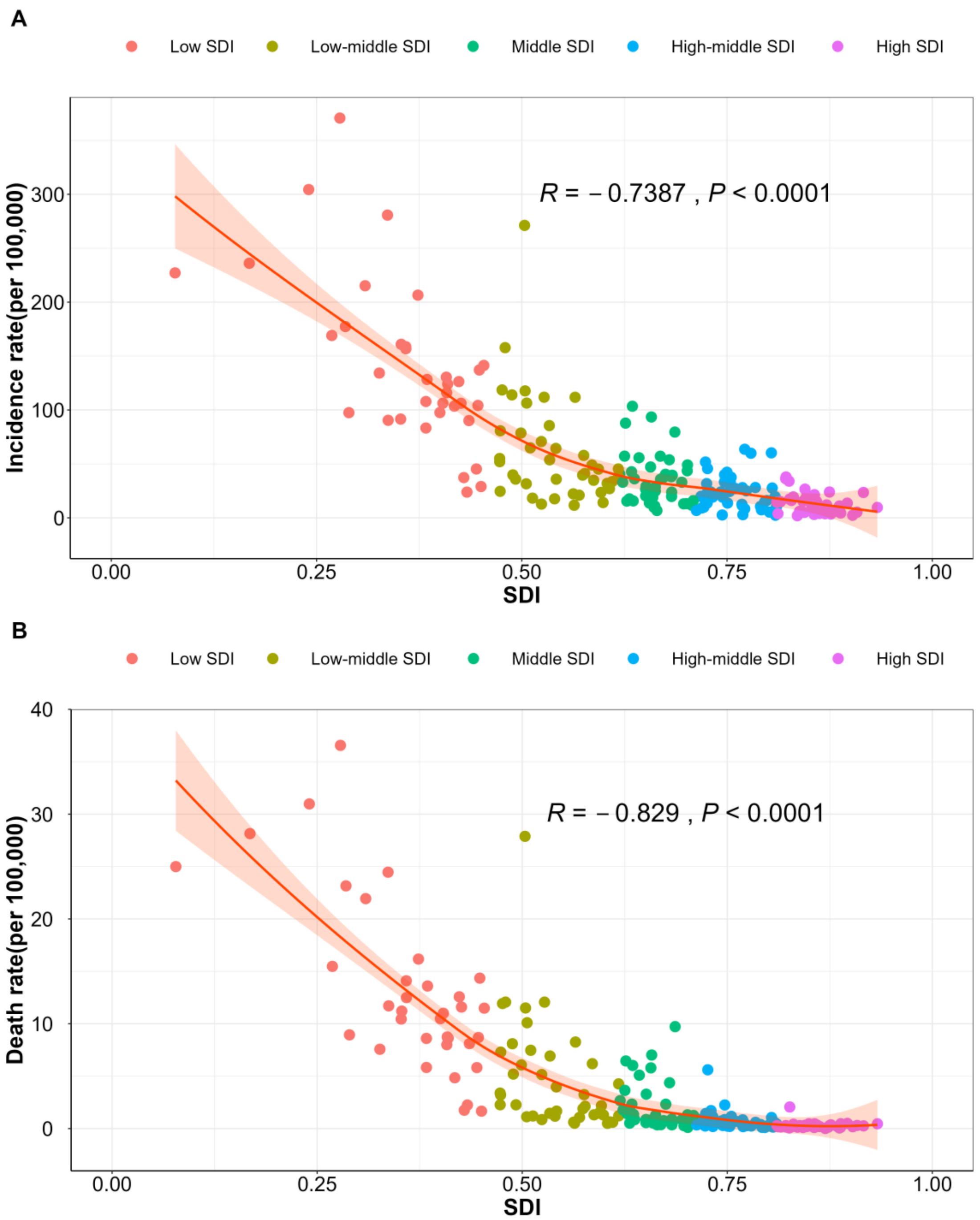

S4 Fig. The correlation between the SDI and incidence rate(A) and death rate(B) of childhood meningitis in 204 countries in 2021.

Supplemental Tables.

S1 Table. Incidence of childhood meningitis in 1990 and 2021, with EAPC from 1990 to 2021

Location	Incident cases in 1990	Rate in 1990 (per 100,000)	Incident cases in 2021	Rate in 2021 (per 100,000)	Changes of cases, 1990-2021	Changes of rates, 1990-2021	EAPC, 1990-2021
Global	2866040 (2297345 to 3486322)	164.8 (132.1 to 200.46)	1332734 (1107029 to 1577408)	66.24 (55.03 to 78.41)	-53.5% (-55.65 to -51.08)	-59.8% (-61.66 to -57.71)	-2.96% (-3.27 to -2.65)
**5 SDI quintiles regions**							
High SDI	48642 (35327 to 65068)	26.18 (19.01 to 35.02)	13837 (10207 to 18725)	8.02 (5.92 to 10.85)	-71.55% (-72.96 to -69.95)	-69.36% (-70.88 to -67.64)	-4.17% (-4.37 to -3.97)
High-middle SDI	169599 (131208 to 208821)	61.98 (47.95 to 76.32)	31634 (24082 to 41362)	13.7 (10.43 to 17.91)	-81.35% (-83.37 to -79.21)	-77.9% (-80.29 to -75.36)	-4.91% (-5.08 to -4.74)
Middle SDI	593090 (470190 to 724798)	102.75 (81.46 to 125.57)	161702 (128059 to 200532)	28.53 (22.59 to 35.38)	-72.74% (-74.67 to -70.62)	-72.24% (-74.21 to -70.08)	-4.08% (-4.35 to -3.81)
Low-middle SDI	975025 (788353 to 1179199)	206.52 (166.98 to 249.77)	400137 (331664 to 474276)	69.01 (57.2 to 81.79)	-58.96% (-60.92 to -56.86)	-66.59% (-68.18 to -64.87)	-3.56% (-3.88 to -3.23)
Low SDI	1077723 (867512 to 1302213)	470.8 (378.97 to 568.87)	724525 (607610 to 848982)	157.43 (132.02 to 184.47)	-32.77% (-36.03 to -29.12)	-66.56% (-68.18 to -64.74)	-3.72% (-3.99 to -3.45)
**GBD regions**							
Andean Latin America	7185 (5927 to 8517)	48.37 (39.91 to 57.34)	2025 (1601 to 2557)	11.19 (8.85 to 14.13)	-71.82% (-74.95 to -68.72)	-76.87% (-79.44 to -74.32)	-5.05% (-5.21 to -4.89)
Australasia	906 (700 to 1140)	19.75 (15.26 to 24.85)	406 (309 to 540)	7.08 (5.39 to 9.42)	-55.22% (-58.51 to -52.14)	-64.17% (-66.8 to -61.7)	-4.29% (-4.67 to -3.9)
Caribbean	20246 (16928 to 23701)	177.4 (148.33 to 207.68)	9191 (7774 to 10793)	79.89 (67.57 to 93.81)	-54.6% (-57.6 to -51.36)	-54.97% (-57.94 to -51.76)	-2.27% (-2.47 to -2.06)
Central Asia	27018 (22464 to 31930)	108.11 (89.89 to 127.77)	9838 (7680 to 12453)	35.55 (27.75 to 45)	-63.59% (-67.46 to -59.48)	-67.12% (-70.61 to -63.41)	-3.79% (-3.98 to -3.61)
Central Europe	11756 (9196 to 14842)	39.87 (31.19 to 50.34)	1741 (1314 to 2293)	9.84 (7.42 to 12.95)	-85.19% (-86.55 to -83.99)	-75.33% (-77.6 to -73.33)	-4.58% (-4.73 to -4.44)
Central Latin America	30324 (24449 to 36608)	47.1 (37.98 to 56.86)	7433 (5767 to 9548)	11.71 (9.08 to 15.04)	-75.49% (-78.45 to -72.28)	-75.14% (-78.15 to -71.89)	-4.6% (-4.98 to -4.22)
Central Sub-Saharan Africa	103348 (85318 to 123183)	408.51 (337.24 to 486.92)	70255 (59559 to 82168)	119.72 (101.5 to 140.02)	-32.02% (-37.41 to -26.78)	-70.69% (-73.02 to -68.43)	-4.32% (-4.78 to -3.87)

Location	Incident cases in 1990	Rate in 1990 (per 100,000)	Incident cases in 2021	Rate in 2021 (per 100,000)	Changes of cases, 1990-2021	Changes of rates, 1990-2021	EAPC, 1990-2021
East Asia	247493 (189870 to 307316)	75.04 (57.57 to 93.17)	27083 (19965 to 35895)	10.13 (7.47 to 13.43)	-89.06% (-90.98 to -86.98)	-86.5% (-88.87 to -83.94)	-6.75% (-6.96 to -6.54)
Eastern Europe	41331 (32507 to 51297)	80.31 (63.17 to 99.68)	7611 (5709 to 9880)	21.47 (16.11 to 27.87)	-81.58% (-83.48 to -79.59)	-73.26% (-76.02 to -70.37)	-3.8% (-4.27 to -3.32)
Eastern Sub-Saharan Africa	465475 (377267 to 561100)	513.94 (416.54 to 619.52)	236306 (199545 to 277313)	132.44 (111.83 to 155.42)	-49.23% (-52.36 to -45.81)	-74.23% (-75.82 to -72.49)	-4.64% (-4.88 to -4.4)
High-income Asia Pacific	6349 (4345 to 9001)	18.04 (12.34 to 25.57)	2259 (1596 to 3236)	10.08 (7.12 to 14.43)	-64.41% (-67.12 to -61.59)	-44.14% (-48.39 to -39.71)	-2.36% (-2.83 to -1.89)
High-income North America	16005 (11007 to 22192)	25.95 (17.85 to 35.98)	2845 (2175 to 3729)	4.34 (3.32 to 5.68)	-82.22% (-83.57 to -80.03)	-83.29% (-84.55 to -81.23)	-6.27% (-6.95 to -5.59)
North Africa and Middle East	119053 (96156 to 142327)	84.74 (68.45 to 101.31)	50036 (40256 to 62677)	27.29 (21.96 to 34.19)	-57.97% (-61.71 to -53.7)	-67.79% (-70.66 to -64.52)	-3.7% (-4.08 to -3.31)
Oceania	4726 (3924 to 5639)	176.34 (146.43 to 210.43)	4716 (3948 to 5596)	92.81 (77.69 to 110.15)	-0.21% (-6.7 to 6.12)	-47.37% (-50.79 to -44.03)	-2.66% (-2.83 to -2.49)
South Asia	849454 (684293 to 1040753)	196.01 (157.9 to 240.16)	331292 (270494 to 398008)	65.34 (53.35 to 78.5)	-61% (-62.73 to -59.17)	-66.67% (-68.15 to -65.11)	-3.56% (-3.9 to -3.21)
Southeast Asia	245979 (195740 to 303995)	144.06 (114.64 to 178.04)	62818 (50599 to 75759)	36.38 (29.31 to 43.88)	-74.46% (-75.95 to -72.92)	-74.74% (-76.21 to -73.22)	-4.36% (-4.63 to -4.09)
Southern Latin America	6573 (5453 to 7880)	44.04 (36.53 to 52.79)	1613 (1280 to 2045)	11.13 (8.83 to 14.11)	-75.46% (-77.96 to -72.93)	-74.73% (-77.31 to -72.12)	-4.49% (-4.69 to -4.29)
Southern Sub-Saharan Africa	23694 (19115 to 28652)	114.52 (92.39 to 138.49)	14634 (12183 to 17287)	60.81 (50.62 to 71.83)	-38.24% (-41.67 to -33.94)	-46.9% (-49.85 to -43.21)	-2.03% (-2.34 to -1.71)
Tropical Latin America	53284 (43457 to 64515)	99.39 (81.06 to 120.33)	13150 (10189 to 16977)	26.2 (20.3 to 33.82)	-75.32% (-78.72 to -71.71)	-73.64% (-77.27 to -69.78)	-5.34% (-5.72 to -4.97)
Western Europe	16108 (12076 to 20939)	22.68 (17 to 29.48)	4898 (3668 to 6519)	7.19 (5.39 to 9.57)	-69.59% (-70.87 to -68.29)	-68.3% (-69.63 to -66.94)	-4.02% (-4.2 to -3.85)
Western Sub-Saharan Africa	569732 (452176 to 701787)	648.31 (514.54 to 798.58)	472582 (395744 to 558761)	220.05 (184.27 to 260.17)	-17.05% (-21.86 to -11.09)	-66.06% (-68.03 to -63.62)	-3.59% (-3.93 to -3.25)

SDI, sociodemographic index; EAPC, estimated annual percentage change

S2 Table. Incidence of childhood meningitis in 204 countries, 1990 and 2021, with EAPC from 1990 and 2021.

Location	Incident cases in 1990	Rate in 1990 (per 100,000)	Incident cases in 2021	Rate in 2021 (per 100,000)	Changes of cases, 1990-2021	Changes of rates, 1990-2021	EAPC, 1990-2021
Afghanistan	21312 (17081 to 25989)	494.69 (396.49 to 603.27)	12842 (10568 to 15327)	90.43 (74.42 to 107.93)	-39.74% (-45.88% to -33.06%)	-81.72% (-83.58% to -79.69%)	-5.45% (-5.89% to -5%)
Albania	707 (578 to 862)	63.29 (51.74 to 77.18)	52 (40 to 67)	11.74 (9.01 to 15.13)	-92.63% (-93.65% to -91.59%)	-81.45% (-84.02% to -78.82%)	-6.01% (-6.26% to -5.76%)
Algeria	6017 (4807 to 7352)	56.1 (44.82 to 68.55)	2520 (1884 to 3328)	18.95 (14.16 to 25.02)	-58.12% (-63.76% to -51.4%)	-66.23% (-70.78% to -60.82%)	-3.11% (-3.38% to -2.85%)
American Samoa	15 (12 to 19)	80.95 (62.67 to 101.16)	7 (5 to 10)	51.74 (38.39 to 68.72)	-52.35% (-58% to -46.56%)	-36.09% (-43.66% to -28.31%)	-1.62% (-1.83% to -1.42%)
Andorra	1 (1 to 2)	11.29 (8.13 to 15.82)	0 (0 to 1)	3.74 (2.64 to 5.15)	-64.55% (-67.68% to -60.49%)	-66.87% (-69.79% to -63.07%)	-4.13% (-4.36% to -3.9%)
Angola	26744 (21778 to 32313)	567.24 (461.9 to 685.34)	21546 (18070 to 25170)	141.33 (118.53 to 165.1)	-19.44% (-27.23% to -8.92%)	-75.09% (-77.5% to -71.83%)	-4.99% (-5.29% to -4.7%)
Antigua and Barbuda	12 (10 to 15)	66.96 (54.1 to 79.97)	7 (6 to 9)	42.29 (32.87 to 53.07)	-41.32% (-47.84% to -34.83%)	-36.84% (-43.87% to -29.87%)	-1.92% (-2.09% to -1.75%)
Argentina	4707 (3957 to 5607)	46.44 (39.05 to 55.32)	1249 (993 to 1583)	12.26 (9.75 to 15.54)	-73.47% (-76.43% to -70.4%)	-73.6% (-76.54% to -70.54%)	-4.32% (-4.52% to -4.11%)
Armenia	714 (546 to 918)	68.42 (52.35 to 88.03)	291 (203 to 411)	49.05 (34.3 to 69.45)	-59.3% (-64.11% to -53.03%)	-28.32% (-36.79% to -17.28%)	-1% (-1.32% to -0.68%)
Australia	526 (416 to 649)	13.88 (11 to 17.15)	240 (181 to 318)	5.06 (3.81 to 6.69)	-54.3% (-59.72% to -48.78%)	-63.57% (-67.89% to -59.17%)	-3.48% (-3.61% to -3.35%)
Austria	456 (391 to 524)	33.81 (29.01 to 38.86)	145 (113 to 194)	11.21 (8.73 to 14.99)	-68.11% (-73.33% to -60.59%)	-66.85% (-72.27% to -59.03%)	-3.33% (-3.52% to -3.14%)
Azerbaijan	2059 (1690 to 2456)	84.83 (69.64 to 101.18)	779 (603 to 998)	33 (25.53 to 42.28)	-62.16% (-67.04% to -57.29%)	-61.1% (-66.11% to -56.09%)	-3.2% (-3.64% to -2.77%)
Bahamas	53 (43 to 64)	65.61 (53.25 to 79.75)	22 (17 to 30)	27.65 (20.57 to 36.7)	-57.59% (-63.28% to -51.86%)	-57.86% (-63.52% to -52.17%)	-2.95% (-3.3% to -2.6%)
Bahrain	105 (82 to 135)	64.5 (50.51 to 82.57)	73 (51 to 100)	24.44 (17.35 to 33.84)	-31.14% (-40.15% to -20.98%)	-62.11% (-67.07% to -56.52%)	-3.71% (-4.03% to -3.39%)
Bangladesh	53812 (43808 to 65772)	110.02 (89.56 to 134.47)	16424 (13023 to 20243)	35.89 (28.46 to 44.23)	-69.48% (-72.6% to -65.95%)	-67.38% (-70.71% to -63.61%)	-3.37% (-3.86% to -2.87%)
Barbados	45 (36 to 54)	71.61 (58.33 to 86.42)	16 (12 to 21)	33.24 (25.3 to 44)	-64.95% (-68.8% to -60.86%)	-53.58% (-58.68% to -48.17%)	-2.9% (-3.16% to -2.65%)
Belarus	1721 (1376 to 2115)	71.58 (57.23 to 87.98)	443 (332 to 585)	28.09 (21.02 to 37.04)	-74.24% (-77.27% to -71.01%)	-60.75% (-65.38% to -55.84%)	-2.63% (-2.91% to -2.36%)
Belgium	510 (374 to 682)	28.25 (20.72 to 37.75)	206 (150 to 282)	10.79 (7.82 to 14.75)	-59.57% (-64.4% to -55.71%)	-61.82% (-66.38% to -58.16%)	-3.24% (-3.8% to -2.68%)
Belize	63 (53 to 75)	77.06 (64.14 to 91.46)	41 (32 to 54)	33.67 (25.6 to 43.83)	-34.29% (-44.3% to -22.26%)	-56.31% (-62.97% to -48.31%)	-2.89% (-3.1% to -2.68%)

Location	Incident cases in 1990	Rate in 1990 (per 100,000)	Incident cases in 2021	Rate in 2021 (per 100,000)	Changes of cases, 1990-2021	Changes of rates, 1990-2021	EAPC, 1990-2021
Benin	12942 (10511 to 15701)	534.41 (434.02 to 648.31)	12555 (10580 to 14705)	206.48 (174 to 241.84)	-2.99% (-11.14% to 6.16%)	-61.36% (-64.61% to -57.72%)	-3.41% (-3.72% to -3.1%)
Bermuda	7 (6 to 9)	59.64 (46.77 to 74.32)	3 (2 to 4)	36.36 (26.09 to 48.08)	-56.86% (-62.2% to -51.34%)	-39.04% (-46.59% to -31.24%)	-1.78% (-1.98% to -1.58%)
Bhutan	491 (398 to 595)	187.09 (151.61 to 227.09)	103 (86 to 122)	55.03 (46.12 to 64.96)	-79% (-81.48% to -76.26%)	-70.59% (-74.06% to -66.74%)	-4.24% (-4.45% to -4.02%)
Bolivia (Plurinational State of)	2554 (2053 to 3124)	95.08 (76.46 to 116.31)	493 (409 to 595)	14.15 (11.74 to 17.06)	-80.68% (-83.27% to -77.78%)	-85.11% (-87.11% to -82.88%)	-6.23% (-6.44% to -6.02%)
Bosnia and Herzegovina	374 (293 to 470)	34.13 (26.7 to 42.92)	86 (63 to 117)	17.57 (12.95 to 23.82)	-76.96% (-79.63% to -74.34%)	-48.54% (-54.5% to -42.67%)	-2.33% (-2.52% to -2.14%)
Botswana	574 (472 to 685)	97.24 (79.93 to 116.07)	389 (325 to 453)	55.72 (46.51 to 64.93)	-32.23% (-37.61% to -25.62%)	-42.7% (-47.25% to -37.11%)	-1.92% (-2.36% to -1.47%)
Brazil	52185 (42544 to 63229)	100.46 (81.9 to 121.72)	12836 (9945 to 16592)	26.64 (20.64 to 34.43)	-75.4% (-78.82% to -71.75%)	-73.48% (-77.17% to -69.55%)	-5.34% (-5.72% to -4.97%)
Brunei Darussalam	14 (11 to 18)	15.49 (12.18 to 19.74)	10 (7 to 13)	10.3 (7.92 to 13.58)	-30.57% (-36.76% to -24.51%)	-33.52% (-39.45% to -27.72%)	-1.61% (-2.03% to -1.19%)
Bulgaria	646 (512 to 815)	37.19 (29.49 to 46.95)	122 (94 to 161)	12.53 (9.58 to 16.44)	-81.06% (-82.72% to -79.24%)	-66.3% (-69.27% to -63.07%)	-3.81% (-4.23% to -3.39%)
Burkina Faso	29756 (24049 to 35922)	630.55 (509.62 to 761.21)	18394 (15098 to 22130)	177.34 (145.56 to 213.36)	-38.19% (-44.9% to -30%)	-71.88% (-74.93% to -68.15%)	-4.25% (-5.01% to -3.49%)
Burundi	10686 (8767 to 12809)	407.7 (334.48 to 488.68)	5708 (4854 to 6665)	97.51 (82.92 to 113.84)	-46.58% (-51.98% to -40.74%)	-76.08% (-78.5% to -73.47%)	-4.64% (-5.13% to -4.15%)
Cabo Verde	306 (242 to 370)	194.58 (154.01 to 235.47)	122 (96 to 154)	85.52 (66.96 to 107.64)	-59.99% (-65.21% to -54.59%)	-56.05% (-61.78% to -50.11%)	-3.2% (-3.53% to -2.86%)
Cambodia	7518 (6056 to 9158)	161.3 (129.92 to 196.49)	1257 (1026 to 1500)	24.57 (20.05 to 29.32)	-83.28% (-85.03% to -81.41%)	-84.77% (-86.36% to -83.07%)	-6.27% (-6.73% to -5.81%)
Cameroon	19356 (15847 to 23363)	396.47 (324.59 to 478.56)	21248 (17785 to 24837)	157.78 (132.07 to 184.43)	9.78% (-1.12% to 20.29%)	-60.2% (-64.16% to -56.39%)	-3.14% (-3.58% to -2.69%)
Canada	787 (607 to 1031)	13.68 (10.56 to 17.92)	457 (351 to 618)	7.4 (5.69 to 10.02)	-41.95% (-46.08% to -38.01%)	-45.9% (-49.75% to -42.23%)	-2.11% (-2.26% to -1.95%)
Central African Republic	6226 (5199 to 7205)	509.24 (425.23 to 589.3)	4915 (4219 to 5594)	215.19 (184.74 to 244.94)	-21.07% (-27.31% to -13.78%)	-57.74% (-61.08% to -53.84%)	-2.65% (-2.98% to -2.31%)
Chad	14161 (11566 to 16941)	483.92 (395.25 to 578.9)	27441 (23063 to 32263)	304.39 (255.83 to 357.89)	93.77% (76.09% to 112.89%)	-37.1% (-42.84% to -30.89%)	-1.69% (-1.93% to -1.46%)
Chile	1571 (1260 to 1915)	39.55 (31.72 to 48.22)	304 (238 to 388)	8.33 (6.52 to 10.63)	-80.64% (-82.64% to -78.69%)	-78.95% (-81.12% to -76.82%)	-5.24% (-5.46% to -5.01%)
China	241228 (184712 to 299400)	75.77 (58.02 to 94.04)	25376 (18702 to 33676)	9.77 (7.2 to 12.97)	-89.48% (-91.33% to -87.46%)	-87.1% (-89.37% to -84.62%)	-6.91% (-7.14% to -6.68%)
Colombia	6465 (5364 to 7593)	55.43 (45.99 to 65.1)	1454 (1122 to 1863)	13.7 (10.58 to 17.55)	-77.51% (-80.69% to -73.95%)	-75.29% (-78.78% to -71.37%)	-4.93% (-5.15% to -4.71%)
Comoros	671 (552 to 800)	315.51 (259.61 to 376.01)	285 (239 to 336)	118.57 (99.64 to 140.02)	-57.57% (-61.5% to -52.88%)	-62.42% (-65.9% to -58.27%)	-3.29% (-3.54% to -3.04%)

Location	Incident cases in 1990	Rate in 1990 (per 100,000)	Incident cases in 2021	Rate in 2021 (per 100,000)	Changes of cases, 1990-2021	Changes of rates, 1990-2021	EAPC, 1990-2021
Congo	2611 (2173 to 3088)	247.99 (206.38 to 293.26)	1607 (1342 to 1906)	83.29 (69.56 to 98.81)	-38.46% (-44.91% to -31.54%)	-66.41% (-69.93% to -62.64%)	-3.99% (-4.62% to -3.36%)
Cook Islands	5 (3 to 6)	70.55 (51.1 to 94.78)	2 (2 to 3)	59.87 (41.06 to 84.84)	-51.27% (-56% to -45.89%)	-15.15% (-23.39% to -5.78%)	-0.93% (-1.21% to -0.64%)
Costa Rica	334 (262 to 414)	29.72 (23.3 to 36.83)	134 (101 to 179)	13.14 (9.9 to 17.55)	-60% (-64.89% to -54.36%)	-55.79% (-61.2% to -49.56%)	-2.57% (-2.67% to -2.47%)
Coted'Ivoire	17057 (13860 to 20580)	299.07 (243.01 to 360.85)	12290 (10290 to 14525)	106.21 (88.92 to 125.52)	-27.95% (-34.42% to -20.78%)	-64.49% (-67.68% to -60.95%)	-3.21% (-3.69% to -2.73%)
Croatia	332 (256 to 430)	33.6 (25.9 to 43.56)	96 (72 to 124)	16 (12.14 to 20.85)	-71.19% (-73.31% to -68.2%)	-52.38% (-55.89% to -47.44%)	-2.47% (-2.69% to -2.25%)
Cuba	3525 (2932 to 4214)	140.77 (117.09 to 168.29)	1015 (754 to 1325)	57.12 (42.44 to 74.55)	-71.2% (-76.03% to -66.53%)	-59.42% (-66.23% to -52.84%)	-2.7% (-3.07% to -2.33%)
Cyprus	22 (17 to 28)	11.19 (8.77 to 13.92)	4 (3 to 6)	1.98 (1.48 to 2.63)	-80.48% (-83.25% to -77.25%)	-82.34% (-84.84% to -79.41%)	-5.54% (-5.78% to -5.3%)
Czechia	669 (509 to 873)	30.36 (23.12 to 39.63)	277 (199 to 384)	16.13 (11.57 to 22.38)	-58.62% (-63.56% to -54.22%)	-46.88% (-53.21% to -41.22%)	-2.64% (-3% to -2.27%)
Democratic People's Republic of [illegible]	3786 (3006 to 4602)	63.64 (50.53 to 77.36)	1002 (779 to 1301)	21 (16.31 to 27.26)	-73.53% (-77.31% to -69.26%)	-67.01% (-71.72% to -61.69%)	-3.7% (-3.97% to -3.42%)
Democratic Republic of the Congo	66017 (54764 to 78323)	372.9 (309.34 to 442.41)	40980 (34453 to 48791)	107.84 (90.67 to 128.4)	-37.93% (-44.25% to -32%)	-71.08% (-74.03% to -68.32%)	-4.34% (-4.88% to -3.8%)
Denmark	426 (326 to 540)	48.23 (36.85 to 61.16)	130 (96 to 181)	13.62 (10.06 to 18.99)	-69.49% (-73.62% to -64.68%)	-71.75% (-75.58% to -67.3%)	-4.41% (-4.73% to -4.1%)
Djibouti	456 (368 to 550)	261.62 (211.57 to 315.67)	471 (395 to 556)	114.01 (95.7 to 134.46)	3.41% (-5.25% to 13.95%)	-56.42% (-60.07% to -51.98%)	-3.06% (-3.58% to -2.54%)
Dominica	16 (13 to 19)	64.49 (52.33 to 78.54)	5 (4 to 7)	40 (31.76 to 51.57)	-65.8% (-69.34% to -61.79%)	-37.98% (-44.41% to -30.7%)	-1.71% (-1.85% to -1.56%)
Dominican Republic	3965 (3232 to 4721)	147.1 (119.91 to 175.15)	1190 (987 to 1393)	40.49 (33.6 to 47.42)	-69.99% (-73.28% to -66.25%)	-72.47% (-75.49% to -69.04%)	-3.85% (-4.16% to -3.54%)
Ecuador	1393 (1166 to 1631)	36.04 (30.18 to 42.19)	491 (387 to 627)	9.69 (7.64 to 12.37)	-64.73% (-68.6% to -59.65%)	-73.11% (-76.06% to -69.24%)	-5% (-5.3% to -4.69%)
Egypt	17057 (13999 to 20687)	76.9 (63.11 to 93.26)	9964 (7638 to 13108)	27.04 (20.72 to 35.57)	-41.58% (-51.02% to -30.93%)	-64.84% (-70.52% to -58.43%)	-3.25% (-3.59% to -2.92%)
El Salvador	1137 (934 to 1367)	52.7 (43.27 to 63.36)	212 (159 to 288)	11.65 (8.75 to 15.84)	-81.37% (-85.06% to -77.44%)	-77.89% (-82.27% to -73.23%)	-5.29% (-5.68% to -4.91%)
Equatorial Guinea	692 (574 to 818)	351.66 (291.34 to 415.58)	547 (452 to 651)	93.49 (77.3 to 111.24)	-21.02% (-29% to -12.12%)	-73.41% (-76.1% to -70.42%)	-4.73% (-4.98% to -4.48%)
Eritrea	5557 (4612 to 6671)	349.1 (289.71 to 419.06)	2682 (2243 to 3157)	106.24 (88.84 to 125.04)	-51.73% (-55.88% to -46.44%)	-69.57% (-72.18% to -66.23%)	-3.82% (-4.16% to -3.49%)
Estonia	244 (196 to 301)	69.93 (56.26 to 86.21)	58 (41 to 79)	26.63 (19.08 to 36.45)	-76.43% (-79.81% to -72.88%)	-61.93% (-67.39% to -56.19%)	-2.97% (-3.18% to -2.76%)
Eswatini	428 (348 to 514)	110.89 (90.19 to 133.24)	202 (172 to 234)	48.97 (41.69 to 56.69)	-52.76% (-58.25% to -45.69%)	-55.84% (-60.97% to -49.23%)	-2.28% (-3.01% to -1.54%)

Location	Incident cases in 1990	Rate in 1990 (per 100,000)	Incident cases in 2021	Rate in 2021 (per 100,000)	Changes of cases, 1990-2021	Changes of rates, 1990-2021	EAPC, 1990-2021
Ethiopia	177718 (142562 to 216370)	729.43 (585.14 to 888.08)	69566 (56878 to 82763)	156.86 (128.25 to 186.61)	-60.86% (-63.63% to -57.91%)	-78.5% (-80.02% to -76.88%)	-5.52% (-5.82% to -5.21%)
Fiji	265 (215 to 317)	94.22 (76.26 to 112.74)	110 (85 to 137)	40.38 (31.32 to 50.16)	-58.51% (-62% to -53.43%)	-57.15% (-60.75% to -51.9%)	-3.05% (-3.54% to -2.56%)
Finland	243 (178 to 328)	25.14 (18.43 to 33.98)	88 (62 to 126)	10.35 (7.29 to 14.85)	-63.86% (-68.85% to -59.25%)	-58.83% (-64.51% to -53.58%)	-2.99% (-3.48% to -2.5%)
France	2029 (1557 to 2614)	17.32 (13.29 to 22.32)	703 (542 to 924)	6.06 (4.67 to 7.96)	-65.35% (-68.07% to -62.27%)	-65.04% (-67.78% to -61.93%)	-3.31% (-3.54% to -3.07%)
Gabon	1057 (880 to 1262)	259.43 (215.97 to 309.73)	661 (552 to 783)	103.48 (86.31 to 122.53)	-37.44% (-43.47% to -31.02%)	-60.11% (-63.96% to -56.02%)	-3.11% (-3.4% to -2.82%)
Gambia	1756 (1438 to 2136)	380.7 (311.82 to 463.09)	1232 (1019 to 1453)	124.04 (102.55 to 146.22)	-29.83% (-36.68% to -21.63%)	-67.42% (-70.6% to -63.61%)	-4.09% (-4.48% to -3.7%)
Georgia	775 (622 to 951)	56.63 (45.48 to 69.45)	141 (109 to 183)	19.22 (14.86 to 24.9)	-81.75% (-83.45% to -80.14%)	-66.06% (-69.21% to -63.06%)	-3.29% (-3.55% to -3.03%)
Germany	1272 (982 to 1634)	9.83 (7.58 to 12.62)	282 (213 to 378)	2.36 (1.78 to 3.16)	-77.84% (-80.39% to -74.3%)	-76.02% (-78.78% to -72.19%)	-3.85% (-4.11% to -3.6%)
Ghana	22388 (18186 to 27462)	333.33 (270.77 to 408.87)	14402 (12109 to 17088)	111.79 (93.99 to 132.64)	-35.67% (-41.76% to -27.96%)	-66.46% (-69.64% to -62.44%)	-4.09% (-4.34% to -3.84%)
Greece	469 (360 to 591)	23.2 (17.81 to 29.19)	103 (78 to 141)	7.39 (5.56 to 10.08)	-78.04% (-82.39% to -72.93%)	-68.15% (-74.45% to -60.73%)	-3.6% (-3.91% to -3.3%)
Greenland	16 (13 to 19)	110.55 (88.19 to 133.55)	2 (2 to 3)	17.28 (13.43 to 21.52)	-87.08% (-88.55% to -85.42%)	-84.37% (-86.14% to -82.35%)	-5.74% (-6.2% to -5.28%)
Grenada	23 (19 to 28)	69.46 (56.89 to 83.15)	8 (6 to 10)	35.77 (26.57 to 45.97)	-66.35% (-71.12% to -61.42%)	-48.49% (-55.81% to -40.96%)	-2.19% (-2.43% to -1.96%)
Guam	31 (24 to 40)	74.99 (58.1 to 96.16)	22 (17 to 28)	60.25 (45.58 to 77.27)	-29.54% (-34.92% to -23.22%)	-19.67% (-25.8% to -12.46%)	-0.85% (-1.01% to -0.69%)
Guatemala	1924 (1596 to 2273)	47.37 (39.3 to 55.98)	870 (688 to 1104)	17.63 (13.93 to 22.38)	-54.78% (-61.7% to -47.62%)	-62.78% (-68.48% to -56.89%)	-3.46% (-3.63% to -3.29%)
Guinea	18797 (15280 to 22750)	683.09 (555.27 to 826.72)	16970 (14704 to 19579)	280.7 (243.22 to 323.85)	-9.72% (-18.25% to 0.47%)	-58.91% (-62.79% to -54.27%)	-2.76% (-2.92% to -2.59%)
Guinea-Bissau	3115 (2539 to 3759)	645.71 (526.35 to 779.33)	1445 (1220 to 1691)	160.89 (135.83 to 188.27)	-53.61% (-57.76% to -48.36%)	-75.08% (-77.31% to -72.27%)	-4.85% (-5.46% to -4.24%)
Guyana	211 (176 to 252)	71.93 (59.71 to 85.57)	57 (44 to 74)	26.55 (20.52 to 34.46)	-73.21% (-76.73% to -68.98%)	-63.09% (-67.94% to -57.27%)	-3.59% (-3.82% to -3.36%)
Haiti	10001 (8215 to 11914)	368.64 (302.8 to 439.14)	5968 (5065 to 6995)	137.1 (116.36 to 160.7)	-40.33% (-46.25% to -33.99%)	-62.81% (-66.5% to -58.85%)	-2.8% (-2.97% to -2.62%)
Honduras	929 (745 to 1121)	42.04 (33.72 to 50.74)	598 (479 to 745)	18.24 (14.62 to 22.73)	-35.63% (-42.64% to -26.94%)	-56.6% (-61.33% to -50.75%)	-2.16% (-2.58% to -1.74%)
Hungary	713 (554 to 908)	33.47 (25.98 to 42.63)	147 (108 to 200)	10.59 (7.78 to 14.4)	-79.38% (-81.67% to -77.02%)	-68.36% (-71.87% to -64.73%)	-3.97% (-4.15% to -3.78%)
Iceland	9 (7 to 11)	13.42 (10.54 to 17.14)	2 (2 to 3)	3.48 (2.6 to 4.56)	-72.38% (-75.85% to -69.15%)	-74.06% (-77.32% to -71.02%)	-4.59% (-4.78% to -4.39%)

Location	Incident cases in 1990	Rate in 1990 (per 100,000)	Incident cases in 2021	Rate in 2021 (per 100,000)	Changes of cases, 1990-2021	Changes of rates, 1990-2021	EAPC, 1990-2021
India	618249 (499700 to 762019)	189.34 (153.04 to 233.38)	211998 (171937 to 256326)	57.86 (46.93 to 69.96)	-65.71% (-67.34% to -63.9%)	-69.44% (-70.89% to -67.83%)	-3.94% (-4.35% to -3.54%)
Indonesia	130635 (100785 to 164154)	192.86 (148.79 to 242.34)	31729 (25036 to 38827)	47.15 (37.21 to 57.7)	-75.71% (-76.96% to -74.12%)	-75.55% (-76.8% to -73.95%)	-4.6% (-4.87% to -4.33%)
Iran (Islamic Republic of)	12638 (10056 to 15440)	49.79 (39.62 to 60.82)	2622 (1979 to 3422)	13 (9.81 to 16.96)	-79.25% (-82.22% to -76.25%)	-73.9% (-77.64% to -70.12%)	-3.93% (-4.28% to -3.58%)
Iraq	6729 (5519 to 8066)	81.7 (67.01 to 97.94)	3330 (2626 to 4196)	24.74 (19.5 to 31.17)	-50.51% (-57.38% to -42.89%)	-69.72% (-73.92% to -65.06%)	-3.91% (-4.36% to -3.47%)
Ireland	196 (152 to 252)	19.94 (15.47 to 25.62)	58 (43 to 79)	5.81 (4.3 to 7.95)	-70.41% (-73.89% to -67.05%)	-70.85% (-74.28% to -67.53%)	-4.35% (-4.85% to -3.85%)
Israel	198 (156 to 250)	12.89 (10.16 to 16.32)	63 (48 to 82)	2.41 (1.82 to 3.13)	-67.99% (-72.78% to -63.78%)	-81.33% (-84.12% to -78.87%)	-5.28% (-5.48% to -5.08%)
Italy	2380 (1561 to 3453)	25.79 (16.91 to 37.41)	409 (307 to 555)	5.38 (4.04 to 7.3)	-82.81% (-85.37% to -79.88%)	-79.12% (-82.23% to -75.56%)	-5.98% (-6.56% to -5.41%)
Jamaica	598 (492 to 705)	71.64 (58.96 to 84.45)	230 (174 to 300)	39.47 (29.87 to 51.36)	-61.47% (-66.33% to -54.72%)	-44.9% (-51.84% to -35.23%)	-2.24% (-2.46% to -2.03%)
Japan	4829 (3129 to 7115)	20.92 (13.55 to 30.82)	1851 (1307 to 2634)	11.98 (8.46 to 17.05)	-61.67% (-65.76% to -57.03%)	-42.7% (-48.81% to -35.76%)	-2.56% (-3.27% to -1.85%)
Jordan	645 (510 to 812)	39.51 (31.19 to 49.72)	825 (629 to 1077)	22.7 (17.32 to 29.66)	27.76% (15.37% to 41.57%)	-42.56% (-48.13% to -36.35%)	-2.2% (-2.39% to -2%)
Kazakhstan	4578 (3799 to 5627)	88.09 (73.11 to 108.28)	1723 (1310 to 2250)	31.75 (24.13 to 41.47)	-62.36% (-67.03% to -57.72%)	-63.96% (-68.43% to -59.51%)	-3.56% (-3.92% to -3.19%)
Kenya	27199 (21788 to 33212)	243.49 (195.05 to 297.33)	13201 (10894 to 15798)	70.72 (58.36 to 84.64)	-51.47% (-55.06% to -47.53%)	-70.95% (-73.11% to -68.6%)	-3.94% (-4.23% to -3.64%)
Kiribati	110 (90 to 131)	372.47 (303.86 to 443.9)	47 (39 to 56)	111.85 (92.61 to 134.46)	-57.28% (-61.52% to -52.23%)	-69.97% (-72.96% to -66.42%)	-3.94% (-4.19% to -3.69%)
Kuwait	208 (158 to 269)	37.49 (28.44 to 48.59)	164 (117 to 220)	19.34 (13.84 to 26.02)	-21.32% (-28.08% to -15.07%)	-48.41% (-52.85% to -44.32%)	-2.52% (-2.72% to -2.31%)
Kyrgyzstan	2207 (1860 to 2562)	131.56 (110.85 to 152.72)	726 (557 to 942)	31.94 (24.5 to 41.42)	-67.09% (-72.48% to -61.63%)	-75.72% (-79.7% to -71.7%)	-4.54% (-4.66% to -4.41%)
Lao People's Democratic Republic	3986 (3325 to 4647)	216.27 (180.39 to 252.15)	914 (778 to 1056)	39.78 (33.86 to 46.01)	-77.08% (-79.46% to -74.36%)	-81.61% (-83.51% to -79.42%)	-5.74% (-6.18% to -5.31%)
Latvia	410 (326 to 498)	72.13 (57.34 to 87.48)	58 (43 to 78)	19.47 (14.31 to 26.13)	-85.91% (-87.68% to -83.83%)	-73.01% (-76.4% to -69.03%)	-4.65% (-5.06% to -4.23%)
Lebanon	443 (337 to 565)	42.39 (32.25 to 54.07)	244 (173 to 337)	19.06 (13.54 to 26.34)	-45.05% (-51.38% to -37.49%)	-55.03% (-60.21% to -48.85%)	-2.67% (-2.86% to -2.48%)
Lesotho	699 (581 to 840)	102.46 (85.06 to 123.07)	409 (353 to 482)	64.9 (55.94 to 76.44)	-41.49% (-47.51% to -33.96%)	-36.66% (-43.17% to -28.51%)	-1.42% (-1.85% to -0.99%)
Liberia	8651 (6904 to 10727)	765.43 (610.84 to 949.15)	2001 (1691 to 2363)	91.56 (77.38 to 108.09)	-76.86% (-79.64% to -73.29%)	-88.04% (-89.47% to -86.19%)	-6.9% (-7.12% to -6.67%)
Libya	700 (549 to 868)	38.67 (30.3 to 47.95)	204 (151 to 275)	13.65 (10.1 to 18.47)	-70.94% (-75.11% to -67.3%)	-64.71% (-69.79% to -60.3%)	-3.63% (-3.96% to -3.3%)

Location	Incident cases in 1990	Rate in 1990 (per 100,000)	Incident cases in 2021	Rate in 2021 (per 100,000)	Changes of cases, 1990-2021	Changes of rates, 1990-2021	EAPC, 1990-2021
Lithuania	584 (468 to 716)	70.3 (56.29 to 86.2)	88 (64 to 115)	21.46 (15.76 to 28.31)	-85.01% (-86.91% to -82.74%)	-69.47% (-73.34% to -64.85%)	-3.02% (-3.26% to -2.77%)
Luxembourg	14 (11 to 18)	20.78 (15.93 to 27.03)	6 (5 to 9)	6.18 (4.58 to 8.5)	-54.47% (-59.91% to -49.75%)	-70.28% (-73.83% to -67.2%)	-3.83% (-4.05% to -3.62%)
Madagascar	18511 (15168 to 22354)	339.28 (278.01 to 409.72)	11454 (9788 to 13451)	97.62 (83.42 to 114.64)	-38.12% (-44.66% to -31.31%)	-71.23% (-74.27% to -68.06%)	-3.98% (-4.35% to -3.61%)
Malawi	36759 (30054 to 43766)	807.95 (660.58 to 961.96)	10423 (8850 to 12142)	128.3 (108.93 to 149.45)	-71.64% (-74.69% to -67.47%)	-84.12% (-85.83% to -81.78%)	-6.23% (-6.46% to -6.01%)
Malaysia	3025 (2405 to 3756)	46.02 (36.59 to 57.14)	1827 (1390 to 2382)	23.99 (18.26 to 31.29)	-39.61% (-47.63% to -33.54%)	-47.87% (-54.79% to -42.62%)	-2.16% (-2.46% to -1.85%)
Maldives	48 (38 to 59)	45.84 (36.59 to 55.96)	20 (15 to 28)	20.31 (15.03 to 27.52)	-57.75% (-64.76% to -49.58%)	-55.69% (-63.04% to -47.13%)	-2.33% (-2.68% to -1.98%)
Mali	27909 (22641 to 34146)	675.8 (548.23 to 826.81)	19568 (16094 to 22993)	169.04 (139.03 to 198.63)	-29.89% (-36.67% to -21.89%)	-74.99% (-77.41% to -72.13%)	-4.99% (-5.5% to -4.48%)
Malta	13 (10 to 17)	15.1 (11.73 to 19.15)	3 (3 to 4)	5.35 (4.11 to 6.76)	-74.06% (-76.54% to -71.58%)	-64.54% (-67.92% to -61.14%)	-4.66% (-5.44% to -3.87%)
Marshall Islands	18 (15 to 22)	82.75 (66.2 to 101.9)	7 (5 to 9)	39.59 (31.47 to 49.25)	-61.93% (-64.76% to -58.55%)	-52.16% (-55.71% to -47.9%)	-2.73% (-3.09% to -2.37%)
Mauritania	2288 (1887 to 2771)	247.51 (204.09 to 299.73)	1457 (1195 to 1739)	78.62 (64.46 to 93.87)	-36.33% (-41.19% to -30.96%)	-68.24% (-70.66% to -65.56%)	-4.1% (-4.68% to -3.51%)
Mauritius	147 (119 to 179)	44.47 (35.97 to 54.28)	41 (31 to 54)	19.9 (15.12 to 26.02)	-71.88% (-74.85% to -68.57%)	-55.25% (-59.97% to -49.98%)	-2.91% (-3.09% to -2.73%)
Mexico	13407 (10297 to 16920)	40.12 (30.81 to 50.64)	2170 (1583 to 2973)	6.77 (4.94 to 9.27)	-83.82% (-86.38% to -81.07%)	-83.14% (-85.81% to -80.27%)	-5.98% (-6.59% to -5.36%)
Micronesia (Federated States of)	44 (35 to 54)	95.41 (76.05 to 116.79)	11 (8 to 14)	34.84 (26.86 to 44.16)	-75.67% (-78.37% to -72.8%)	-63.49% (-67.53% to -59.17%)	-3.68% (-3.9% to -3.46%)
Monaco	1 (0 to 1)	14.84 (10.82 to 19.62)	0 (0 to 0)	5.26 (3.86 to 7.15)	-49.94% (-55.29% to -44.41%)	-64.56% (-68.35% to -60.64%)	-3.5% (-3.81% to -3.19%)
Mongolia	1898 (1543 to 2284)	210.91 (171.5 to 253.79)	412 (311 to 550)	37.93 (28.59 to 50.59)	-78.29% (-82.16% to -73.76%)	-82.01% (-85.23% to -78.27%)	-6.29% (-6.72% to -5.85%)
Montenegro	43 (32 to 57)	26.78 (19.93 to 35.47)	16 (12 to 23)	14.71 (10.54 to 20.85)	-62.13% (-65.62% to -57.32%)	-45.08% (-50.14% to -38.1%)	-2.39% (-2.67% to -2.11%)
Morocco	8598 (6996 to 10499)	87.86 (71.49 to 107.29)	2177 (1716 to 2756)	22.23 (17.52 to 28.14)	-74.68% (-77.92% to -71.28%)	-74.69% (-77.94% to -71.3%)	-4.74% (-5.08% to -4.4%)
Mozambique	30854 (25355 to 36988)	497.32 (408.68 to 596.18)	19152 (16079 to 22444)	134.25 (112.71 to 157.32)	-37.93% (-44.14% to -31.78%)	-73% (-75.71% to -70.33%)	-4.5% (-4.86% to -4.14%)
Myanmar	45988 (36550 to 57493)	311.23 (247.35 to 389.09)	8396 (7019 to 9863)	53.77 (44.95 to 63.17)	-81.74% (-83.97% to -79.28%)	-82.72% (-84.83% to -80.39%)	-5.42% (-5.99% to -4.84%)
Namibia	600 (494 to 724)	99.81 (82.22 to 120.53)	374 (314 to 446)	45.32 (38.09 to 54.07)	-37.61% (-42.83% to -31.23%)	-54.59% (-58.39% to -49.95%)	-2.99% (-3.7% to -2.29%)
Nauru	4 (3 to 5)	100.94 (80.78 to 123.48)	2 (2 to 3)	57.18 (46.38 to 70.2)	-46.6% (-50.57% to -41.77%)	-43.35% (-47.56% to -38.23%)	-2.33% (-2.92% to -1.73%)

Location	Incident cases in 1990	Rate in 1990 (per 100,000)	Incident cases in 2021	Rate in 2021 (per 100,000)	Changes of cases, 1990-2021	Changes of rates, 1990-2021	EAPC, 1990-2021
Nepal	7278 (5675 to 9178)	86.38 (67.36 to 108.93)	2200 (1714 to 2783)	23.84 (18.58 to 30.16)	-69.77% (-72.9% to -66.41%)	-72.4% (-75.26% to -69.33%)	-3.77% (-3.94% to -3.6%)
Netherlands	418 (330 to 521)	15.35 (12.09 to 19.13)	119 (94 to 150)	4.45 (3.52 to 5.61)	-71.45% (-74.69% to -67.73%)	-70.99% (-74.28% to -67.21%)	-4.82% (-5.26% to -4.38%)
New Zealand	380 (281 to 501)	47.51 (35.12 to 62.6)	165 (126 to 221)	16.85 (12.79 to 22.53)	-56.5% (-60.57% to -52.33%)	-64.54% (-67.86% to -61.15%)	-5.15% (-5.86% to -4.44%)
Nicaragua	1719 (1433 to 2079)	94.39 (78.68 to 114.16)	252 (197 to 323)	12.75 (9.93 to 16.29)	-85.31% (-87.57% to -81.9%)	-86.49% (-88.57% to -83.35%)	-6.84% (-7.07% to -6.62%)
Niger	60378 (48119 to 75981)	1486.06 (1184.34 to 1870.09)	30132 (25032 to 36589)	236.07 (196.11 to 286.66)	-50.09% (-55.17% to -43.1%)	-84.11% (-85.73% to -81.89%)	-6.69% (-7.16% to -6.23%)
Nigeria	292581 (228658 to 362448)	747.82 (584.44 to 926.4)	275487 (226833 to 328728)	271.18 (223.28 to 323.59)	-5.84% (-12.17% to 1.67%)	-63.74% (-66.18% to -60.84%)	-3.17% (-3.54% to -2.79%)
Niue	1 (0 to 1)	78.61 (60.53 to 98.22)	0 (0 to 0)	45.66 (35.57 to 59.73)	-72.13% (-74.64% to -69.53%)	-41.92% (-47.15% to -36.51%)	-2.9% (-3.31% to -2.49%)
North Macedonia	286 (232 to 349)	54.29 (44 to 66.3)	45 (34 to 60)	13.69 (10.23 to 18.4)	-84.32% (-86.94% to -81.43%)	-74.78% (-79% to -70.13%)	-4.09% (-4.33% to -3.85%)
Northern Mariana Islands	10 (8 to 14)	85.41 (64.1 to 111.85)	7 (5 to 10)	63.58 (45.64 to 85.23)	-31.03% (-38.83% to -23.93%)	-25.55% (-33.97% to -17.89%)	-1.27% (-1.5% to -1.05%)
Norway	370 (269 to 491)	46.37 (33.66 to 61.54)	217 (154 to 301)	23.51 (16.62 to 32.59)	-41.34% (-46.04% to -37.01%)	-49.3% (-53.37% to -45.57%)	-3.4% (-3.92% to -2.88%)
Oman	383 (299 to 488)	45.56 (35.54 to 58.07)	306 (217 to 426)	25.04 (17.76 to 34.8)	-20.01% (-30.58% to -8.98%)	-45.03% (-52.3% to -37.46%)	-2.44% (-2.66% to -2.22%)
Pakistan	169624 (136442 to 206371)	344.47 (277.08 to 419.1)	100567 (83336 to 118901)	117.7 (97.53 to 139.16)	-40.71% (-46.11% to -34.33%)	-65.83% (-68.94% to -62.16%)	-3.23% (-3.48% to -2.98%)
Palau	4 (3 to 5)	92.71 (74.78 to 114.36)	1 (1 to 1)	32.69 (24.47 to 42.96)	-74.77% (-77.55% to -71.26%)	-64.74% (-68.63% to -59.84%)	-3.63% (-3.89% to -3.36%)
Palestine	605 (487 to 743)	62.45 (50.31 to 76.76)	328 (247 to 435)	17.59 (13.22 to 23.31)	-45.69% (-52.83% to -37.8%)	-71.83% (-75.54% to -67.74%)	-4.36% (-4.75% to -3.98%)
Panama	398 (317 to 478)	47.76 (37.99 to 57.38)	184 (144 to 231)	15.93 (12.48 to 20)	-53.88% (-60.02% to -46.99%)	-66.65% (-71.1% to -61.67%)	-3.58% (-3.73% to -3.42%)
Papua New Guinea	3569 (2973 to 4270)	209.94 (174.86 to 251.15)	4057 (3411 to 4791)	103.57 (87.07 to 122.32)	13.67% (5.53% to 21.88%)	-50.66% (-54.2% to -47.1%)	-2.93% (-3.08% to -2.78%)
Paraguay	1099 (908 to 1327)	65.83 (54.37 to 79.48)	314 (242 to 403)	15.65 (12.05 to 20.1)	-71.41% (-74.78% to -68.09%)	-76.23% (-79.02% to -73.46%)	-5.01% (-5.38% to -4.64%)
Peru	3238 (2640 to 3872)	39 (31.8 to 46.65)	1040 (802 to 1340)	10.91 (8.41 to 14.04)	-67.88% (-71.6% to -63.45%)	-72.04% (-75.28% to -68.19%)	-4.33% (-4.54% to -4.12%)
Philippines	25325 (19957 to 30957)	100.44 (79.15 to 122.78)	9914 (8090 to 12122)	29.16 (23.79 to 35.66)	-60.85% (-64.04% to -57.69%)	-70.97% (-73.33% to -68.62%)	-3.57% (-3.86% to -3.29%)
Poland	3524 (2489 to 4740)	36.79 (25.99 to 49.49)	229 (179 to 290)	3.89 (3.05 to 4.93)	-93.5% (-94.35% to -92.24%)	-89.42% (-90.81% to -87.38%)	-7.2% (-7.54% to -6.86%)
Portugal	378 (308 to 460)	17.89 (14.56 to 21.72)	36 (27 to 46)	2.65 (2 to 3.41)	-90.47% (-91.86% to -88.89%)	-85.2% (-87.35% to -82.74%)	-6.53% (-7.03% to -6.03%)

Location	Incident cases in 1990	Rate in 1990 (per 100,000)	Incident cases in 2021	Rate in 2021 (per 100,000)	Changes of cases, 1990-2021	Changes of rates, 1990-2021	EAPC, 1990-2021
Puerto Rico	561 (437 to 706)	56.32 (43.92 to 70.86)	151 (108 to 215)	34 (24.34 to 48.48)	-73.06% (-76.18% to -68.19%)	-39.63% (-46.63% to -28.71%)	-1.54% (-1.84% to -1.24%)
Qatar	53 (40 to 69)	42.49 (31.92 to 55.01)	96 (69 to 129)	19.37 (13.94 to 26.17)	80.09% (62.64% to 97.7%)	-54.41% (-58.82% to -49.95%)	-2.89% (-3.05% to -2.72%)
Republic of Korea	1445 (1135 to 1853)	12.71 (9.98 to 16.3)	374 (259 to 537)	6.15 (4.26 to 8.85)	-74.13% (-78.77% to -69.63%)	-51.58% (-60.26% to -43.16%)	-1.9% (-2.56% to -1.23%)
Republic of Moldova	1219 (1020 to 1456)	98.63 (82.55 to 117.81)	116 (88 to 145)	22.17 (16.92 to 27.85)	-90.5% (-91.81% to -89.28%)	-77.52% (-80.62% to -74.64%)	-4.01% (-4.3% to -3.72%)
Romania	3021 (2471 to 3632)	54.26 (44.38 to 65.23)	345 (261 to 462)	11.47 (8.67 to 15.35)	-88.57% (-90.25% to -86.66%)	-78.87% (-81.96% to -75.32%)	-4.43% (-4.6% to -4.25%)
Russian Federation	27595 (21552 to 34247)	79.53 (62.11 to 98.7)	5132 (3845 to 6683)	19.68 (14.75 to 25.63)	-81.4% (-83.5% to -79.35%)	-75.25% (-78.05% to -72.52%)	-4.04% (-4.61% to -3.46%)
Rwanda	16881 (13620 to 20457)	497.54 (401.43 to 602.93)	4481 (3746 to 5246)	90.15 (75.36 to 105.55)	-73.46% (-76.18% to -70.67%)	-81.88% (-83.74% to -79.98%)	-6.77% (-7.32% to -6.23%)
Saint Kitts and Nevis	12 (10 to 15)	88.31 (74.14 to 104.2)	4 (3 to 5)	37.57 (29.05 to 47.36)	-70.33% (-74.36% to -65.69%)	-57.45% (-63.23% to -50.8%)	-2.99% (-3.29% to -2.68%)
Saint Lucia	39 (32 to 46)	76.05 (61.98 to 90.14)	11 (8 to 14)	37.39 (28.61 to 47.85)	-71.68% (-75.15% to -67.77%)	-50.84% (-56.86% to -44.05%)	-2.5% (-2.74% to -2.27%)
Saint Vincent and the Grenadines	35 (29 to 42)	85.05 (71.79 to 101.12)	9 (7 to 12)	36.2 (27.92 to 46.99)	-74.15% (-77.71% to -70.33%)	-57.43% (-63.29% to -51.14%)	-3.1% (-3.25% to -2.94%)
Samoa	64 (51 to 78)	89.53 (71.7 to 109.92)	36 (28 to 46)	45.15 (34.73 to 57.78)	-43.43% (-48.77% to -37.28%)	-49.57% (-54.34% to -44.1%)	-2.47% (-2.75% to -2.19%)
San Marino	1 (1 to 1)	16.87 (12.48 to 22.46)	0 (0 to 0)	5.01 (3.61 to 6.96)	-68.15% (-72.07% to -63.89%)	-70.32% (-73.98% to -66.35%)	-3.89% (-4.01% to -3.76%)
Sao Tome and Principe	76 (61 to 93)	134.88 (107.98 to 163.51)	25 (19 to 31)	31.56 (25 to 39.85)	-67.86% (-71.6% to -63.9%)	-76.6% (-79.32% to -73.72%)	-5.23% (-5.65% to -4.81%)
Saudi Arabia	2471 (1911 to 3113)	37.71 (29.15 to 47.5)	1133 (785 to 1597)	14.98 (10.37 to 21.11)	-54.13% (-61.69% to -46%)	-60.26% (-66.82% to -53.23%)	-3.55% (-3.84% to -3.25%)
Senegal	17376 (13904 to 21186)	475.88 (380.78 to 580.23)	8295 (6994 to 9717)	130.41 (109.96 to 152.76)	-52.26% (-56.81% to -46.75%)	-72.6% (-75.2% to -69.43%)	-4.8% (-5.25% to -4.35%)
Serbia	654 (514 to 826)	30.17 (23.7 to 38.09)	128 (92 to 177)	9.64 (6.96 to 13.36)	-80.43% (-83.11% to -77.75%)	-68.04% (-72.41% to -63.66%)	-4.76% (-5.17% to -4.34%)
Seychelles	26 (22 to 30)	109.25 (92.12 to 126.46)	6 (4 to 7)	23.98 (18.79 to 30.13)	-78.34% (-81.32% to -75.07%)	-78.05% (-81.07% to -74.73%)	-4.95% (-5.95% to -3.93%)
Sierra Leone	14309 (11467 to 17543)	789.41 (632.6 to 967.84)	5675 (4680 to 6584)	158.69 (130.87 to 184.11)	-60.34% (-65.22% to -55.03%)	-79.9% (-82.37% to -77.21%)	-5.87% (-6.24% to -5.51%)
Singapore	61 (49 to 76)	9.39 (7.54 to 11.73)	25 (19 to 33)	3.08 (2.39 to 4.01)	-58.9% (-64.58% to -52.3%)	-67.14% (-71.68% to -61.86%)	-2.9% (-3.54% to -2.26%)
Slovakia	467 (352 to 608)	35.26 (26.59 to 45.9)	125 (91 to 169)	14.55 (10.66 to 19.78)	-73.35% (-75.53% to -70.32%)	-58.75% (-62.13% to -54.07%)	-3.24% (-3.42% to -3.06%)
Slovenia	132 (94 to 178)	31.81 (22.76 to 42.99)	48 (34 to 68)	15.34 (10.76 to 21.65)	-63.59% (-67.75% to -60.16%)	-51.78% (-57.29% to -47.25%)	-1.47% (-1.8% to -1.13%)

Location	Incident cases in 1990	Rate in 1990 (per 100,000)	Incident cases in 2021	Rate in 2021 (per 100,000)	Changes of cases, 1990-2021	Changes of rates, 1990-2021	EAPC, 1990-2021
Solomon Islands	148 (118 to 182)	94.92 (75.78 to 116.9)	97 (76 to 123)	37.3 (29.33 to 47.43)	-34.38% (-40.31% to -28.04%)	-60.71% (-64.26% to -56.91%)	-3.31% (-3.81% to -2.8%)
Somalia	15702 (12643 to 18944)	403.07 (324.55 to 486.3)	23461 (20133 to 27050)	227.13 (194.91 to 261.87)	49.41% (35.25% to 64.21%)	-43.65% (-48.99% to -38.07%)	-1.61% (-1.9% to -1.33%)
South Africa	15436 (12456 to 18684)	113.39 (91.5 to 137.25)	8179 (6535 to 10022)	53.79 (42.98 to 65.91)	-47.02% (-50.54% to -42.98%)	-52.56% (-55.72% to -48.95%)	-2.4% (-2.71% to -2.09%)
South Sudan	15118 (12491 to 17818)	576.13 (476 to 678.99)	15920 (13463 to 18687)	370.67 (313.46 to 435.09)	5.3% (-4.39% to 15.06%)	-35.66% (-41.58% to -29.7%)	-1.32% (-1.44% to -1.19%)
Spain	1130 (892 to 1416)	14.42 (11.38 to 18.06)	187 (139 to 244)	2.88 (2.15 to 3.77)	-83.48% (-85.57% to -81.14%)	-80.03% (-82.55% to -77.19%)	-4.86% (-5.12% to -4.6%)
Sri Lanka	3233 (2574 to 4002)	58.43 (46.52 to 72.32)	2226 (1661 to 2962)	43.61 (32.54 to 58.03)	-31.16% (-39.07% to -22.45%)	-25.37% (-33.95% to -15.94%)	-1.42% (-1.69% to -1.15%)
Sudan	15675 (12656 to 18634)	176.27 (142.32 to 209.54)	5942 (4757 to 7379)	35.82 (28.67 to 44.48)	-62.09% (-67.23% to -56.03%)	-79.68% (-82.43% to -76.43%)	-5.15% (-5.67% to -4.63%)
Suriname	133 (111 to 154)	101.74 (85.43 to 118.46)	61 (49 to 76)	42.74 (34.28 to 53.2)	-53.79% (-59.68% to -47.66%)	-57.99% (-63.34% to -52.41%)	-2.98% (-3.13% to -2.83%)
Sweden	375 (269 to 509)	24.3 (17.45 to 32.97)	200 (138 to 284)	11 (7.58 to 15.62)	-46.61% (-51.6% to -41.35%)	-54.71% (-58.95% to -50.25%)	-2.56% (-2.94% to -2.19%)
Switzerland	380 (286 to 493)	32.86 (24.73 to 42.69)	128 (90 to 174)	9.61 (6.74 to 13.07)	-66.28% (-71.07% to -61.77%)	-70.75% (-74.91% to -66.84%)	-3.98% (-4.28% to -3.69%)
Syrian Arab Republic	5814 (4795 to 6882)	98.17 (80.97 to 116.21)	1208 (930 to 1524)	32.98 (25.37 to 41.61)	-79.22% (-82.04% to -75.85%)	-66.4% (-70.96% to -60.96%)	-3.61% (-3.95% to -3.28%)
Taiwan (Province of China)	2479 (1807 to 3323)	45 (32.8 to 60.33)	705 (494 to 1016)	23.93 (16.76 to 34.49)	-71.55% (-74.96% to -68.06%)	-46.82% (-53.19% to -40.3%)	-2.83% (-3.21% to -2.45%)
Tajikistan	3831 (3206 to 4484)	164.99 (138.05 to 193.08)	2308 (1892 to 2743)	64.39 (52.78 to 76.52)	-39.76% (-45.51% to -33.35%)	-60.97% (-64.7% to -56.82%)	-3.2% (-3.33% to -3.07%)
Thailand	8944 (7277 to 10758)	53.05 (43.16 to 63.82)	2326 (1732 to 3065)	23.81 (17.73 to 31.38)	-74% (-78.44% to -69.45%)	-55.11% (-62.78% to -47.26%)	-2.71% (-2.86% to -2.55%)
Timor-Leste	669 (541 to 822)	201.09 (162.64 to 247.19)	236 (199 to 276)	45.29 (38.19 to 53.04)	-64.75% (-68.25% to -60.58%)	-77.48% (-79.72% to -74.82%)	-5.1% (-5.27% to -4.93%)
Togo	6511 (5389 to 7915)	369.39 (305.77 to 449.05)	3838 (3203 to 4580)	115.99 (96.78 to 138.42)	-41.05% (-45.75% to -34.38%)	-68.6% (-71.1% to -65.05%)	-4.14% (-4.68% to -3.59%)
Tokelau	1 (0 to 1)	90.22 (70.07 to 112.39)	0 (0 to 0)	79.51 (60.3 to 102.12)	-42.7% (-48.19% to -37.45%)	-11.87% (-20.3% to -3.79%)	-1.6% (-2.07% to -1.13%)
Tonga	70 (56 to 84)	167.53 (134.73 to 200.99)	34 (28 to 41)	87.82 (72.83 to 103.97)	-51.07% (-54.88% to -46.5%)	-47.58% (-51.66% to -42.68%)	-2.25% (-2.38% to -2.11%)
Trinidad and Tobago	243 (197 to 294)	59.85 (48.56 to 72.3)	76 (57 to 101)	27.96 (21 to 37.14)	-68.68% (-72.74% to -63.67%)	-53.28% (-59.33% to -45.81%)	-2.53% (-2.77% to -2.29%)
Tunisia	1650 (1296 to 2037)	53.13 (41.74 to 65.61)	546 (411 to 730)	19.76 (14.85 to 26.38)	-66.89% (-71.67% to -62.24%)	-62.82% (-68.19% to -57.61%)	-2.76% (-3.04% to -2.49%)
Turkey	10900 (8599 to 13269)	53.2 (41.97 to 64.76)	1261 (943 to 1643)	6.81 (5.09 to 8.87)	-88.43% (-90.51% to -86.09%)	-87.2% (-89.5% to -84.61%)	-7.35% (-7.77% to -6.93%)

Location	Incident cases in 1990	Rate in 1990 (per 100,000)	Incident cases in 2021	Rate in 2021 (per 100,000)	Changes of cases, 1990-2021	Changes of rates, 1990-2021	EAPC, 1990-2021
Turkmenistan	1802 (1497 to 2095)	120.06 (99.72 to 139.62)	580 (461 to 734)	38.09 (30.23 to 48.17)	-67.78% (-71.63% to -63.33%)	-68.27% (-72.06% to -63.88%)	-3.87% (-4.15% to -3.59%)
Tuvalu	6 (5 to 7)	160.15 (131.52 to 195.17)	2 (1 to 2)	41.1 (31.98 to 52.53)	-72.47% (-76.26% to -67.98%)	-74.34% (-77.87% to -70.15%)	-4.59% (-4.8% to -4.38%)
Uganda	39267 (31736 to 46796)	466.37 (376.92 to 555.8)	25061 (21076 to 29255)	126.34 (106.25 to 147.49)	-36.18% (-41.64% to -28.66%)	-72.91% (-75.23% to -69.72%)	-4.34% (-4.53% to -4.14%)
Ukraine	9558 (7305 to 12042)	84.03 (64.22 to 105.86)	1717 (1307 to 2277)	27.07 (20.6 to 35.88)	-82.03% (-83.97% to -79.44%)	-67.79% (-71.27% to -63.14%)	-3.28% (-3.57% to -2.99%)
United Arab Emirates	268 (209 to 337)	45.46 (35.5 to 57.13)	207 (147 to 288)	15.49 (11.01 to 21.53)	-22.59% (-34% to -8.49%)	-65.92% (-70.94% to -59.71%)	-3.72% (-3.93% to -3.51%)
United Kingdom	4804 (3472 to 6460)	43.99 (31.8 to 59.16)	1801 (1310 to 2432)	15.29 (11.12 to 20.64)	-62.5% (-64.44% to -60.33%)	-65.25% (-67.04% to -63.24%)	-3.95% (-4.19% to -3.72%)
United Republic of Tanzania	43846 (35929 to 52632)	363.1 (297.54 to 435.86)	25434 (21623 to 29516)	104.23 (88.61 to 120.95)	-41.99% (-47.59% to -35.57%)	-71.3% (-74.07% to -68.12%)	-4.06% (-4.48% to -3.65%)
United States of America	15202 (10408 to 21141)	27.19 (18.62 to 37.81)	2386 (1823 to 3145)	4.02 (3.07 to 5.29)	-84.3% (-85.68% to -82.21%)	-85.23% (-86.53% to -83.26%)	-6.77% (-7.54% to -6%)
United States Virgin Islands	17 (13 to 22)	52.82 (39.93 to 67.89)	5 (4 to 7)	37.89 (26.8 to 52.22)	-69.94% (-73.33% to -66.07%)	-28.27% (-36.38% to -19.04%)	-0.99% (-1.18% to -0.81%)
Uruguay	295 (239 to 361)	36.04 (29.17 to 44.13)	60 (44 to 83)	9.11 (6.72 to 12.56)	-79.64% (-82.64% to -76.25%)	-74.73% (-78.45% to -70.52%)	-4.76% (-4.97% to -4.55%)
Uzbekistan	9155 (7598 to 10806)	107 (88.8 to 126.3)	2877 (2153 to 3826)	28.51 (21.33 to 37.92)	-68.57% (-73.37% to -62.96%)	-73.36% (-77.42% to -68.6%)	-4.55% (-4.73% to -4.37%)
Vanuatu	59 (48 to 72)	87.08 (70.53 to 105.24)	61 (50 to 75)	52.06 (42.5 to 64.18)	2.34% (-6.37% to 11.72%)	-40.21% (-45.3% to -34.73%)	-1.9% (-2.27% to -1.53%)
Venezuela (Bolivarian Republic of)	4011 (3332 to 4767)	56.54 (46.96 to 67.2)	1561 (1255 to 1930)	23.56 (18.94 to 29.13)	-61.09% (-65.9% to -55.65%)	-58.33% (-63.48% to -52.5%)	-2.64% (-3.22% to -2.06%)
Viet Nam	16079 (12542 to 19870)	60.65 (47.3 to 74.95)	3841 (3128 to 4581)	15.51 (12.63 to 18.5)	-76.11% (-78.71% to -72.6%)	-74.43% (-77.21% to -70.66%)	-4.2% (-4.42% to -3.99%)
Yemen	6717 (5501 to 8043)	94.68 (77.55 to 113.37)	3998 (3144 to 4973)	28.99 (22.8 to 36.07)	-40.49% (-48.28% to -31.43%)	-69.38% (-73.39% to -64.72%)	-4.09% (-4.35% to -3.82%)
Zambia	25917 (21130 to 31275)	690.28 (562.78 to 832.99)	8800 (7411 to 10502)	106.39 (89.6 to 126.97)	-66.05% (-69.49% to -62.37%)	-84.59% (-86.15% to -82.92%)	-5.96% (-6.33% to -5.58%)
Zimbabwe	5957 (4830 to 7097)	123.68 (100.29 to 147.36)	5081 (4332 to 5864)	80.73 (68.82 to 93.16)	-14.7% (-21.6% to -4.19%)	-34.72% (-40.01% to -26.68%)	-1.29% (-1.64% to -0.94%)

EAPC, estimated annual percentage change.

S3 Table. Deaths of childhood meningitis in 1990 and 2021, with EAPC from 1990 and 2021.

Location	Deaths in 1990	Rate in 1990 (per 100,000)	Deaths in 2021	Rate in 2021 (per 100,000)	Changes of deaths, 1990-2021	Changes of death rates, 1990-2021	EAPC 1990-2021
Global	315176 (270536 to 372951)	18.12 (15.56 to 21.44)	112373 (80908 to 154126)	5.59 (4.02 to 7.66)	-64.35% (-72.68 to -52.42)	-69.18% (-76.38 to -58.87)	-3.4% (-3.76 to -3.04)
**5 SDI quintiles regions**							
High SDI	2565 (2456 to 2700)	1.38 (1.32 to 1.45)	354 (320 to 386)	0.21 (0.19 to 0.22)	-86.21% (-87.49 to -85.02)	-85.14% (-86.53 to -83.87)	-5.92% (-6.14 to -5.71)
High-middle SDI	14581 (12507 to 17515)	5.33 (4.57 to 6.4)	1283 (1105 to 1505)	0.56 (0.48 to 0.65)	-91.2% (-93.14 to -89.16)	-89.57% (-91.87 to -87.16)	-6.69% (-6.91 to -6.47)
Middle SDI	60495 (53179 to 67664)	10.48 (9.21 to 11.72)	10514 (8333 to 12924)	1.85 (1.47 to 2.28)	-82.62% (-85.98 to -78.4)	-82.3% (-85.72 to -78)	-4.9% (-5.21 to -4.59)
Low-middle SDI	104746 (90496 to 122640)	22.19 (19.17 to 25.98)	31552 (24000 to 41693)	5.44 (4.14 to 7.19)	-69.88% (-76.72 to -59.32)	-75.47% (-81.04 to -66.88)	-4.01% (-4.41 to -3.61)
Low SDI	132584 (109074 to 166442)	57.92 (47.65 to 72.71)	68603 (46456 to 102445)	14.91 (10.09 to 22.26)	-48.26% (-61.04 to -28.45)	-74.26% (-80.62 to -64.41)	-4.26% (-4.57 to -3.94)
**GBD regions**							
Andean Latin America	914 (792 to 1098)	6.16 (5.33 to 7.39)	118 (89 to 157)	0.65 (0.49 to 0.87)	-87.15% (-90.35 to -82.36)	-89.45% (-92.08 to -85.52)	-7.12% (-7.33 to -6.92)
Australasia	59 (55 to 64)	1.29 (1.19 to 1.39)	14 (12 to 17)	0.25 (0.21 to 0.29)	-76.01% (-80.2 to -71.22)	-80.8% (-84.15 to -76.97)	-5.73% (-6.32 to -5.15)
Caribbean	2279 (1914 to 2760)	19.97 (16.77 to 24.18)	755 (529 to 1085)	6.56 (4.6 to 9.43)	-66.86% (-76.68 to -51.86)	-67.13% (-76.87 to -52.24)	-3.05% (-3.31 to -2.8)
Central Asia	2324 (2108 to 2569)	9.3 (8.43 to 10.28)	298 (230 to 391)	1.08 (0.83 to 1.41)	-87.16% (-90.35 to -82.67)	-88.41% (-91.28 to -84.35)	-6.85% (-7.13 to -6.57)
Central Europe	902 (852 to 953)	3.06 (2.89 to 3.23)	45 (37 to 53)	0.25 (0.21 to 0.3)	-95.04% (-95.88 to -94)	-91.74% (-93.13 to -90)	-7.08% (-7.32 to -6.84)
Central Latin America	3484 (3246 to 3764)	5.41 (5.04 to 5.85)	487 (379 to 626)	0.77 (0.6 to 0.99)	-86.03% (-89.4 to -81.68)	-85.83% (-89.26 to -81.43)	-6.27% (-6.79 to -5.75)
Central Sub-Saharan Africa	12954 (9776 to 16673)	51.2 (38.64 to 65.91)	5713 (3879 to 9968)	9.74 (6.61 to 16.99)	-55.9% (-65.78 to -35.82)	-80.99% (-85.25 to -72.33)	-5.21% (-5.81 to -4.6)
East Asia	28180 (21117 to 35527)	8.54 (6.4 to 10.77)	1483 (1194 to 1791)	0.55 (0.45 to 0.67)	-94.74% (-96.06 to -93.15)	-93.51% (-95.14 to -91.55)	-8.26% (-8.69 to -7.83)

Location	Deaths in 1990	Rate in 1990 (per 100,000)	Deaths in 2021	Rate in 2021 (per 100,000)	Changes of deaths, 1990-2021	Changes of death rates, 1990-2021	EAPC 1990-2021
Eastern Europe	1846 (1754 to 1949)	3.59 (3.41 to 3.79)	203 (183 to 223)	0.57 (0.52 to 0.63)	-89.03% (-90.01 to -87.9)	-84.07% (-85.49 to -82.43)	-4.61% (-5.16 to -4.05)
Eastern Sub-Saharan Africa	57646 (44497 to 74585)	63.65 (49.13 to 82.35)	20805 (14754 to 29026)	11.66 (8.27 to 16.27)	-63.91% (-72.95 to -48.32)	-81.68% (-86.27 to -73.77)	-5.46% (-5.71 to -5.21)
High-income Asia Pacific	258 (226 to 290)	0.73 (0.64 to 0.82)	18 (16 to 20)	0.08 (0.07 to 0.09)	-93.12% (-94.02 to -91.89)	-89.21% (-90.61 to -87.27)	-6.84% (-7.15 to -6.54)
High-income North America	721 (688 to 761)	1.17 (1.12 to 1.23)	125 (112 to 139)	0.19 (0.17 to 0.21)	-82.67% (-84.5 to -80.86)	-83.71% (-85.43 to -82.01)	-5.61% (-5.93 to -5.28)
North Africa and Middle East	12842 (10661 to 15927)	9.14 (7.59 to 11.34)	2973 (2116 to 4329)	1.62 (1.15 to 2.36)	-76.85% (-82.46 to -67.37)	-82.26% (-86.56 to -74.99)	-5.18% (-5.71 to -4.65)
Oceania	307 (237 to 404)	11.45 (8.84 to 15.07)	224 (145 to 341)	4.4 (2.86 to 6.72)	-27.08% (-54.51 to 19.44)	-61.54% (-76.01 to -37)	-4.49% (-5.08 to -3.89)
South Asia	79552 (67027 to 95730)	18.36 (15.47 to 22.09)	22955 (17613 to 30148)	4.53 (3.47 to 5.95)	-71.14% (-78.42 to -61.1)	-75.34% (-81.56 to -66.75)	-3.91% (-4.28 to -3.55)
Southeast Asia	29699 (23788 to 36837)	17.39 (13.93 to 21.57)	6982 (5423 to 8748)	4.04 (3.14 to 5.07)	-76.49% (-82.33 to -67.31)	-76.75% (-82.52 to -67.67)	-4.36% (-4.66 to -4.05)
Southern Latin America	604 (566 to 646)	4.05 (3.79 to 4.33)	76 (63 to 90)	0.52 (0.44 to 0.62)	-87.47% (-89.77 to -84.77)	-87.1% (-89.47 to -84.32)	-5.88% (-6.12 to -5.64)
Southern Sub-Saharan Africa	2441 (2002 to 2928)	11.8 (9.68 to 14.15)	1267 (983 to 1611)	5.27 (4.09 to 6.69)	-48.09% (-61.67 to -26.36)	-55.37% (-67.05 to -36.69)	-2.32% (-2.73 to -1.9)
Tropical Latin America	5654 (5095 to 6216)	10.55 (9.5 to 11.59)	425 (342 to 515)	0.85 (0.68 to 1.03)	-92.48% (-94.01 to -90.82)	-91.97% (-93.6 to -90.19)	-8.19% (-8.59 to -7.8)
Western Europe	1018 (992 to 1045)	1.43 (1.4 to 1.47)	174 (155 to 194)	0.26 (0.23 to 0.29)	-82.86% (-84.78 to -80.85)	-82.13% (-84.13 to -80.04)	-5.5% (-5.69 to -5.3)
Western Sub-Saharan Africa	71492 (55274 to 92522)	81.35 (62.9 to 105.28)	47231 (28668 to 74281)	21.99 (13.35 to 34.59)	-33.94% (-51.76 to -9.94)	-72.97% (-80.26 to -63.15)	-3.97% (-4.41 to -3.53)

SDI, sociodemographic index; EAPC, estimated annual percentage change

S4 Table. Deaths of childhood meningitis in 204 countries, 1990 and 2021, with EAPC from 1990 to 2021.

Location	Deaths in 1990	Rate in 1990 (per 100,000)	Deaths in 2021	Rate in 2021 (per 100,000)	Changes of deaths, 1990-2021	Changes of rates, 1990-2021	EAPC 1990-2021
Afghanistan	2885 (2002 to 3861)	66.97 (46.47 to 89.63)	1662 (1043 to 2719)	11.71 (7.35 to 19.14)	-42.38% (-62.27% to -3.03%)	-82.52% (-88.55% to -70.58%)	-5.57% (-6.03% to -5.11%)
Albania	71 (54 to 91)	6.36 (4.83 to 8.19)	2 (2 to 4)	0.55 (0.34 to 0.93)	-96.54% (-98.05% to -93.63%)	-91.28% (-95.08% to -83.96%)	-8.27% (-8.49% to -8.05%)
Algeria	541 (373 to 790)	5.05 (3.48 to 7.36)	71 (44 to 111)	0.53 (0.33 to 0.84)	-86.89% (-91.22% to -80.31%)	-89.43% (-92.92% to -84.13%)	-5.88% (-6.42% to -5.33%)
American Samoa	1 (0 to 1)	3.29 (2.52 to 4.24)	0 (0 to 0)	1.44 (1.06 to 1.87)	-67.44% (-77.62% to -53.66%)	-56.33% (-69.98% to -37.84%)	-2.78% (-3.05% to -2.5%)
Andorra	0 (0 to 0)	0.15 (0.1 to 0.23)	0 (0 to 0)	0.01 (0.01 to 0.01)	-92.73% (-95.86% to -87.28%)	-93.21% (-96.13% to -88.11%)	-8.53% (-8.97% to -8.08%)
Angola	3581 (2624 to 4541)	75.95 (55.65 to 96.31)	1752 (1201 to 2473)	11.49 (7.88 to 16.22)	-51.08% (-66.9% to -23.97%)	-84.87% (-89.76% to -76.49%)	-6.18% (-6.65% to -5.7%)
Antigua and Barbuda	1 (1 to 1)	3.5 (2.84 to 4.23)	0 (0 to 0)	0.83 (0.68 to 0.97)	-78.06% (-83.25% to -72.38%)	-76.39% (-81.97% to -70.27%)	-5.24% (-5.67% to -4.81%)
Argentina	438 (405 to 475)	4.32 (4 to 4.69)	60 (50 to 72)	0.59 (0.49 to 0.71)	-86.21% (-89.02% to -82.92%)	-86.27% (-89.07% to -83%)	-5.67% (-5.93% to -5.41%)
Armenia	22 (19 to 26)	2.13 (1.83 to 2.45)	1 (1 to 1)	0.11 (0.09 to 0.14)	-97.06% (-97.71% to -96.18%)	-94.82% (-95.98% to -93.28%)	-8.61% (-9.08% to -8.14%)
Australia	48 (44 to 52)	1.27 (1.16 to 1.37)	10 (8 to 12)	0.2 (0.17 to 0.24)	-79.83% (-83.88% to -75.21%)	-83.92% (-87.15% to -80.24%)	-5.68% (-6.06% to -5.3%)
Austria	19 (17 to 20)	1.39 (1.26 to 1.52)	3 (2 to 3)	0.2 (0.16 to 0.23)	-86.3% (-88.59% to -83.49%)	-85.76% (-88.14% to -82.83%)	-5.62% (-5.93% to -5.3%)
Azerbaijan	151 (122 to 180)	6.22 (5.05 to 7.42)	20 (15 to 30)	0.86 (0.62 to 1.29)	-86.55% (-90.84% to -79.7%)	-86.18% (-90.59% to -79.13%)	-6.22% (-6.78% to -5.67%)
Bahamas	3 (3 to 4)	4.24 (3.49 to 5.18)	0 (0 to 1)	0.49 (0.37 to 0.69)	-88.33% (-91.79% to -83.27%)	-88.4% (-91.84% to -83.38%)	-7.25% (-7.61% to -6.89%)
Bahrain	3 (2 to 3)	1.61 (1.34 to 1.97)	1 (0 to 1)	0.21 (0.16 to 0.27)	-76.75% (-82.23% to -68.45%)	-87.21% (-90.22% to -82.64%)	-6.17% (-6.49% to -5.86%)
Bangladesh	5575 (2110 to 8455)	11.4 (4.31 to 17.29)	1038 (528 to 1596)	2.27 (1.15 to 3.49)	-81.39% (-87.32% to -63.95%)	-80.11% (-86.45% to -61.47%)	-4.21% (-4.83% to -3.58%)
Barbados	2 (2 to 3)	3.92 (3.28 to 4.59)	0 (0 to 1)	0.86 (0.6 to 1.2)	-83.46% (-88.29% to -76.82%)	-78.09% (-84.5% to -69.31%)	-5.29% (-5.79% to -4.79%)
Belarus	80 (68 to 94)	3.31 (2.82 to 3.91)	9 (7 to 12)	0.59 (0.46 to 0.77)	-88.23% (-91.25% to -84.24%)	-82.07% (-86.68% to -76%)	-4.37% (-4.77% to -3.97%)

Location	Deaths in 1990	Rate in 1990 (per 100,000)	Deaths in 2021	Rate in 2021 (per 100,000)	Changes of deaths, 1990-2021	Changes of rates, 1990-2021	EAPC 1990-2021
Belgium	21 (19 to 22)	1.15 (1.06 to 1.23)	5 (4 to 6)	0.24 (0.2 to 0.29)	-77.47% (-81.74% to -72.48%)	-78.72% (-82.75% to -74%)	-5.25% (-5.45% to -5.06%)
Belize	6 (5 to 7)	7.25 (6.4 to 8.19)	1 (1 to 1)	0.6 (0.48 to 0.73)	-87.61% (-90.35% to -84.33%)	-91.76% (-93.59% to -89.58%)	-7.95% (-8.33% to -7.56%)
Benin	1627 (1234 to 2137)	67.19 (50.96 to 88.25)	983 (523 to 1757)	16.17 (8.61 to 28.9)	-39.56% (-64.33% to 4.89%)	-75.93% (-85.79% to -58.22%)	-4.55% (-5.01% to -4.08%)
Bermuda	0 (0 to 0)	2.19 (1.81 to 2.58)	0 (0 to 0)	0.29 (0.21 to 0.36)	-90.67% (-93.12% to -87.91%)	-86.81% (-90.28% to -82.92%)	-6.71% (-7.13% to -6.29%)
Bhutan	59 (21 to 91)	22.66 (8.05 to 34.56)	6 (4 to 9)	3.38 (2.29 to 4.91)	-89.34% (-94.01% to -68.8%)	-85.07% (-91.62% to -56.3%)	-6.4% (-6.81% to -6%)
Bolivia (Plurinational State of)	350 (269 to 504)	13.03 (10.03 to 18.77)	45 (33 to 66)	1.3 (0.94 to 1.91)	-87.06% (-91.27% to -79.63%)	-90.03% (-93.28% to -84.31%)	-7.15% (-7.41% to -6.89%)
Bosnia and Herzegovina	11 (9 to 14)	1.03 (0.81 to 1.28)	1 (1 to 2)	0.25 (0.18 to 0.34)	-89.35% (-92.7% to -84.59%)	-76.21% (-83.7% to -65.57%)	-5.26% (-5.94% to -4.57%)
Botswana	61 (46 to 81)	10.4 (7.81 to 13.78)	36 (25 to 51)	5.1 (3.53 to 7.31)	-42.04% (-62.52% to -9.61%)	-50.99% (-68.31% to -23.57%)	-1.76% (-2.36% to -1.15%)
Brazil	5533 (4975 to 6099)	10.65 (9.58 to 11.74)	398 (321 to 479)	0.83 (0.67 to 0.99)	-92.81% (-94.29% to -91.24%)	-92.25% (-93.84% to -90.56%)	-8.29% (-8.7% to -7.89%)
Brunei Darussalam	1 (1 to 1)	0.99 (0.78 to 1.33)	0 (0 to 1)	0.44 (0.33 to 0.57)	-53.91% (-70.3% to -30.61%)	-55.87% (-71.56% to -33.56%)	-2.5% (-2.86% to -2.14%)
Bulgaria	52 (47 to 59)	3.02 (2.69 to 3.37)	5 (4 to 6)	0.48 (0.39 to 0.57)	-91.05% (-92.92% to -89.1%)	-84.07% (-87.41% to -80.61%)	-5.83% (-6.23% to -5.44%)
Burkina Faso	4023 (3106 to 5200)	85.25 (65.82 to 110.2)	2402 (1368 to 4113)	23.16 (13.19 to 39.65)	-40.29% (-62.43% to -0.9%)	-72.83% (-82.91% to -54.91%)	-4.22% (-5.01% to -3.43%)
Burundi	1455 (1079 to 1902)	55.53 (41.17 to 72.56)	524 (287 to 990)	8.94 (4.91 to 16.91)	-64.03% (-77.45% to -37%)	-83.89% (-89.9% to -71.79%)	-5.56% (-6.2% to -4.91%)
Cabo Verde	23 (15 to 31)	14.36 (9.53 to 20.01)	2 (2 to 3)	1.46 (1.12 to 1.91)	-90.77% (-94.22% to -83.5%)	-89.86% (-93.65% to -81.87%)	-8.49% (-9.32% to -7.64%)
Cambodia	1032 (748 to 1421)	22.15 (16.06 to 30.48)	164 (113 to 253)	3.21 (2.2 to 4.95)	-84.08% (-90.01% to -74.79%)	-85.5% (-90.9% to -77.04%)	-6.34% (-6.81% to -5.86%)
Cameroon	2349 (1777 to 3139)	48.12 (36.4 to 64.31)	1625 (941 to 2481)	12.07 (6.98 to 18.42)	-30.83% (-54.33% to 5.42%)	-74.92% (-83.44% to -61.78%)	-4.21% (-4.81% to -3.6%)
Canada	44 (41 to 47)	0.76 (0.71 to 0.82)	9 (8 to 10)	0.14 (0.12 to 0.17)	-79.63% (-83.14% to -75.61%)	-81.01% (-84.29% to -77.27%)	-4.51% (-4.78% to -4.23%)
Central African Republic	832 (669 to 1137)	68.02 (54.72 to 93.01)	501 (322 to 847)	21.94 (14.1 to 37.07)	-39.76% (-60.13% to -8.52%)	-67.75% (-78.66% to -51.03%)	-3.21% (-3.67% to -2.75%)

Location	Deaths in 1990	Rate in 1990 (per 100,000)	Deaths in 2021	Rate in 2021 (per 100,000)	Changes of deaths, 1990-2021	Changes of rates, 1990-2021	EAPC 1990-2021
Chad	1723 (1280 to 2336)	58.89 (43.75 to 79.83)	2792 (1855 to 4030)	30.98 (20.58 to 44.7)	62.02% (11.78% to 134.96%)	-47.41% (-63.71% to -23.73%)	-2.25% (-2.65% to -1.85%)
Chile	141 (132 to 150)	3.55 (3.32 to 3.77)	12 (11 to 14)	0.34 (0.3 to 0.39)	-91.17% (-92.5% to -89.63%)	-90.4% (-91.84% to -88.72%)	-6.85% (-7.11% to -6.58%)
China	27816 (20776 to 35196)	8.74 (6.53 to 11.05)	1424 (1151 to 1721)	0.55 (0.44 to 0.66)	-94.88% (-96.23% to -93.34%)	-93.72% (-95.38% to -91.84%)	-8.37% (-8.8% to -7.93%)
Colombia	816 (715 to 936)	7 (6.13 to 8.02)	84 (62 to 112)	0.79 (0.59 to 1.06)	-89.75% (-92.46% to -86.25%)	-88.74% (-91.71% to -84.89%)	-7.14% (-7.5% to -6.78%)
Comoros	88 (64 to 115)	41.48 (30.13 to 54.27)	29 (21 to 38)	11.93 (8.54 to 15.88)	-67.52% (-77.91% to -52.23%)	-71.24% (-80.44% to -57.69%)	-3.98% (-4.31% to -3.66%)
Congo	282 (215 to 372)	26.74 (20.45 to 35.31)	112 (76 to 159)	5.83 (3.96 to 8.24)	-60.05% (-72.41% to -41.47%)	-78.2% (-84.94% to -68.06%)	-5.24% (-6.04% to -4.43%)
Cook Islands	0 (0 to 0)	1.01 (0.76 to 1.31)	0 (0 to 0)	0.25 (0.18 to 0.34)	-86.06% (-90.43% to -79.21%)	-75.73% (-83.33% to -63.8%)	-6.92% (-7.77% to -6.05%)
Costa Rica	31 (29 to 34)	2.79 (2.57 to 3.06)	5 (5 to 6)	0.53 (0.45 to 0.62)	-82.77% (-85.74% to -79.43%)	-80.96% (-84.24% to -77.27%)	-5.18% (-5.57% to -4.79%)
Coted'Ivoire	2319 (1683 to 3134)	40.66 (29.51 to 54.96)	1342 (770 to 2131)	11.59 (6.65 to 18.42)	-42.15% (-62.46% to -7.3%)	-71.49% (-81.5% to -54.31%)	-3.56% (-4.16% to -2.95%)
Croatia	17 (16 to 18)	1.71 (1.58 to 1.86)	1 (1 to 2)	0.24 (0.18 to 0.29)	-91.63% (-93.54% to -89.53%)	-86.17% (-89.32% to -82.69%)	-5.46% (-5.95% to -4.96%)
Cuba	225 (213 to 236)	8.98 (8.51 to 9.44)	13 (10 to 15)	0.71 (0.59 to 0.84)	-94.39% (-95.34% to -93.24%)	-92.09% (-93.43% to -90.47%)	-6.95% (-7.77% to -6.12%)
Cyprus	3 (2 to 4)	1.53 (1.15 to 2.1)	0 (0 to 0)	0.13 (0.1 to 0.18)	-90.43% (-93.49% to -85.21%)	-91.34% (-94.11% to -86.62%)	-7.63% (-7.87% to -7.38%)
Czechia	32 (29 to 35)	1.44 (1.3 to 1.57)	3 (2 to 4)	0.18 (0.14 to 0.21)	-90.36% (-92.16% to -88.41%)	-87.62% (-89.93% to -85.12%)	-6.17% (-6.6% to -5.73%)
Democratic People's Republic of [illegible]	282 (194 to 386)	4.75 (3.26 to 6.5)	51 (34 to 81)	1.07 (0.7 to 1.69)	-81.91% (-88.44% to -71.77%)	-77.45% (-85.6% to -64.82%)	-4.43% (-4.9% to -3.96%)
Democratic Republic of the Congo	8080 (5521 to 11347)	45.64 (31.18 to 64.09)	3269 (2112 to 6133)	8.6 (5.56 to 16.14)	-59.55% (-71.27% to -39.22%)	-81.15% (-86.62% to -71.68%)	-5.12% (-5.82% to -4.42%)
Denmark	27 (25 to 29)	3.07 (2.83 to 3.32)	5 (4 to 6)	0.49 (0.41 to 0.58)	-82.69% (-85.89% to -79.24%)	-83.97% (-86.94% to -80.78%)	-6.43% (-6.94% to -5.92%)
Djibouti	56 (41 to 74)	32.34 (23.37 to 42.77)	33 (23 to 47)	8.1 (5.67 to 11.35)	-40.6% (-61.26% to -7.99%)	-74.97% (-83.68% to -61.23%)	-4.49% (-5.19% to -3.79%)
Dominica	1 (1 to 1)	3.88 (2.94 to 5.04)	0 (0 to 0)	2.25 (1.52 to 3.39)	-68.01% (-81.42% to -45.83%)	-41.99% (-66.31% to -1.75%)	-1.86% (-2.48% to -1.23%)

Location	Deaths in 1990	Rate in 1990 (per 100,000)	Deaths in 2021	Rate in 2021 (per 100,000)	Changes of deaths, 1990-2021	Changes of rates, 1990-2021	EAPC 1990-2021
Dominican Republic	502 (396 to 616)	18.64 (14.7 to 22.85)	78 (56 to 115)	2.67 (1.89 to 3.91)	-84.4% (-89.42% to -76.21%)	-85.69% (-90.29% to -78.17%)	-5.69% (-6.06% to -5.32%)
Ecuador	150 (138 to 163)	3.88 (3.57 to 4.23)	27 (21 to 36)	0.54 (0.41 to 0.71)	-81.78% (-86.19% to -76.63%)	-86.11% (-89.47% to -82.19%)	-6.59% (-7.23% to -5.94%)
Egypt	1390 (1133 to 1703)	6.27 (5.11 to 7.68)	235 (177 to 308)	0.64 (0.48 to 0.84)	-83.1% (-87.98% to -76.23%)	-89.83% (-92.76% to -85.69%)	-6.49% (-6.87% to -6.1%)
El Salvador	143 (115 to 174)	6.64 (5.34 to 8.04)	9 (7 to 13)	0.51 (0.37 to 0.71)	-93.56% (-95.58% to -90.23%)	-92.36% (-94.76% to -88.4%)	-8.45% (-8.92% to -7.98%)
Equatorial Guinea	84 (64 to 119)	42.53 (32.31 to 60.26)	41 (22 to 69)	7.02 (3.79 to 11.74)	-50.97% (-73.74% to -12.78%)	-83.5% (-91.16% to -70.64%)	-6.45% (-6.79% to -6.11%)
Eritrea	706 (512 to 960)	44.37 (32.17 to 60.32)	278 (170 to 441)	11 (6.74 to 17.46)	-60.68% (-76.66% to -30.31%)	-75.2% (-85.28% to -56.06%)	-4.14% (-4.56% to -3.71%)
Estonia	11 (10 to 12)	3.19 (2.87 to 3.52)	1 (1 to 1)	0.32 (0.27 to 0.39)	-93.8% (-94.97% to -92.37%)	-89.98% (-91.87% to -87.68%)	-6.62% (-6.88% to -6.36%)
Eswatini	58 (42 to 76)	15.08 (10.97 to 19.8)	26 (18 to 36)	6.19 (4.37 to 8.6)	-56.1% (-70.71% to -33.38%)	-58.96% (-72.62% to -37.72%)	-2.35% (-3.01% to -1.7%)
Ethiopia	19890 (14871 to 26032)	81.64 (61.04 to 106.85)	5542 (4098 to 7084)	12.5 (9.24 to 15.97)	-72.14% (-79.68% to -59.94%)	-84.69% (-88.84% to -77.99%)	-6.44% (-6.68% to -6.21%)
Fiji	17 (12 to 24)	6 (4.44 to 8.41)	6 (4 to 9)	2.32 (1.55 to 3.39)	-62.48% (-77.97% to -42.49%)	-61.25% (-77.25% to -40.6%)	-3.4% (-3.93% to -2.87%)
Finland	11 (10 to 12)	1.17 (1.07 to 1.28)	2 (2 to 3)	0.26 (0.22 to 0.3)	-80.51% (-83.8% to -76.67%)	-77.8% (-81.55% to -73.43%)	-5.09% (-5.32% to -4.86%)
France	138 (129 to 147)	1.17 (1.1 to 1.25)	33 (28 to 39)	0.29 (0.24 to 0.33)	-75.78% (-80.05% to -71.56%)	-75.56% (-79.87% to -71.3%)	-4.5% (-4.88% to -4.13%)
Gabon	96 (76 to 121)	23.62 (18.71 to 29.67)	38 (24 to 59)	6.02 (3.83 to 9.21)	-60.02% (-74.09% to -38.42%)	-74.51% (-83.48% to -60.74%)	-3.29% (-3.84% to -2.73%)
Gambia	192 (141 to 253)	41.53 (30.58 to 54.82)	86 (56 to 140)	8.62 (5.65 to 14.05)	-55.28% (-69.93% to -30.78%)	-79.24% (-86.04% to -67.86%)	-5.14% (-5.64% to -4.63%)
Georgia	49 (42 to 56)	3.55 (3.1 to 4.13)	1 (1 to 2)	0.2 (0.15 to 0.25)	-97.04% (-97.72% to -96.1%)	-94.5% (-95.76% to -92.75%)	-8.55% (-9.09% to -8.01%)
Germany	150 (141 to 160)	1.16 (1.09 to 1.23)	21 (19 to 24)	0.18 (0.15 to 0.2)	-85.9% (-87.8% to -83.92%)	-84.74% (-86.8% to -82.6%)	-5.28% (-5.61% to -4.96%)
Ghana	2757 (1914 to 3917)	41.05 (28.5 to 58.32)	1064 (664 to 1692)	8.26 (5.15 to 13.14)	-61.42% (-73.11% to -40.9%)	-79.89% (-85.98% to -69.19%)	-4.89% (-5.29% to -4.48%)
Greece	13 (12 to 14)	0.66 (0.61 to 0.71)	3 (3 to 4)	0.22 (0.19 to 0.27)	-76.39% (-80.64% to -71.75%)	-65.75% (-71.91% to -59.02%)	-3.44% (-3.8% to -3.07%)

Location	Deaths in 1990	Rate in 1990 (per 100,000)	Deaths in 2021	Rate in 2021 (per 100,000)	Changes of deaths, 1990-2021	Changes of rates, 1990-2021	EAPC 1990-2021
Greenland	2 (1 to 3)	14.53 (10.35 to 20.24)	0 (0 to 0)	2.06 (1.32 to 2.91)	-88.3% (-92.41% to -81.56%)	-85.84% (-90.81% to -77.69%)	-5.93% (-6.47% to -5.39%)
Grenada	1 (1 to 2)	4.39 (3.63 to 5.36)	0 (0 to 0)	0.37 (0.3 to 0.46)	-94.47% (-95.59% to -93.11%)	-91.54% (-93.25% to -89.46%)	-7.1% (-7.68% to -6.52%)
Guam	1 (1 to 1)	2.41 (1.84 to 2.92)	0 (0 to 0)	1.06 (0.8 to 1.36)	-61.47% (-73.69% to -44.12%)	-56.07% (-70% to -36.29%)	-1.74% (-2.37% to -1.11%)
Guatemala	249 (225 to 278)	6.13 (5.55 to 6.84)	58 (47 to 74)	1.18 (0.94 to 1.51)	-76.59% (-81.59% to -70.14%)	-80.73% (-84.85% to -75.42%)	-5.6% (-5.94% to -5.26%)
Guinea	2467 (1855 to 3213)	89.64 (67.41 to 116.75)	1479 (855 to 2536)	24.46 (14.15 to 41.94)	-40.05% (-62.36% to 2.03%)	-72.71% (-82.87% to -53.56%)	-3.68% (-4% to -3.35%)
Guinea-Bissau	384 (274 to 519)	79.65 (56.85 to 107.65)	101 (66 to 173)	11.21 (7.3 to 19.22)	-73.79% (-82.03% to -57.43%)	-85.92% (-90.35% to -77.13%)	-6.1% (-6.9% to -5.29%)
Guyana	18 (15 to 22)	6.19 (4.94 to 7.58)	2 (1 to 3)	0.87 (0.64 to 1.19)	-89.82% (-92.95% to -85.52%)	-85.98% (-90.29% to -80.05%)	-5.96% (-6.31% to -5.6%)
Haiti	1345 (1010 to 1774)	49.59 (37.22 to 65.38)	625 (414 to 923)	14.35 (9.52 to 21.2)	-53.57% (-69.05% to -29.38%)	-71.06% (-80.71% to -55.98%)	-3.5% (-3.69% to -3.31%)
Honduras	124 (98 to 159)	5.63 (4.44 to 7.2)	39 (15 to 67)	1.2 (0.46 to 2.04)	-68.37% (-89.23% to -43.48%)	-78.68% (-92.74% to -61.89%)	-4.24% (-4.74% to -3.73%)
Hungary	52 (47 to 58)	2.44 (2.2 to 2.71)	5 (4 to 7)	0.38 (0.3 to 0.47)	-89.74% (-91.76% to -87.23%)	-84.25% (-87.36% to -80.4%)	-5.25% (-5.7% to -4.8%)
Iceland	1 (1 to 1)	1.71 (1.48 to 1.94)	0 (0 to 0)	0.36 (0.29 to 0.44)	-77.6% (-82.1% to -71.63%)	-78.96% (-83.19% to -73.35%)	-5.31% (-5.5% to -5.12%)
India	52227 (41600 to 68445)	16 (12.74 to 20.96)	11872 (8849 to 15771)	3.24 (2.42 to 4.3)	-77.27% (-83.69% to -67.93%)	-79.74% (-85.47% to -71.42%)	-4.61% (-5.06% to -4.15%)
Indonesia	15053 (10737 to 18833)	22.22 (15.85 to 27.8)	3898 (2901 to 5098)	5.79 (4.31 to 7.58)	-74.11% (-82.1% to -60.31%)	-73.93% (-81.98% to -60.05%)	-4.13% (-4.37% to -3.89%)
Iran (Islamic Republic of)	1176 (942 to 1605)	4.63 (3.71 to 6.32)	56 (44 to 70)	0.28 (0.22 to 0.35)	-95.26% (-96.53% to -93.86%)	-94.04% (-95.64% to -92.28%)	-6.5% (-7.58% to -5.4%)
Iraq	697 (540 to 880)	8.47 (6.56 to 10.69)	171 (124 to 236)	1.27 (0.92 to 1.75)	-75.49% (-82.94% to -65.27%)	-85% (-89.56% to -78.75%)	-5.49% (-6.1% to -4.88%)
Ireland	12 (11 to 13)	1.19 (1.08 to 1.29)	2 (2 to 2)	0.18 (0.15 to 0.22)	-84.23% (-86.94% to -81%)	-84.46% (-87.13% to -81.28%)	-6.01% (-6.75% to -5.28%)
Israel	27 (24 to 29)	1.74 (1.59 to 1.87)	6 (5 to 7)	0.22 (0.19 to 0.26)	-77.86% (-81.44% to -73.41%)	-87.08% (-89.17% to -84.49%)	-5.85% (-6.12% to -5.58%)
Italy	53 (50 to 55)	0.57 (0.54 to 0.6)	11 (9 to 12)	0.14 (0.12 to 0.16)	-79.85% (-83.12% to -76.4%)	-75.53% (-79.5% to -71.34%)	-4.52% (-4.79% to -4.25%)

Location	Deaths in 1990	Rate in 1990 (per 100,000)	Deaths in 2021	Rate in 2021 (per 100,000)	Changes of deaths, 1990-2021	Changes of rates, 1990-2021	EAPC 1990-2021
Jamaica	42 (37 to 47)	4.99 (4.43 to 5.69)	4 (3 to 5)	0.61 (0.46 to 0.8)	-91.47% (-93.76% to -88.76%)	-87.8% (-91.08% to -83.93%)	-6.85% (-7.36% to -6.33%)
Japan	97 (93 to 100)	0.42 (0.4 to 0.43)	13 (11 to 14)	0.08 (0.07 to 0.09)	-86.71% (-88.35% to -85.09%)	-80.13% (-82.58% to -77.7%)	-5.03% (-5.32% to -4.73%)
Jordan	50 (38 to 69)	3.09 (2.35 to 4.2)	22 (18 to 28)	0.61 (0.49 to 0.76)	-56.19% (-70.18% to -39.63%)	-80.3% (-86.59% to -72.86%)	-5.57% (-5.83% to -5.31%)
Kazakhstan	290 (253 to 331)	5.58 (4.88 to 6.37)	32 (25 to 41)	0.6 (0.47 to 0.76)	-88.84% (-91.39% to -85.65%)	-89.31% (-91.76% to -86.26%)	-6.86% (-7.56% to -6.16%)
Kenya	3007 (2355 to 3849)	26.92 (21.09 to 34.46)	962 (756 to 1322)	5.15 (4.05 to 7.09)	-68.01% (-76.3% to -53.15%)	-80.85% (-85.82% to -71.96%)	-4.98% (-5.43% to -4.53%)
Kiribati	14 (10 to 18)	46.12 (33.85 to 60.3)	5 (3 to 7)	12.07 (8.23 to 17.17)	-62.78% (-74.06% to -46.54%)	-73.84% (-81.77% to -62.42%)	-4.26% (-4.51% to -4.01%)
Kuwait	8 (7 to 9)	1.4 (1.25 to 1.6)	3 (2 to 3)	0.31 (0.26 to 0.38)	-65.73% (-72.13% to -57.98%)	-77.53% (-81.73% to -72.45%)	-3.18% (-3.91% to -2.45%)
Kyrgyzstan	182 (160 to 204)	10.83 (9.52 to 12.17)	11 (10 to 14)	0.5 (0.42 to 0.6)	-93.78% (-94.88% to -92.45%)	-95.41% (-96.23% to -94.43%)	-9.48% (-10.04% to -8.92%)
Lao People's Democratic Republic	539 (365 to 804)	29.26 (19.81 to 43.6)	119 (79 to 184)	5.2 (3.42 to 8)	-77.86% (-86.39% to -60.42%)	-82.23% (-89.08% to -68.23%)	-5.81% (-6.26% to -5.37%)
Latvia	21 (19 to 22)	3.68 (3.41 to 3.95)	1 (1 to 1)	0.42 (0.35 to 0.5)	-94.1% (-95.08% to -92.86%)	-88.71% (-90.57% to -86.33%)	-6.23% (-6.61% to -5.86%)
Lebanon	26 (18 to 38)	2.46 (1.75 to 3.59)	4 (3 to 5)	0.29 (0.2 to 0.41)	-85.72% (-91.45% to -76.36%)	-88.32% (-93% to -80.65%)	-6.72% (-7.1% to -6.34%)
Lesotho	81 (60 to 112)	11.86 (8.75 to 16.42)	47 (33 to 66)	7.47 (5.23 to 10.41)	-41.82% (-62.71% to -9.32%)	-37.02% (-59.62% to -1.82%)	-0.99% (-1.46% to -0.52%)
Liberia	1189 (830 to 1704)	105.17 (73.41 to 150.8)	228 (128 to 529)	10.45 (5.84 to 24.22)	-80.79% (-88.56% to -65.53%)	-90.07% (-94.09% to -82.18%)	-7.2% (-7.5% to -6.9%)
Libya	47 (31 to 71)	2.58 (1.71 to 3.92)	11 (7 to 16)	0.73 (0.46 to 1.08)	-76.74% (-85.87% to -61.97%)	-71.76% (-82.84% to -53.83%)	-3.41% (-3.6% to -3.23%)
Lithuania	23 (21 to 25)	2.79 (2.56 to 3.04)	2 (2 to 2)	0.46 (0.38 to 0.54)	-91.95% (-93.31% to -90.29%)	-83.61% (-86.36% to -80.22%)	-5.28% (-5.78% to -4.78%)
Luxembourg	1 (1 to 1)	1.15 (1.02 to 1.31)	0 (0 to 0)	0.19 (0.15 to 0.23)	-75.18% (-80.64% to -68.5%)	-83.8% (-87.37% to -79.44%)	-6.24% (-6.97% to -5.51%)
Madagascar	2445 (1841 to 3106)	44.82 (33.74 to 56.94)	1231 (852 to 1744)	10.49 (7.26 to 14.86)	-49.66% (-68.11% to -21.74%)	-76.59% (-85.17% to -63.61%)	-4.45% (-4.85% to -4.05%)
Malawi	5052 (3478 to 7071)	111.05 (76.44 to 155.41)	1105 (651 to 1653)	13.6 (8.02 to 20.35)	-78.13% (-86.07% to -62.78%)	-87.75% (-92.2% to -79.16%)	-6.86% (-7.14% to -6.57%)

**Location**	**Deaths in 1990**	**Rate in 1990 (per 100,000)**	**Deaths in 2021**	**Rate in 2021 (per 100,000)**	**Changes of deaths, 1990-2021**	**Changes of rates, 1990-2021**	**EAPC 1990-2021**
Malaysia	208 (152 to 270)	3.16 (2.31 to 4.11)	55 (44 to 70)	0.72 (0.58 to 0.92)	-73.56% (-81.12% to -61.48%)	-77.18% (-83.7% to -66.75%)	-3.77% (-4.39% to -3.15%)
Maldives	5 (4 to 7)	5.02 (3.79 to 6.57)	1 (0 to 1)	0.57 (0.41 to 0.78)	-89.27% (-93.02% to -83.32%)	-88.75% (-92.68% to -82.5%)	-6.14% (-6.56% to -5.72%)
Mali	3828 (2730 to 5177)	92.68 (66.09 to 125.37)	1791 (1283 to 2469)	15.48 (11.08 to 21.33)	-53.2% (-66.28% to -31.65%)	-83.3% (-87.97% to -75.61%)	-6% (-6.68% to -5.31%)
Malta	1 (1 to 1)	1.18 (1.01 to 1.36)	0 (0 to 0)	0.38 (0.3 to 0.47)	-76.4% (-82.19% to -69.96%)	-67.74% (-75.65% to -58.93%)	-4.39% (-5.64% to -3.13%)
Marshall Islands	1 (1 to 2)	4.56 (3.32 to 7.19)	0 (0 to 1)	1.9 (1.24 to 2.99)	-66.87% (-78.36% to -49.38%)	-58.36% (-72.8% to -36.38%)	-3.06% (-3.57% to -2.54%)
Mauritania	268 (198 to 360)	28.94 (21.41 to 38.95)	113 (81 to 164)	6.07 (4.38 to 8.83)	-57.94% (-71.26% to -39.29%)	-79.02% (-85.67% to -69.71%)	-5.04% (-5.83% to -4.24%)
Mauritius	6 (5 to 6)	1.77 (1.62 to 1.96)	2 (2 to 2)	0.89 (0.73 to 1.06)	-68.29% (-74.44% to -61.78%)	-49.53% (-59.32% to -39.18%)	3.31% (1.74% to 4.91%)
Mexico	1355 (1230 to 1522)	4.06 (3.68 to 4.56)	115 (90 to 148)	0.36 (0.28 to 0.46)	-91.51% (-93.61% to -88.94%)	-91.15% (-93.34% to -88.47%)	-7.85% (-8.52% to -7.17%)
Micronesia (Federated States of)	3 (2 to 4)	6.74 (4.95 to 9.18)	0 (0 to 1)	1.31 (0.93 to 1.96)	-87.02% (-90.92% to -80.67%)	-80.51% (-86.37% to -71%)	-5.35% (-5.54% to -5.16%)
Monaco	0 (0 to 0)	0.82 (0.55 to 1.2)	0 (0 to 0)	0.3 (0.22 to 0.4)	-48.56% (-68.33% to -13.07%)	-63.58% (-77.58% to -38.46%)	-5.09% (-5.98% to -4.18%)
Mongolia	204 (101 to 315)	22.68 (11.19 to 35.03)	13 (9 to 19)	1.22 (0.84 to 1.71)	-93.52% (-96.36% to -84.86%)	-94.64% (-96.99% to -87.46%)	-9.35% (-9.63% to -9.07%)
Montenegro	1 (1 to 2)	0.84 (0.64 to 1.1)	0 (0 to 0)	0.1 (0.07 to 0.15)	-91.46% (-94.53% to -86.39%)	-87.61% (-92.07% to -80.26%)	-6.62% (-7.15% to -6.09%)
Morocco	719 (498 to 1063)	7.35 (5.09 to 10.86)	60 (38 to 100)	0.61 (0.39 to 1.02)	-91.68% (-94.49% to -86.69%)	-91.69% (-94.49% to -86.7%)	-7.65% (-8.22% to -7.09%)
Mozambique	3942 (2994 to 5168)	63.54 (48.25 to 83.3)	1080 (731 to 1747)	7.57 (5.12 to 12.25)	-72.61% (-81.94% to -49.29%)	-88.09% (-92.15% to -77.95%)	-6.74% (-7.36% to -6.12%)
Myanmar	6287 (4449 to 8737)	42.55 (30.11 to 59.13)	1081 (720 to 1579)	6.92 (4.61 to 10.11)	-82.8% (-88.81% to -71.96%)	-83.73% (-89.41% to -73.47%)	-5.56% (-6.15% to -4.96%)
Namibia	65 (47 to 86)	10.75 (7.8 to 14.31)	35 (25 to 51)	4.26 (2.98 to 6.13)	-45.53% (-62.92% to -14.67%)	-60.35% (-73.02% to -37.9%)	-2.99% (-3.91% to -2.06%)
Nauru	0 (0 to 0)	7.29 (5.22 to 9.66)	0 (0 to 0)	3.65 (2.5 to 5.03)	-52.85% (-69.14% to -27.95%)	-49.98% (-67.26% to -23.56%)	-2.57% (-3.51% to -1.61%)
Nepal	861 (496 to 1829)	10.22 (5.88 to 21.7)	207 (138 to 307)	2.25 (1.5 to 3.33)	-75.92% (-90.79% to -49.23%)	-78.01% (-91.59% to -53.64%)	-3.77% (-4.19% to -3.35%)

Location	Deaths in 1990	Rate in 1990 (per 100,000)	Deaths in 2021	Rate in 2021 (per 100,000)	Changes of deaths, 1990-2021	Changes of rates, 1990-2021	EAPC 1990-2021
Netherlands	56 (52 to 59)	2.04 (1.89 to 2.18)	12 (11 to 14)	0.46 (0.4 to 0.53)	-77.69% (-81.38% to -74.02%)	-77.33% (-81.08% to -73.6%)	-5.68% (-6.22% to -5.14%)
New Zealand	11 (10 to 12)	1.38 (1.26 to 1.5)	4 (4 to 5)	0.46 (0.4 to 0.52)	-59.39% (-65.24% to -53.34%)	-66.9% (-71.67% to -61.97%)	-5.57% (-6.81% to -4.31%)
Nicaragua	222 (183 to 273)	12.16 (10.06 to 14.99)	17 (12 to 25)	0.86 (0.58 to 1.28)	-92.32% (-94.67% to -88.09%)	-92.94% (-95.09% to -89.05%)	-8.54% (-8.76% to -8.31%)
Niger	8269 (5884 to 11563)	203.52 (144.82 to 284.6)	3592 (2266 to 6015)	28.14 (17.75 to 47.13)	-56.56% (-71.28% to -22.51%)	-86.17% (-90.86% to -75.33%)	-6.97% (-7.46% to -6.48%)
Nigeria	35352 (26376 to 47005)	90.36 (67.42 to 120.14)	28327 (16052 to 45826)	27.88 (15.8 to 45.11)	-19.87% (-43.36% to 9.41%)	-69.14% (-78.19% to -57.86%)	-3.3% (-3.76% to -2.83%)
Niue	0 (0 to 0)	4.04 (3 to 5.33)	0 (0 to 0)	5.61 (4.32 to 7.39)	-33.36% (-54.87% to -2.76%)	38.86% (-5.95% to 102.62%)	-2.4% (-3.5% to -1.29%)
North Macedonia	27 (21 to 34)	5.22 (4.05 to 6.49)	1 (1 to 1)	0.22 (0.17 to 0.31)	-97.34% (-98.19% to -95.82%)	-95.72% (-97.09% to -93.28%)	-8.33% (-8.94% to -7.71%)
Northern Mariana Islands	0 (0 to 0)	0.8 (0.59 to 1.08)	0 (0 to 0)	0.44 (0.34 to 0.57)	-49.28% (-63.63% to -32.47%)	-45.25% (-60.74% to -27.1%)	-1.65% (-2.13% to -1.18%)
Norway	17 (16 to 18)	2.16 (1.99 to 2.32)	2 (2 to 3)	0.26 (0.22 to 0.3)	-86.12% (-88.24% to -83.96%)	-88% (-89.83% to -86.14%)	-6.69% (-7.23% to -6.15%)
Oman	20 (15 to 28)	2.44 (1.79 to 3.34)	3 (2 to 4)	0.27 (0.2 to 0.36)	-83.69% (-88.86% to -75.9%)	-88.79% (-92.35% to -83.44%)	-6.05% (-6.89% to -5.21%)
Pakistan	20828 (16613 to 25402)	42.3 (33.74 to 51.59)	9831 (6905 to 14375)	11.51 (8.08 to 16.82)	-52.8% (-68.41% to -29.25%)	-72.8% (-81.79% to -59.22%)	-3.44% (-3.77% to -3.12%)
Palau	0 (0 to 0)	4.68 (3.22 to 6.34)	0 (0 to 0)	0.96 (0.69 to 1.38)	-85.32% (-90.26% to -77.71%)	-79.49% (-86.39% to -68.85%)	-4.91% (-5.26% to -4.56%)
Palestine	43 (31 to 60)	4.47 (3.2 to 6.17)	10 (7 to 13)	0.52 (0.37 to 0.69)	-77.79% (-84.84% to -65.7%)	-88.48% (-92.14% to -82.21%)	-6.44% (-6.92% to -5.96%)
Panama	49 (42 to 57)	5.92 (5.07 to 6.83)	14 (11 to 19)	1.25 (0.97 to 1.61)	-70.77% (-78.15% to -61.06%)	-78.87% (-84.2% to -71.85%)	-4.87% (-5.14% to -4.61%)
Papua New Guinea	224 (167 to 304)	13.19 (9.85 to 17.86)	190 (119 to 299)	4.84 (3.05 to 7.64)	-15.35% (-49.8% to 42.36%)	-63.26% (-78.21% to -38.21%)	-4.88% (-5.6% to -4.15%)
Paraguay	121 (97 to 148)	7.25 (5.78 to 8.85)	27 (18 to 40)	1.36 (0.91 to 1.99)	-77.46% (-85.76% to -63.96%)	-81.26% (-88.16% to -70.03%)	-5.56% (-5.94% to -5.19%)
Peru	414 (346 to 494)	4.99 (4.17 to 5.95)	45 (31 to 63)	0.47 (0.32 to 0.67)	-89.16% (-92.71% to -83.32%)	-90.56% (-93.66% to -85.49%)	-7.54% (-7.83% to -7.26%)
Philippines	3280 (2358 to 3960)	13.01 (9.35 to 15.71)	1116 (880 to 1449)	3.28 (2.59 to 4.26)	-65.97% (-74.98% to -50.47%)	-74.76% (-81.45% to -63.26%)	-3.59% (-3.95% to -3.23%)

Location	Deaths in 1990	Rate in 1990 (per 100,000)	Deaths in 2021	Rate in 2021 (per 100,000)	Changes of deaths, 1990-2021	Changes of rates, 1990-2021	EAPC 1990-2021
Poland	245 (229 to 262)	2.56 (2.39 to 2.74)	11 (8 to 13)	0.18 (0.14 to 0.23)	-95.7% (-96.65% to -94.44%)	-93% (-94.55% to -90.96%)	-6.48% (-7.45% to -5.51%)
Portugal	52 (48 to 56)	2.44 (2.26 to 2.63)	4 (4 to 5)	0.32 (0.26 to 0.39)	-91.58% (-93.13% to -89.65%)	-86.93% (-89.33% to -83.93%)	-7.09% (-7.74% to -6.43%)
Puerto Rico	13 (12 to 14)	1.32 (1.2 to 1.45)	0 (0 to 0)	0.06 (0.05 to 0.07)	-98.02% (-98.36% to -97.62%)	-95.56% (-96.33% to -94.66%)	-9.3% (-10.21% to -8.38%)
Qatar	2 (1 to 3)	1.27 (0.81 to 2.09)	1 (1 to 1)	0.2 (0.15 to 0.26)	-38.55% (-63.86% to -0.88%)	-84.44% (-90.85% to -74.91%)	-5.19% (-5.59% to -4.8%)
Republic of Korea	153 (121 to 187)	1.35 (1.07 to 1.64)	4 (3 to 5)	0.06 (0.05 to 0.08)	-97.6% (-98.28% to -96.4%)	-95.5% (-96.78% to -93.26%)	-9.14% (-9.49% to -8.78%)
Republic of Moldova	98 (87 to 111)	7.96 (7.08 to 8.99)	6 (4 to 8)	1.15 (0.84 to 1.55)	-93.88% (-95.65% to -91.74%)	-85.53% (-89.7% to -80.45%)	-4.34% (-4.96% to -3.71%)
Romania	312 (286 to 340)	5.61 (5.14 to 6.1)	10 (8 to 13)	0.34 (0.28 to 0.44)	-96.73% (-97.35% to -95.81%)	-93.95% (-95.11% to -92.24%)	-7.88% (-8.29% to -7.47%)
Russian Federation	1190 (1152 to 1234)	3.43 (3.32 to 3.56)	140 (125 to 156)	0.54 (0.48 to 0.6)	-88.23% (-89.49% to -86.87%)	-84.33% (-86.01% to -82.53%)	-4.64% (-5.29% to -3.98%)
Rwanda	2315 (1513 to 3354)	68.24 (44.59 to 98.85)	403 (266 to 612)	8.1 (5.34 to 12.31)	-82.6% (-88.51% to -71.5%)	-88.12% (-92.16% to -80.54%)	-7.97% (-8.65% to -7.28%)
Saint Kitts and Nevis	1 (1 to 1)	6.37 (5.64 to 7.22)	0 (0 to 0)	1.17 (0.92 to 1.48)	-87.23% (-90.19% to -83.52%)	-81.69% (-85.92% to -76.37%)	-5.57% (-6.05% to -5.09%)
Saint Lucia	2 (2 to 3)	4.85 (4.04 to 5.76)	0 (0 to 0)	0.76 (0.56 to 1.02)	-91.01% (-93.67% to -87.53%)	-84.4% (-89.02% to -78.35%)	-6.35% (-6.75% to -5.95%)
Saint Vincent and the Grenadines	3 (2 to 3)	6.45 (5.28 to 7.8)	0 (0 to 0)	0.86 (0.67 to 1.11)	-91.93% (-94.07% to -89.05%)	-86.71% (-90.23% to -81.96%)	-6.76% (-7.16% to -6.36%)
Samoa	3 (2 to 5)	4.9 (3.32 to 6.88)	1 (1 to 2)	1.46 (0.94 to 2.14)	-66.46% (-78.98% to -48.23%)	-70.11% (-81.27% to -53.86%)	-3.8% (-3.92% to -3.67%)
San Marino	0 (0 to 0)	1.22 (0.79 to 1.8)	0 (0 to 0)	0.11 (0.07 to 0.17)	-90.46% (-94.33% to -83.39%)	-91.12% (-94.71% to -84.52%)	-7.22% (-7.48% to -6.96%)
Sao Tome and Principe	8 (6 to 11)	14.63 (11.2 to 19.08)	1 (1 to 1)	1.13 (0.78 to 1.77)	-89.4% (-93.13% to -83.13%)	-92.28% (-95% to -87.71%)	-8.38% (-9.11% to -7.63%)
Saudi Arabia	228 (165 to 323)	3.49 (2.52 to 4.93)	12 (8 to 17)	0.15 (0.11 to 0.22)	-94.91% (-96.71% to -92.32%)	-95.59% (-97.15% to -93.35%)	-10.02% (-10.37% to -9.68%)
Senegal	2060 (1574 to 2750)	56.43 (43.11 to 75.31)	509 (360 to 828)	8.01 (5.66 to 13.02)	-75.29% (-82.92% to -60.12%)	-85.81% (-90.2% to -77.11%)	-6.27% (-6.95% to -5.58%)
Serbia	43 (32 to 55)	1.98 (1.48 to 2.54)	2 (1 to 2)	0.11 (0.08 to 0.16)	-96.47% (-97.62% to -94.73%)	-94.24% (-96.11% to -91.39%)	-9.24% (-9.88% to -8.59%)

Location	Deaths in 1990	Rate in 1990 (per 100,000)	Deaths in 2021	Rate in 2021 (per 100,000)	Changes of deaths, 1990-2021	Changes of rates, 1990-2021	EAPC 1990-2021
Seychelles	3 (3 to 4)	14.57 (13.37 to 15.98)	0 (0 to 1)	1.72 (1.25 to 2.37)	-88.38% (-91.64% to -83.24%)	-88.22% (-91.52% to -83.01%)	-4.61% (-6.66% to -2.52%)
Sierra Leone	1965 (1258 to 2813)	108.4 (69.43 to 155.21)	504 (354 to 715)	14.1 (9.89 to 20)	-74.34% (-83.79% to -59.11%)	-86.99% (-91.78% to -79.27%)	-7.14% (-7.66% to -6.62%)
Singapore	8 (7 to 8)	1.16 (1.06 to 1.27)	1 (1 to 1)	0.1 (0.09 to 0.12)	-89% (-91.18% to -86.61%)	-91.2% (-92.95% to -89.29%)	-7.01% (-7.72% to -6.29%)
Slovakia	18 (15 to 22)	1.38 (1.15 to 1.64)	2 (2 to 3)	0.29 (0.21 to 0.4)	-86.57% (-90.44% to -80.08%)	-79.22% (-85.2% to -69.17%)	-4.88% (-5.18% to -4.58%)
Slovenia	4 (4 to 5)	1.06 (0.94 to 1.18)	0 (0 to 0)	0.1 (0.08 to 0.13)	-92.7% (-94.25% to -90.92%)	-90.33% (-92.39% to -87.98%)	-6.3% (-6.61% to -5.99%)
Solomon Islands	12 (7 to 17)	7.39 (4.79 to 10.9)	5 (3 to 6)	1.75 (1.3 to 2.4)	-60.4% (-73.86% to -34.25%)	-76.29% (-84.35% to -60.63%)	-4.37% (-4.97% to -3.77%)
Somalia	2128 (1411 to 3131)	54.63 (36.21 to 80.37)	2582 (1564 to 4417)	25 (15.14 to 42.76)	21.32% (-24.37% to 97.69%)	-54.25% (-71.48% to -25.44%)	-2.01% (-2.42% to -1.6%)
South Africa	1604 (1192 to 1993)	11.78 (8.76 to 14.64)	665 (523 to 815)	4.37 (3.44 to 5.36)	-58.55% (-69.84% to -42.45%)	-62.89% (-73% to -48.47%)	-2.89% (-3.24% to -2.54%)
South Sudan	1668 (1073 to 2226)	63.57 (40.89 to 84.83)	1570 (1078 to 2161)	36.56 (25.1 to 50.32)	-5.87% (-37.47% to 46.69%)	-42.49% (-61.8% to -10.37%)	-1.4% (-1.71% to -1.09%)
Spain	123 (116 to 131)	1.57 (1.48 to 1.67)	12 (10 to 16)	0.18 (0.15 to 0.25)	-90.3% (-92.42% to -86.85%)	-88.27% (-90.84% to -84.1%)	-6.71% (-7.22% to -6.2%)
Sri Lanka	209 (169 to 271)	3.77 (3.06 to 4.89)	67 (49 to 89)	1.31 (0.95 to 1.75)	-67.83% (-79.49% to -52.36%)	-65.13% (-77.76% to -48.36%)	-2.8% (-3.45% to -2.14%)
Sudan	2024 (1592 to 2664)	22.76 (17.9 to 29.96)	277 (174 to 433)	1.67 (1.05 to 2.61)	-86.32% (-91.05% to -78.84%)	-92.67% (-95.2% to -88.66%)	-7.5% (-8.44% to -6.55%)
Suriname	14 (9 to 18)	10.68 (7.21 to 13.66)	3 (2 to 5)	2.33 (1.56 to 3.56)	-76% (-85.82% to -53.15%)	-78.18% (-87.11% to -57.4%)	-5.19% (-5.58% to -4.79%)
Sweden	11 (10 to 12)	0.72 (0.67 to 0.78)	4 (4 to 5)	0.23 (0.2 to 0.27)	-62.1% (-67.48% to -56.12%)	-67.85% (-72.42% to -62.78%)	-3.7% (-4.46% to -2.93%)
Switzerland	33 (30 to 36)	2.84 (2.63 to 3.1)	6 (5 to 7)	0.46 (0.38 to 0.54)	-81.42% (-84.9% to -77.36%)	-83.88% (-86.91% to -80.37%)	-5.88% (-6.21% to -5.55%)
Syrian Arab Republic	704 (529 to 927)	11.89 (8.94 to 15.66)	65 (48 to 85)	1.78 (1.31 to 2.32)	-90.75% (-94% to -86.4%)	-85.04% (-90.3% to -78.01%)	-5.69% (-6.4% to -4.97%)
Taiwan (Province of China)	82 (78 to 87)	1.5 (1.41 to 1.58)	9 (7 to 10)	0.29 (0.25 to 0.33)	-89.65% (-91.18% to -87.97%)	-80.66% (-83.52% to -77.51%)	-5.58% (-6.45% to -4.71%)
Tajikistan	433 (362 to 512)	18.66 (15.58 to 22.07)	143 (85 to 224)	3.98 (2.37 to 6.25)	-67.11% (-81.23% to -47.22%)	-78.7% (-87.84% to -65.81%)	-4.95% (-5.22% to -4.68%)

Location	Deaths in 1990	Rate in 1990 (per 100,000)	Deaths in 2021	Rate in 2021 (per 100,000)	Changes of deaths, 1990-2021	Changes of rates, 1990-2021	EAPC 1990-2021
Thailand	856 (625 to 1159)	5.08 (3.71 to 6.88)	87 (66 to 115)	0.89 (0.67 to 1.17)	-89.82% (-93.31% to -84.29%)	-82.44% (-88.46% to -72.89%)	-5.52% (-5.96% to -5.09%)
Timor-Leste	91 (59 to 143)	27.46 (17.79 to 42.88)	30 (22 to 43)	5.82 (4.15 to 8.32)	-66.84% (-79.71% to -36.9%)	-78.81% (-87.03% to -59.68%)	-5.26% (-5.43% to -5.09%)
Togo	687 (531 to 888)	38.98 (30.13 to 50.36)	289 (161 to 512)	8.73 (4.87 to 15.47)	-57.93% (-74.61% to -31.94%)	-77.59% (-86.48% to -63.75%)	-4.76% (-5.49% to -4.02%)
Tokelau	0 (0 to 0)	5.3 (3.82 to 7.2)	0 (0 to 0)	9.73 (7.17 to 12.97)	19.23% (-18.16% to 75.8%)	83.39% (25.87% to 170.4%)	-2.23% (-3.82% to -0.62%)
Tonga	7 (5 to 9)	16.31 (12.34 to 21.06)	3 (2 to 4)	6.45 (4.51 to 9.02)	-63.07% (-76.68% to -44.88%)	-60.43% (-75.01% to -40.95%)	-3.01% (-3.33% to -2.69%)
Trinidad and Tobago	20 (17 to 23)	4.97 (4.2 to 5.77)	2 (2 to 3)	0.84 (0.58 to 1.23)	-88.73% (-92.6% to -83.1%)	-83.2% (-88.97% to -74.79%)	-5.82% (-6.19% to -5.45%)
Tunisia	134 (93 to 200)	4.33 (2.99 to 6.43)	10 (6 to 15)	0.36 (0.22 to 0.55)	-92.66% (-95.14% to -89.07%)	-91.76% (-94.54% to -87.73%)	-6.8% (-7.23% to -6.37%)
Turkey	1306 (898 to 1853)	6.37 (4.38 to 9.04)	65 (49 to 83)	0.35 (0.27 to 0.45)	-95.03% (-96.99% to -92.31%)	-94.5% (-96.68% to -91.49%)	-9.39% (-9.85% to -8.92%)
Turkmenista n	151 (133 to 171)	10.04 (8.87 to 11.4)	14 (11 to 18)	0.89 (0.69 to 1.2)	-91.01% (-93.17% to -87.78%)	-91.14% (-93.28% to -87.96%)	-7.23% (-8% to -6.46%)
Tuvalu	1 (0 to 1)	15.91 (11.58 to 21.12)	0 (0 to 0)	2.12 (1.44 to 3.21)	-85.7% (-90.7% to -77.46%)	-86.67% (-91.33% to -78.99%)	-6.29% (-6.49% to -6.08%)
Uganda	5372 (3380 to 8361)	63.8 (40.15 to 99.31)	2494 (1471 to 3982)	12.57 (7.42 to 20.08)	-53.57% (-69.78% to -24.55%)	-80.29% (-87.17% to -67.97%)	-5.15% (-5.47% to -4.83%)
Ukraine	423 (365 to 485)	3.72 (3.21 to 4.26)	43 (36 to 51)	0.68 (0.56 to 0.8)	-89.76% (-91.91% to -87.36%)	-81.64% (-85.5% to -77.34%)	-4.35% (-4.85% to -3.86%)
United Arab Emirates	25 (19 to 33)	4.26 (3.17 to 5.52)	5 (3 to 6)	0.34 (0.25 to 0.45)	-81.76% (-86.75% to -72.57%)	-91.97% (-94.17% to -87.92%)	-6.61% (-7% to -6.22%)
United Kingdom	249 (240 to 259)	2.28 (2.2 to 2.37)	42 (37 to 46)	0.35 (0.32 to 0.39)	-83.24% (-84.96% to -81.68%)	-84.47% (-86.06% to -83.02%)	-5.92% (-6.26% to -5.58%)
United Republic of Tanzania	5917 (3777 to 8575)	49 (31.28 to 71.01)	2120 (1118 to 3440)	8.69 (4.58 to 14.1)	-64.17% (-75.28% to -38.45%)	-82.27% (-87.77% to -69.54%)	-5.15% (-5.69% to -4.6%)
United States of America	675 (643 to 714)	1.21 (1.15 to 1.28)	116 (104 to 129)	0.19 (0.18 to 0.22)	-82.85% (-84.69% to -81.01%)	-83.87% (-85.6% to -82.14%)	-5.69% (-6.03% to -5.34%)
United States Virgin Islands	0 (0 to 1)	1.34 (0.95 to 1.87)	0 (0 to 0)	0.16 (0.1 to 0.23)	-95.13% (-97.12% to -92.3%)	-88.39% (-93.12% to -81.62%)	-6.28% (-6.66% to -5.9%)
Uruguay	26 (23 to 28)	3.12 (2.86 to 3.38)	3 (2 to 3)	0.44 (0.35 to 0.53)	-88.74% (-91.18% to -86.01%)	-86.02% (-89.05% to -82.64%)	-6.46% (-6.69% to -6.22%)

Location	Deaths in 1990	Rate in 1990 (per 100,000)	Deaths in 2021	Rate in 2021 (per 100,000)	Changes of deaths, 1990-2021	Changes of rates, 1990-2021	EAPC 1990-2021
Uzbekistan	843 (745 to 958)	9.85 (8.7 to 11.2)	63 (51 to 77)	0.62 (0.5 to 0.77)	-92.53% (-94.05% to -90.57%)	-93.66% (-94.96% to -92%)	-8.9% (-9.44% to -8.37%)
Vanuatu	4 (3 to 6)	5.6 (3.82 to 8.72)	3 (2 to 4)	2.27 (1.52 to 3.77)	-30.51% (-52.42% to 2.26%)	-59.4% (-72.2% to -40.26%)	-2.95% (-3.63% to -2.26%)
Venezuela (Bolivarian Republic of)	493 (463 to 526)	6.96 (6.53 to 7.42)	144 (106 to 201)	2.18 (1.61 to 3.04)	-70.72% (-78.37% to -60.29%)	-68.64% (-76.84% to -57.47%)	-3.44% (-4.38% to -2.49%)
Viet Nam	2085 (1455 to 3040)	7.87 (5.49 to 11.47)	351 (219 to 559)	1.42 (0.89 to 2.26)	-83.15% (-89.58% to -73.89%)	-81.96% (-88.84% to -72.05%)	-5.05% (-5.3% to -4.8%)
Yemen	805 (545 to 1196)	11.35 (7.68 to 16.86)	229 (142 to 431)	1.66 (1.03 to 3.13)	-71.55% (-81.23% to -51.78%)	-85.36% (-90.34% to -75.19%)	-6.33% (-6.78% to -5.89%)
Zambia	3561 (2598 to 4714)	94.86 (69.19 to 125.56)	835 (516 to 1219)	10.1 (6.24 to 14.74)	-76.54% (-84.49% to -63.58%)	-89.35% (-92.96% to -83.47%)	-6.84% (-7.34% to -6.34%)
Zimbabwe	573 (446 to 728)	11.89 (9.27 to 15.12)	459 (319 to 700)	7.3 (5.07 to 11.12)	-19.79% (-45.87% to 26.19%)	-38.62% (-58.57% to -3.44%)	-1.28% (-1.9% to -0.67%)

EAPC, estimated annual percentage change

Table S5. Prevalence of impairments attributed to childhood meningitis in 1990 and 2021

Impairments	Prevalent cases in 1990	Rate in 1990 (per 100,000)	Prevalent cases in 2021	Rate in 2021 (per 100,000)	Changes of cases, 1990-2021	Changes of rates, 1990-2021	EAPC, 1990-2021
**Epilepsy**	254905 (211005 to 299777)	14.66 (12.13 to 17.24)	111514 (93093 to 131442)	5.54 (4.63 to 6.53)	-56.25% (-58.07 to -54.47)	-62.18% (-63.75 to -60.64)	-3.69% (-4.02 to -3.36)
**Hearing loss**	473505 (347709 to 619302)	27.23 (19.99 to 35.61)	249682 (190429 to 323026)	12.41 (9.47 to 16.06)	-47.27% (-49.43 to -44.36)	-54.42% (-56.28 to -51.91)	-2.88% (-3.21 to -2.55)
Mild hearing loss	161046 (92982 to 238304)	9.26 (5.35 to 13.7)	82715 (48578 to 120506)	4.11 (2.41 to 5.99)	-48.64% (-50.84 to -45.81)	-55.6% (-57.5 to -53.15)	-2.98% (-3.31 to -2.65)
Moderate hearing loss	181188 (119122 to 261688)	10.42 (6.85 to 15.05)	101981 (69657 to 144525)	5.07 (3.46 to 7.18)	-43.72% (-46.27 to -40.21)	-51.34% (-53.56 to -48.32)	-2.63% (-2.98 to -2.28)
Moderately severe hearing loss	36099 (21944 to 55500)	2.08 (1.26 to 3.19)	17959 (11221 to 27457)	0.89 (0.56 to 1.36)	-50.25% (-53.02 to -47.08)	-56.99% (-59.39 to -54.25)	-3.21% (-3.52 to -2.9)
Severe hearing loss	39824 (22061 to 65285)	2.29 (1.27 to 3.75)	20212 (11210 to 32102)	1 (0.56 to 1.6)	-49.25% (-52.16 to -46.07)	-56.13% (-58.64 to -53.38)	-3.03% (-3.33 to -2.73)
Profound hearing loss	46834 (29139 to 72996)	2.69 (1.68 to 4.2)	22872 (14376 to 36174)	1.14 (0.71 to 1.8)	-51.16% (-53.85 to -48)	-57.78% (-60.11 to -55.05)	-3.12% (-3.41 to -2.83)
Complete hearing loss	8514 (5267 to 14408)	0.49 (0.3 to 0.83)	3943 (2409 to 6438)	0.2 (0.12 to 0.32)	-53.69% (-56.22 to -50.78)	-59.97% (-62.16 to -57.46)	-3.41% (-3.73 to -3.09)
**Developmental intellectual disability**	1055477 (754938 to 1459823)	60.69 (43.41 to 83.94)	552778 (400800 to 757980)	27.48 (19.92 to 37.68)	-47.63% (-51.14 to -44.3)	-54.73% (-57.76 to -51.85)	-3.05% (-3.42 to -2.68)
Borderline intellectual disability	564687 (359563 to 874958)	32.47 (20.67 to 50.31)	287727 (177414 to 452002)	14.3 (8.82 to 22.47)	-49.05% (-53 to -45.34)	-55.95% (-59.37 to -52.75)	-3.11% (-3.44 to -2.78)
Mild intellectual disability	347406 (264179 to 460277)	19.98 (15.19 to 26.47)	195258 (153360 to 252824)	9.71 (7.62 to 12.57)	-43.8% (-47.97 to -39.12)	-51.41% (-55.02 to -47.37)	-2.85% (-3.2 to -2.5)
Moderate intellectual disability	99639 (65816 to 138400)	5.73 (3.78 to 7.96)	46878 (34986 to 65189)	2.33 (1.74 to 3.24)	-52.95% (-58.75 to -44.41)	-59.33% (-64.34 to -51.94)	-3.41% (-4 to -2.82)
Severe intellectual disability	43745 (17994 to 77856)	2.52 (1.03 to 4.48)	22914 (10964 to 37545)	1.14 (0.54 to 1.87)	-47.62% (-60.39 to -27.05)	-54.72% (-65.76 to -36.93)	-3.24% (-3.75 to -2.72)
**Blindness and vision loss**	75641 (55510 to 99807)	4.35 (3.19 to 5.74)	39630 (29159 to 51344)	1.97 (1.45 to 2.55)	-47.61% (-50.17 to -44.27)	-54.71% (-56.93 to -51.82)	-3.07% (-3.41 to -2.73)
Moderate vision loss	50740 (33959 to 70539)	2.92 (1.95 to 4.06)	26747 (18172 to 36476)	1.33 (0.9 to 1.81)	-47.29% (-49.9 to -43.99)	-54.43% (-56.69 to -51.58)	-3.06% (-3.39 to -2.74)
Severe vision loss	14101 (8357 to 21443)	0.81 (0.48 to 1.23)	8304 (4812 to 12492)	0.41 (0.24 to 0.62)	-41.11% (-44.71 to -37.21)	-49.1% (-52.2 to -45.72)	-2.69% (-3.07 to -2.32)
Blindness	10799 (6350 to 17435)	0.62 (0.37 to 1)	4579 (2571 to 7268)	0.23 (0.13 to 0.36)	-57.59% (-62.16 to -52.93)	-63.34% (-67.29 to -59.31)	-3.7% (-4.08 to -3.32)

S6 Table. Etiology proportions (%) of childhood meningitis deaths by region in 2021

Location	Streptococcu spneumoniae	Neisseria meningitidis	Klebsiella pneumoniae	Viral etiologies	Other bacterial pathogen	Group B streptococcus	Escherichia coli	Staphylococc us aureus	Haemophilus influenzae	Listeria monocytogen es
Global	17.01% (15.78% to 18.21%)	14.03% (12.97% to 15.03%)	12.11% (10% to 14.56%)	10.53% (9.94% to 11.1%)	10.27% (7.88% to 13.23%)	9.49% (8.56% to 10.43%)	9.45% (8.1% to 11.07%)	6.28% (5.6% to 7.08%)	5.92% (5.47% to 6.41%)	4.8% (3.84% to 6.08%)
**5 SDI quintiles regions**										
High SDI	9.22% (8.54% to 9.91%)	8.15% (7.54% to 8.79%)	15.49% (13.59% to 17.37%)	8.9% (8.42% to 9.39%)	12.64% (9.28% to 16.81%)	13.67% (12.72% to 14.78%)	12.95% (11.65% to 14.27%)	7.42% (6.75% to 8.07%)	4.65% (4.2% to 5.08%)	6.9% (5.56% to 8.32%)
High-middle SDI	15.56% (14.71% to 16.53%)	12.23% (11.62% to 12.92%)	13.4% (11.72% to 15.24%)	9.19% (8.78% to 9.61%)	11.49% (8.86% to 14.66%)	10.07% (9.47% to 10.63%)	10.66% (9.56% to 11.7%)	6.14% (5.74% to 6.56%)	5.57% (5.2% to 5.92%)	5.65% (4.69% to 6.7%)
Middle SDI	17.1% (16.21% to 18.12%)	14.13% (13.26% to 14.96%)	12.47% (10.68% to 14.64%)	9.91% (9.47% to 10.38%)	10.81% (8.55% to 13.74%)	9.14% (8.49% to 9.87%)	9.67% (8.48% to 11.01%)	6.06% (5.56% to 6.58%)	5.57% (5.15% to 5.99%)	5.06% (4.13% to 6.17%)
Low-middle SDI	16.79% (15.75% to 17.94%)	14.96% (13.96% to 15.99%)	12.09% (10.1% to 14.47%)	10.36% (9.82% to 10.94%)	10.39% (8.1% to 13.42%)	9.3% (8.45% to 10.18%)	9.44% (8.1% to 11.01%)	6.26% (5.6% to 6.97%)	5.49% (5.08% to 5.93%)	4.83% (3.88% to 6.06%)
Low SDI	17.16% (15.71% to 18.53%)	13.64% (12.54% to 14.81%)	12.01% (9.79% to 14.74%)	10.75% (10.07% to 11.37%)	10.1% (7.73% to 13.04%)	9.6% (8.6% to 10.62%)	9.38% (7.93% to 11.1%)	6.31% (5.57% to 7.19%)	6.18% (5.73% to 6.75%)	4.72% (3.72% to 6.05%)
**21 GBD regions**										
Andean Latin America	13.42% (12.49% to 14.42%)	12.35% (11.58% to 13.18%)	13.64% (11.87% to 15.69%)	10.34% (9.79% to 10.86%)	11.2% (8.74% to 14.07%)	11.48% (10.43% to 12.61%)	10.75% (9.58% to 12.08%)	6.92% (6.31% to 7.53%)	4.26% (3.89% to 4.66%)	5.58% (4.65% to 6.69%)
Australasia	7.53% (6.91% to 8.2%)	7.29% (6.66% to 7.92%)	16.1% (13.98% to 18.08%)	8.73% (8.18% to 9.3%)	12.53% (9.07% to 17.05%)	14.85% (13.68% to 16.15%)	13.45% (12.02% to 14.98%)	7.63% (6.86% to 8.46%)	4.75% (4.25% to 5.24%)	7.12% (5.67% to 8.64%)

Location	Streptococcu spneumoniae	Neisseria meningitidis	Klebsiella pneumoniae	Viral etiologies	Other bacterial pathogen	Group B streptococcus	Escherichia coli	Staphylococc us aureus	Haemophilus influenzae	Listeria monocytogen es
Caribbean	18.13% (16.02% to 20.03%)	16.17% (14.75% to 17.49%)	11.76% (9.55% to 14.07%)	10.21% (9.36% to 11.23%)	8.57% (6.48% to 11.46%)	10.64% (9.14% to 12.61%)	9.24% (7.89% to 10.84%)	5.5% (4.88% to 6.13%)	5.35% (4.96% to 5.8%)	4.41% (3.49% to 5.54%)
Central Asia	16.92% (15.71% to 18.19%)	13.15% (12.45% to 13.92%)	12.98% (11.22% to 14.94%)	9.78% (9.27% to 10.3%)	10.33% (8.07% to 13.13%)	11.08% (10.26% to 11.89%)	10.29% (9.15% to 11.48%)	6.13% (5.61% to 6.65%)	4.12% (3.8% to 4.44%)	5.21% (4.3% to 6.28%)
Central Europe	12.98% (12.23% to 13.83%)	9.5% (8.91% to 10.1%)	14.59% (12.98% to 16.39%)	8.86% (8.39% to 9.31%)	11.22% (8.32% to 14.77%)	13.37% (12.63% to 14.15%)	12.15% (11.06% to 13.26%)	6.47% (5.99% to 6.98%)	4.66% (4.3% to 5.04%)	6.17% (5.09% to 7.38%)
Central Latin America	13.92% (13.14% to 14.83%)	11.84% (11.18% to 12.55%)	13.64% (11.95% to 15.63%)	9.7% (9.24% to 10.18%)	11.31% (8.7% to 14.4%)	11.44% (10.66% to 12.23%)	10.99% (9.85% to 12.25%)	6.68% (6.16% to 7.23%)	4.74% (4.37% to 5.12%)	5.71% (4.71% to 6.87%)
Central Sub-Saharan Africa	15.45% (14.36% to 16.67%)	19.46% (18.17% to 20.91%)	11.53% (9.47% to 13.86%)	11.32% (10.23% to 12.07%)	9.53% (7.67% to 11.85%)	8.72% (7.74% to 9.72%)	8.4% (7.02% to 10.01%)	6.06% (5.32% to 6.79%)	5.16% (4.67% to 5.78%)	4.31% (3.48% to 5.4%)
East Asia	16.23% (15.35% to 17.14%)	11.28% (10.69% to 11.86%)	13.79% (12.21% to 15.5%)	8.83% (8.36% to 9.31%)	12.42% (9.54% to 15.86%)	9.29% (8.72% to 9.86%)	10.86% (9.78% to 11.88%)	6.01% (5.59% to 6.4%)	5.27% (4.85% to 5.69%)	6% (4.98% to 7.16%)
Eastern Europe	12.04% (11.3% to 12.79%)	10.44% (9.87% to 11.05%)	14.12% (12.38% to 16.07%)	9.03% (8.61% to 9.48%)	11.45% (8.69% to 14.94%)	11.11% (10.51% to 11.73%)	11.4% (10.3% to 12.56%)	6.23% (5.74% to 6.7%)	8.25% (7.69% to 8.84%)	5.93% (4.88% to 7.09%)
Eastern Sub-Saharan Africa	16.25% (15.07% to 17.51%)	14.94% (13.83% to 16.03%)	12.08% (9.89% to 14.62%)	11.18% (10.47% to 11.79%)	9.83% (7.68% to 12.46%)	9.94% (8.9% to 11%)	9.24% (7.79% to 10.92%)	6.41% (5.66% to 7.24%)	5.34% (4.91% to 5.83%)	4.65% (3.7% to 5.88%)
High-income Asia Pacific	9.1% (8.45% to 9.83%)	8.1% (7.52% to 8.7%)	15.69% (13.87% to 17.5%)	8.86% (8.39% to 9.35%)	12.58% (9.27% to 16.69%)	12.99% (12.15% to 13.92%)	12.94% (11.68% to 14.17%)	7.03% (6.42% to 7.67%)	5.83% (5.32% to 6.39%)	6.87% (5.62% to 8.28%)
High-income North America	8.05% (7.42% to 8.77%)	7.98% (7.34% to 8.67%)	15.58% (13.62% to 17.52%)	9.03% (8.49% to 9.57%)	12.82% (9.42% to 17.14%)	13.82% (12.79% to 15.01%)	13.08% (11.72% to 14.49%)	7.75% (7.03% to 8.52%)	4.89% (4.39% to 5.36%)	6.99% (5.6% to 8.54%)

Location	Streptococcu spneumoniae	Neisseria meningitidis	Klebsiella pneumoniae	Viral etiologies	Other bacterial pathogen	Group B streptococcus	Escherichia coli	Staphylococc us aureus	Haemophilus influenzae	Listeria monocytogen es
North Africa and Middle East	15.69% (14.65% to 16.91%)	14.3% (13.29% to 15.4%)	12.42% (10.39% to 14.8%)	10.25% (9.63% to 10.84%)	10.05% (7.71% to 12.98%)	10.33% (9.36% to 11.35%)	9.89% (8.47% to 11.41%)	6.25% (5.59% to 6.95%)	5.83% (5.41% to 6.33%)	4.94% (3.97% to 6.09%)
Oceania	17.51% (16.2% to 18.8%)	16.93% (15.83% to 18.1%)	11.15% (8.94% to 13.94%)	9.55% (8.97% to 10.15%)	10.01% (7.3% to 13.38%)	8.58% (7.69% to 9.54%)	9% (7.47% to 10.8%)	5.88% (5.18% to 6.57%)	6.83% (6.27% to 7.44%)	4.54% (3.5% to 5.83%)
South Asia	17.33% (16.24% to 18.58%)	15.95% (14.98% to 16.95%)	11.85% (9.9% to 14.19%)	10.05% (9.52% to 10.63%)	10.34% (8% to 13.3%)	8.96% (8.16% to 9.88%)	9.33% (7.97% to 10.92%)	6.12% (5.51% to 6.8%)	5.23% (4.88% to 5.68%)	4.77% (3.81% to 6.01%)
Southeast Asia	18.05% (16.98% to 19.14%)	15.22% (14.44% to 16.1%)	11.94% (10.06% to 14.32%)	9.44% (8.96% to 9.93%)	10.59% (8.08% to 13.7%)	8.72% (8% to 9.41%)	9.51% (8.19% to 10.96%)	5.87% (5.32% to 6.43%)	5.72% (5.34% to 6.13%)	4.9% (3.88% to 6.1%)
Southern Latin America	13.05% (12.29% to 13.89%)	10.85% (10.22% to 11.51%)	14.22% (12.53% to 16.06%)	9.72% (9.26% to 10.21%)	11.64% (8.92% to 14.81%)	12.11% (11.31% to 12.94%)	11.44% (10.35% to 12.66%)	6.89% (6.38% to 7.43%)	4.09% (3.76% to 4.42%)	5.99% (5% to 7.07%)
Southern Sub-Saharan Africa	14.79% (13.86% to 15.83%)	15.47% (14.5% to 16.4%)	12.5% (10.53% to 14.8%)	11.03% (10.44% to 11.61%)	10.99% (8.85% to 13.69%)	9.38% (8.5% to 10.3%)	9.46% (8.08% to 11.01%)	6.77% (6.08% to 7.49%)	4.55% (4.2% to 5%)	5% (4.09% to 6.12%)
Tropical Latin America	13.97% (13.08% to 14.9%)	12.17% (11.45% to 12.93%)	13.32% (11.44% to 15.41%)	9.81% (9.33% to 10.29%)	12.33% (9.48% to 15.73%)	10.38% (9.6% to 11.17%)	10.97% (9.7% to 12.41%)	7.19% (6.59% to 7.86%)	3.95% (3.62% to 4.28%)	5.9% (4.87% to 7.19%)
Western Europe	9.36% (8.7% to 10.09%)	7.71% (7.13% to 8.32%)	15.67% (13.76% to 17.59%)	8.65% (8.15% to 9.14%)	12.64% (9.17% to 16.98%)	14.07% (13.04% to 15.23%)	13.21% (11.88% to 14.64%)	7.3% (6.62% to 7.99%)	4.36% (3.9% to 4.79%)	7.02% (5.65% to 8.47%)
Western Sub-Saharan Africa	17.49% (15.83% to 19.06%)	11.94% (10.67% to 13.27%)	12.2% (9.89% to 15.06%)	10.69% (9.97% to 11.41%)	10.36% (7.93% to 13.43%)	9.64% (8.5% to 10.9%)	9.58% (8.08% to 11.37%)	6.37% (5.61% to 7.29%)	6.74% (6.19% to 7.39%)	4.84% (3.77% to 6.23%)

S7 Table. Etiology proportions (%) of childhood meningitis deaths by region in 1990.

Location	Streptococcus pneumoniae	Neisseria meningitidis	Klebsiella pneumoniae	Viral etiologies	Other bacterial pathogen	Group B streptococcus	Escherichia coli	Staphylococcus aureus	Haemophilus influenzae	Listeria monocytogenes
Global	16.09% (15.01% to 17.21%)	19.95% (18.72% to 21.17%)	10.44% (8.3% to 13.15%)	9.76% (9.26% to 10.33%)	8.91% (6.6% to 11.82%)	7.62% (6.87% to 8.41%)	7.87% (6.37% to 9.63%)	5.17% (4.5% to 5.93%)	10.16% (9.36% to 11.02%)	3.96% (3.03% to 5.12%)
**5 SDI quintiles regions**										
High SDI	12.32% (11.61% to 13.04%)	15.64% (14.87% to 16.5%)	12.65% (10.83% to 14.54%)	8.34% (7.96% to 8.75%)	10.46% (7.86% to 13.78%)	9.27% (8.79% to 9.83%)	9.9% (8.79% to 11.15%)	5.33% (4.9% to 5.81%)	10.87% (10.19% to 11.5%)	5.21% (4.21% to 6.28%)
High-middle SDI	15.12% (14.19% to 16.08%)	16.07% (15.12% to 17.02%)	11.51% (9.45% to 14%)	9.21% (8.74% to 9.73%)	9.9% (7.22% to 13.08%)	8.26% (7.64% to 8.95%)	8.84% (7.52% to 10.41%)	5.37% (4.81% to 6.02%)	11.13% (10.35% to 11.95%)	4.55% (3.59% to 5.7%)
Middle SDI	15.76% (14.76% to 16.81%)	17.82% (16.71% to 18.89%)	10.99% (8.86% to 13.48%)	9.48% (8.98% to 10.02%)	9.62% (7.12% to 12.67%)	7.72% (7.05% to 8.43%)	8.33% (6.89% to 9.95%)	5.3% (4.72% to 6.01%)	10.64% (9.82% to 11.47%)	4.28% (3.33% to 5.41%)
Low-middle SDI	16.11% (15.05% to 17.23%)	20.96% (19.68% to 22.17%)	10.29% (8.16% to 12.92%)	9.81% (9.28% to 10.38%)	8.83% (6.62% to 11.63%)	7.41% (6.63% to 8.19%)	7.68% (6.2% to 9.42%)	5.13% (4.47% to 5.92%)	9.83% (9.05% to 10.72%)	3.88% (2.95% to 5.04%)
Low SDI	16.41% (15.2% to 17.61%)	20.64% (19.3% to 22%)	10.16% (7.95% to 13.06%)	9.93% (9.36% to 10.52%)	8.5% (6.21% to 11.5%)	7.64% (6.8% to 8.49%)	7.67% (6.12% to 9.48%)	5.11% (4.39% to 5.91%)	10.08% (9.21% to 11.01%)	3.79% (2.86% to 5.01%)
**21 GBD regions**										
Andean Latin America	15.44% (14.44% to 16.49%)	17.67% (16.58% to 18.75%)	11.17% (9.06% to 13.65%)	10.13% (9.53% to 10.76%)	8.98% (6.71% to 11.63%)	8.5% (7.66% to 9.38%)	8.38% (6.98% to 9.98%)	5.32% (4.69% to 5.99%)	10.22% (9.47% to 11%)	4.19% (3.29% to 5.33%)
Australasia	11.53% (10.78% to 12.25%)	10.16% (9.55% to 10.74%)	13.8% (11.87% to 15.88%)	8.17% (7.73% to 8.62%)	11.03% (8.1% to 14.8%)	10.59% (10% to 11.27%)	11.02% (9.84% to 12.41%)	5.53% (5.05% to 6.11%)	12.39% (11.54% to 13.21%)	5.77% (4.65% to 6.99%)

Location	Streptococcus pneumoniae	Neisseria meningitidis	Klebsiella pneumoniae	Viral etiologies	Other bacterial pathogen	Group B streptococcus	Escherichi a coli	Staphylococ cus aureus	Haemophilus influenzae	Listeria monocytoge nes
Caribbean	14.86% (13.63% to 16.31%)	20.28% (18.87% to 21.65%)	10.72% (8.55% to 13.17%)	9.92% (9.14% to 10.75%)	8.25% (6.11% to 11.07%)	8.62% (7.56% to 9.92%)	8.16% (6.79% to 9.83%)	5.08% (4.43% to 5.79%)	10.12% (9.33% to 11.02%)	3.99% (3.1% to 5.1%)
Central Asia	14.04% (13.2% to 14.91%)	18.07% (17.07% to 19.1%)	11.5% (9.61% to 13.79%)	9.16% (8.77% to 9.66%)	9.36% (6.97% to 12.36%)	8.68% (8.04% to 9.34%)	8.82% (7.57% to 10.23%)	5.24% (4.68% to 5.83%)	10.64% (9.94% to 11.32%)	4.48% (3.57% to 5.55%)
Central Europe	13.44% (12.62% to 14.23%)	13.54% (12.82% to 14.28%)	12.5% (10.6% to 14.66%)	8.87% (8.46% to 9.35%)	10.09% (7.43% to 13.45%)	9.59% (8.99% to 10.2%)	9.8% (8.59% to 11.17%)	5.44% (4.96% to 6%)	11.72% (10.98% to 12.47%)	5.01% (3.99% to 6.14%)
Central Latin America	14.9% (14.07% to 15.83%)	15.8% (14.91% to 16.75%)	11.68% (9.64% to 14%)	9.54% (9.09% to 10.06%)	9.69% (7.14% to 12.74%)	8.64% (7.98% to 9.32%)	8.91% (7.58% to 10.38%)	5.4% (4.84% to 6.05%)	10.89% (10.2% to 11.64%)	4.54% (3.62% to 5.67%)
Central Sub-Saharan Africa	15.89% (14.78% to 17.12%)	22.35% (20.75% to 23.99%)	10.08% (7.84% to 12.8%)	10.27% (9.6% to 11.04%)	8.14% (6.03% to 10.92%)	7.69% (6.77% to 8.66%)	7.47% (5.95% to 9.24%)	5.04% (4.34% to 5.8%)	9.38% (8.47% to 10.27%)	3.68% (2.77% to 4.82%)
East Asia	15.77% (14.74% to 16.82%)	16.43% (15.39% to 17.44%)	11.19% (9% to 13.75%)	9.15% (8.63% to 9.68%)	10.12% (7.4% to 13.42%)	7.63% (6.98% to 8.31%)	8.59% (7.15% to 10.26%)	5.37% (4.81% to 6.05%)	11.22% (10.3% to 12.13%)	4.47% (3.47% to 5.66%)
Eastern Europe	12.66% (11.89% to 13.41%)	13.64% (12.92% to 14.39%)	12.81% (10.94% to 14.92%)	9.02% (8.61% to 9.52%)	9.57% (7.05% to 12.75%)	10.49% (9.92% to 11.15%)	10.01% (8.88% to 11.31%)	5.42% (4.93% to 6%)	11.36% (10.67% to 12.04%)	5.01% (4.01% to 6.09%)
Eastern Sub-Saharan Africa	16.29% (15.02% to 17.49%)	21.79% (20.32% to 23.24%)	9.98% (7.77% to 12.77%)	9.95% (9.35% to 10.58%)	8.27% (6.09% to 11.18%)	7.58% (6.71% to 8.47%)	7.51% (5.94% to 9.33%)	5.03% (4.33% to 5.84%)	9.85% (8.98% to 10.72%)	3.7% (2.76% to 4.9%)
High-income Asia Pacific	12.7% (12.06% to 13.33%)	12.61% (12.01% to 13.23%)	13.47% (11.89% to 15.31%)	9.63% (9.22% to 10.1%)	10.81% (8.47% to 13.67%)	9.64% (9.12% to 10.2%)	9.98% (8.92% to 11.14%)	5.69% (5.27% to 6.17%)	10.17% (9.48% to 10.85%)	5.3% (4.42% to 6.21%)
High-income North America	10.8% (10.09% to 11.46%)	20.73% (19.34% to 22.22%)	12.02% (10.26% to 13.91%)	7.24% (6.87% to 7.62%)	10.05% (7.41% to 13.34%)	8.82% (8.32% to 9.39%)	9.61% (8.52% to 10.75%)	4.92% (4.5% to 5.4%)	10.7% (9.93% to 11.43%)	5.09% (4.08% to 6.21%)
North Africa and Middle East	14.64% (13.61% to 15.68%)	21.09% (19.63% to 22.78%)	10.68% (8.67% to 13.06%)	9.42% (8.89% to 10%)	8.66% (6.42% to 11.42%)	8.14% (7.34% to 8.97%)	8.12% (6.82% to 9.7%)	5.06% (4.47% to 5.73%)	10.07% (9.28% to 10.84%)	4.06% (3.21% to 5.11%)

Location	Streptococcus pneumoniae	Neisseria meningitidis	Klebsiella pneumoniae	Viral etiologies	Other bacterial pathogen	Group B streptococcus	Escherichia coli	Staphylococcus aureus	Haemophilus influenzae	Listeria monocytogenes
Oceania	16.7% (15.54% to 17.9%)	18.86% (17.6% to 20.2%)	10.41% (8.03% to 13.18%)	9.41% (8.83% to 10%)	9.31% (6.75% to 12.48%)	7.35% (6.53% to 8.24%)	7.95% (6.38% to 9.76%)	5.22% (4.57% to 6.03%)	10.73% (9.85% to 11.68%)	4.03% (3.03% to 5.27%)
South Asia	16.68% (15.57% to 17.84%)	19.96% (18.7% to 21.21%)	10.4% (8.18% to 13.11%)	10.2% (9.61% to 10.83%)	9.09% (6.92% to 11.93%)	7.22% (6.42% to 8.04%)	7.63% (6.11% to 9.42%)	5.26% (4.54% to 6.07%)	9.62% (8.82% to 10.59%)	3.89% (2.93% to 5.07%)
Southeast Asia	16.53% (15.39% to 17.7%)	18.46% (17.25% to 19.71%)	10.55% (8.2% to 13.28%)	9.37% (8.84% to 9.94%)	9.42% (6.81% to 12.59%)	7.42% (6.7% to 8.21%)	8.06% (6.52% to 9.87%)	5.24% (4.62% to 6.04%)	10.81% (9.95% to 11.77%)	4.1% (3.12% to 5.34%)
Southern Latin America	13.91% (13.07% to 14.76%)	14.46% (13.71% to 15.26%)	12.19% (10.29% to 14.39%)	9.09% (8.67% to 9.59%)	9.9% (7.32% to 13.11%)	9.27% (8.64% to 9.93%)	9.48% (8.25% to 10.86%)	5.41% (4.9% to 5.98%)	11.45% (10.69% to 12.24%)	4.83% (3.84% to 5.95%)
Southern Sub-Saharan Africa	15.4% (14.44% to 16.42%)	17.45% (16.48% to 18.57%)	11.3% (9.29% to 13.72%)	9.97% (9.41% to 10.6%)	9.45% (7.2% to 12.31%)	8.16% (7.44% to 8.96%)	8.42% (7.06% to 10.01%)	5.37% (4.77% to 6.04%)	10.15% (9.37% to 10.96%)	4.3% (3.4% to 5.4%)
Tropical Latin America	14.18% (13.32% to 15.12%)	23.68% (22.31% to 25.13%)	10.32% (8.37% to 12.61%)	8.37% (7.94% to 8.85%)	9.22% (6.78% to 12.2%)	7.1% (6.48% to 7.74%)	7.91% (6.64% to 9.38%)	4.9% (4.39% to 5.52%)	10.2% (9.47% to 10.96%)	4.11% (3.22% to 5.21%)
Western Europe	12.11% (11.37% to 12.78%)	13.05% (12.39% to 13.81%)	13.3% (11.47% to 15.24%)	8.2% (7.81% to 8.63%)	11.07% (8.27% to 14.63%)	9.66% (9.16% to 10.24%)	10.51% (9.37% to 11.74%)	5.47% (5.04% to 5.98%)	11.04% (10.32% to 11.74%)	5.58% (4.52% to 6.74%)
Western Sub-Saharan Africa	16.13% (14.91% to 17.35%)	20.33% (19% to 21.65%)	10.27% (8.08% to 13.03%)	9.71% (9.17% to 10.32%)	8.54% (6.19% to 11.51%)	7.82% (6.97% to 8.76%)	7.83% (6.26% to 9.64%)	5.1% (4.42% to 5.87%)	10.33% (9.47% to 11.25%)	3.87% (2.93% to 5.08%)

S8 Table. Global death counts and mortality rates of childhood meningitis by etiology, 1990 and 2021, with changes from 1990 to 2021.

Etiology	Death count in 1990	Death rate in 1990 (per 100,000)	Death count in 2021	Death rate in 2021 (per 100,000)	Change of number, 1990-2021	Change of rate, 1990-2021
Streptococcus pneumoniae	50732 (41973 to 60605)	2.92 (2.41 to 3.48)	19123 (13429 to 26978)	0.95 (0.67 to 1.34)	-62.31% (-71.22% to -49.13%)	-67.42% (-75.12% to -56.02%)
Neisseria meningitidis	62882 (52612 to 74737)	3.62 (3.03 to 4.3)	15741 (11475 to 21393)	0.78 (0.57 to 1.06)	-74.97% (-80.79% to -66.32%)	-78.36% (-83.39% to -70.88%)
Klebsiella pneumoniae	32911 (24819 to 42990)	1.89 (1.43 to 2.47)	13594 (9331 to 19202)	0.68 (0.46 to 0.95)	-58.7% (-68.44% to -44.56%)	-64.29% (-72.72% to -52.08%)
Viral etiologies of meningitis	30734 (26294 to 36277)	1.77 (1.51 to 2.09)	11817 (8708 to 16072)	0.59 (0.43 to 0.8)	-61.55% (-69.85% to -50.27%)	-66.76% (-73.94% to -57.01%)
Other bacterial pathogen	28076 (20277 to 38561)	1.61 (1.17 to 2.22)	11556 (7893 to 17136)	0.57 (0.39 to 0.85)	-58.84% (-68.69% to -44.12%)	-64.42% (-72.94% to -51.69%)
Group B streptococcus	24015 (19953 to 29014)	1.38 (1.15 to 1.67)	10652 (7603 to 14737)	0.53 (0.38 to 0.73)	-55.65% (-65.08% to -41.72%)	-61.66% (-69.82% to -49.62%)
Escherichia coli	24793 (19165 to 31229)	1.43 (1.1 to 1.8)	10618 (7397 to 15225)	0.53 (0.37 to 0.76)	-57.17% (-67.2% to -43.44%)	-62.98% (-71.64% to -51.11%)
Staphylococcus aureus	16283 (13286 to 20204)	0.94 (0.76 to 1.16)	7053 (4898 to 9779)	0.35 (0.24 to 0.49)	-56.68% (-66.91% to -42.19%)	-62.55% (-71.39% to -50.03%)
Haemophilus influenzae	32042 (26219 to 38505)	1.84 (1.51 to 2.21)	6659 (4654 to 9441)	0.33 (0.23 to 0.47)	-79.22% (-84.61% to -71.63%)	-82.03% (-86.7% to -75.48%)
Listeria monocytogenes	12480 (8930 to 16755)	0.72 (0.51 to 0.96)	5387 (3607 to 7576)	0.27 (0.18 to 0.38)	-56.83% (-67.46% to -42.01%)	-62.68% (-71.87% to -49.87%)

S9 Table. Incidence of childhood meningitis by age groups, 1990 and 2021, with EAPC from 1990 and 2021.

Age group	Incident cases in 1990	Rate in 1990 (per 100,000)	Incident cases in 2021	Rate in 2021 (per 100,000)	Changes of cases, 1990-2021	Changes of rates, 1990-2021	EAPC 1990-2021
0-14 years	2866040 (2297345 to 3486322)	164.8 (132.1 to 200.46)	1332734 (1107029 to 1577408)	66.24 (55.03 to 78.41)	-53.5% (-55.65 to -51.08)	-59.8% (-61.66 to -57.71)	-2.96% (-3.27 to -2.65)
0-6 days	89238 (68855 to 113864)	3514.62 (2711.85 to 4484.52)	50529 (40973 to 62597)	2061.4 (1671.53 to 2553.72)	-43.38% (-46.75 to -39.37)	-41.35% (-44.84 to -37.2)	-1.93% (-2.36 to -1.5)
7-27 days	140402 (108454 to 179214)	1871.89 (1445.95 to 2389.35)	64953 (53089 to 80529)	890.51 (727.85 to 1104.05)	-53.74% (-56.52 to -50.34)	-52.43% (-55.29 to -48.94)	-2.73% (-2.93 to -2.53)
<28 days	229639 (177308 to 293189)	2287.34 (1766.1 to 2920.33)	115483 (94084 to 143192)	1185.02 (965.44 to 1469.36)	-49.71% (-52.71 to -46.05)	-48.19% (-51.28 to -44.42)	-2.39% (-2.68 to -2.1)
1-5 months	558981 (442333 to 691518)	1023.86 (810.2 to 1266.62)	291013 (240044 to 349753)	541.35 (446.54 to 650.62)	-47.94% (-50.85 to -44.28)	-47.13% (-50.08 to -43.41)	-2.4% (-2.63 to -2.17)
6-11 months	397874 (293874 to 511311)	630.42 (465.64 to 810.16)	172121 (132557 to 216414)	272.38 (209.77 to 342.47)	-56.74% (-58.87 to -53.79)	-56.79% (-58.93 to -53.84)	-2.76% (-3.05 to -2.48)
2-4 years	695923 (488385 to 950737)	189.33 (132.87 to 258.65)	263385 (189045 to 352886)	65.35 (46.9 to 87.55)	-62.15% (-63.52 to -60.53)	-65.49% (-66.73 to -64.01)	-3.56% (-3.93 to -3.18)
<5 years	2322971 (1835721 to 2858705)	374.71 (296.11 to 461.13)	1015606 (838756 to 1220117)	154.31 (127.44 to 185.38)	-56.28% (-58.23 to -53.79)	-58.82% (-60.66 to -56.47)	-3.04% (-3.37 to -2.72)
5-9 years	356927 (243227 to 510614)	61.17 (41.68 to 87.5)	190952 (132650 to 266806)	27.79 (19.31 to 38.83)	-46.5% (-48.8 to -43.45)	-54.56% (-56.52 to -51.97)	-2.82% (-3.16 to -2.47)
10-14 years	186142 (115970 to 280704)	34.75 (21.65 to 52.4)	126176 (82781 to 181112)	18.93 (12.42 to 27.17)	-32.22% (-35.75 to -27.05)	-45.53% (-48.37 to -41.38)	-2.18% (-2.44 to -1.93)

S10 Table. Deaths of childhood meningitis by age group, 1990 and 2021, with EAPC from 1990 and 2021.

Age groups	Deaths in 1990	Rate in 1990 (per 100,000)	Deaths in 2021	Rate in 2021 (per 100,000)	Changes of deaths, 1990-2021	Changes of rates, 1990-2021	EAPC 1990-2021
0-14 years	315176 (270536 to 372951)	18.12 (15.56 to 21.44)	112373 (80908 to 154126)	5.59 (4.02 to 7.66)	-64.35% (-72.68 to -52.42)	-69.18% (-76.38 to -58.87)	-3.4% (-3.76 to -3.04)
0-6 days	12134 (10186 to 16870)	477.9 (401.17 to 664.44)	6849 (5261 to 9318)	279.42 (214.61 to 380.14)	-43.55% (-56.81 to -21.89)	-41.53% (-55.26 to -19.09)	-1.93% (-2.37 to -1.49)
7-27 days	18330 (15834 to 22378)	244.38 (211.11 to 298.36)	5790 (4475 to 7771)	79.38 (61.36 to 106.54)	-68.41% (-74.82 to -58.48)	-67.52% (-74.11 to -57.31)	-3.56% (-3.87 to -3.24)
<28 days	30464 (26319 to 39236)	303.44 (262.15 to 390.81)	12639 (9819 to 16643)	129.69 (100.76 to 170.78)	-58.51% (-67.16 to -44.71)	-57.26% (-66.17 to -43.04)	-2.8% (-3.16 to -2.44)
1-5 months	63620 (54615 to 75643)	116.53 (100.03 to 138.55)	20089 (15565 to 26300)	37.37 (28.95 to 48.92)	-68.42% (-75.51 to -57.19)	-67.93% (-75.13 to -56.52)	-3.51% (-3.88 to -3.14)
6-11 months	48484 (39208 to 60759)	76.82 (62.12 to 96.27)	19347 (12784 to 27271)	30.62 (20.23 to 43.16)	-60.1% (-70.54 to -45.01)	-60.15% (-70.58 to -45.08)	-2.7% (-3.01 to -2.38)
2-4 years	81432 (64096 to 102585)	22.15 (17.44 to 27.91)	25269 (14547 to 41477)	6.27 (3.61 to 10.29)	-68.97% (-79.85 to -45.56)	-71.7% (-81.62 to -50.36)	-3.9% (-4.3 to -3.49)
<5 years	272225 (230421 to 326873)	43.91 (37.17 to 52.73)	91147 (63151 to 129446)	13.85 (9.59 to 19.67)	-66.52% (-75.16 to -54.06)	-68.46% (-76.6 to -56.73)	-3.51% (-3.89 to -3.13)
5-9 years	27917 (23439 to 32483)	4.78 (4.02 to 5.57)	11744 (9332 to 15194)	1.71 (1.36 to 2.21)	-57.93% (-64.26 to -43.4)	-64.27% (-69.64 to -51.93)	-3.09% (-3.41 to -2.78)
10-14 years	15034 (13046 to 16823)	2.81 (2.44 to 3.14)	9481 (8026 to 11518)	1.42 (1.2 to 1.73)	-36.93% (-44.05 to -24.27)	-49.32% (-55.04 to -39.15)	-2.08% (-2.28 to -1.88)

S11 Table. Etiology proportions (%) of childhood meningitis deaths by age group, 2021.

Age group	Streptococcus pneumoniae	Neisseria meningitidis	Klebsiella pneumoniae	Viral etiologies	Other bacterial pathogen	Group B streptococcus	Escherichia coli	Staphylococcus aureus	Haemophilus influenzae	Listeria monocytogenes
0-14 years	17.01% (15.78% to 18.21%)	14.03% (12.97% to 15.03%)	12.11% (10% to 14.56%)	10.53% (9.94% to 11.1%)	10.27% (7.88% to 13.23%)	9.49% (8.56% to 10.43%)	9.45% (8.1% to 11.07%)	6.28% (5.6% to 7.08%)	5.92% (5.47% to 6.41%)	4.8% (3.84% to 6.08%)
<28 days	4.52% (3.66% to 5.53%)	8.63% (7.12% to 10.34%)	16.97% (13.48% to 21.23%)	15.35% (13.51% to 17.28%)	3.1% (1.97% to 4.51%)	22.7% (19.79% to 25.73%)	11.63% (9.18% to 14.46%)	5.66% (4.24% to 7.44%)	6.62% (5.51% to 8.05%)	4.68% (3.16% to 6.61%)
1-5 months	19.06% (17.64% to 20.46%)	14.5% (13.31% to 15.7%)	11.14% (8.71% to 14.2%)	8.81% (8.2% to 9.49%)	10.88% (7.75% to 14.9%)	8.31% (7.37% to 9.21%)	9.64% (7.92% to 11.72%)	6.2% (5.42% to 7.06%)	6.47% (5.96% to 7.05%)	4.87% (3.61% to 6.34%)
6-11 months	19.09% (17.65% to 20.58%)	14.29% (13.02% to 15.51%)	11.14% (8.65% to 14.21%)	8.88% (8.2% to 9.6%)	10.87% (7.64% to 14.94%)	8.28% (7.38% to 9.2%)	9.61% (7.87% to 11.73%)	6.21% (5.43% to 7.12%)	6.62% (6.01% to 7.25%)	4.85% (3.57% to 6.39%)
2-4 years	19.09% (17.59% to 20.63%)	13.49% (12.14% to 14.93%)	11.2% (8.68% to 14.35%)	9.01% (8.3% to 9.8%)	10.93% (7.67% to 15.02%)	8.34% (7.36% to 9.4%)	9.65% (7.9% to 11.77%)	6.28% (5.47% to 7.29%)	6.91% (6.24% to 7.64%)	4.86% (3.59% to 6.42%)
<5 years	17% (15.61% to 18.34%)	13.23% (12.17% to 14.35%)	12% (9.72% to 14.66%)	9.82% (9.2% to 10.45%)	9.82% (7.02% to 13.34%)	10.36% (9.33% to 11.39%)	9.94% (8.49% to 11.71%)	6.17% (5.44% to 7%)	6.68% (6.17% to 7.24%)	4.85% (3.74% to 6.23%)
5-9 years	17.04% (15.93% to 18.27%)	17.27% (15.85% to 18.61%)	12.6% (10.06% to 15.26%)	13.58% (12.73% to 14.45%)	12.19% (9.85% to 14.9%)	5.82% (5.26% to 6.47%)	7.41% (6.02% to 8.85%)	6.74% (5.97% to 7.55%)	2.68% (2.45% to 2.93%)	4.61% (3.75% to 5.62%)
10-14 years	17.04% (15.94% to 18.19%)	17.56% (16.28% to 18.84%)	12.54% (10.04% to 15.13%)	13.53% (12.7% to 14.33%)	12.14% (9.81% to 14.87%)	5.8% (5.25% to 6.43%)	7.38% (6.04% to 8.79%)	6.72% (5.97% to 7.51%)	2.66% (2.44% to 2.9%)	4.59% (3.75% to 5.61%)

S12 Table. Incidence of meningitis among neonates (0-27 days) in 1990 and 2021, with EAPC from 1990 to 2021.

	Incident cases in 1990	Rate in 1990 (per 100,000)	Incident cases in 2021	Rate in 2021 (per 100,000)	Changes of cases, 1990-2021	Changes of rates, 1990-2021	EAPC, 1990-2021
Global	229639 (177308 to 293189)	2287.34 (1766.1 to 2920.33)	115483 (94084 to 143192)	1185.02 (965.44 to 1469.36)	-49.71% (-52.71 to -46.05)	-48.19% (-51.28 to -44.42)	-2.39% (-2.68 to -2.1)
**Age**							
0-6 days	89238 (68855 to 113864)	3514.62 (2711.85 to 4484.52)	50529 (40973 to 62597)	2061.4 (1671.53 to 2553.72)	-43.38% (-46.75 to -39.37)	-41.35% (-44.84 to -37.2)	-1.93% (-2.36 to -1.5)
7-27 days	140402 (108454 to 179214)	1871.89 (1445.95 to 2389.35)	64953 (53089 to 80529)	890.51 (727.85 to 1104.05)	-53.74% (-56.52 to -50.34)	-52.43% (-55.29 to -48.94)	-2.73% (-2.93 to -2.53)
**Sex**							
Male	134813 (103859 to 172567)	2595.22 (1999.35 to 3322.01)	66364 (53906 to 81784)	1317.68 (1070.32 to 1623.86)	-50.77% (-53.87 to -46.97)	-49.23% (-52.42 to -45.31)	-2.52% (-2.82 to -2.22)
Female	94826 (73219 to 120459)	1957.24 (1511.25 to 2486.3)	49119 (39891 to 61082)	1043.13 (847.15 to 1297.18)	-48.2% (-51.24 to -44.37)	-46.7% (-49.83 to -42.77)	-2.21% (-2.49 to -1.92)
**5 SDI quintiles regions**							
High SDI	2079 (1622 to 2682)	218.7 (170.59 to 282.15)	428 (345 to 539)	54.59 (44.07 to 68.75)	-79.42% (-80.37 to -78.25)	-75.04% (-76.19 to -73.62)	-4.44% (-4.69 to -4.18)
High-middle SDI	9299 (7174 to 12021)	671.26 (517.87 to 867.74)	1139 (907 to 1455)	127.8 (101.76 to 163.21)	-87.75% (-88.46 to -86.82)	-80.96% (-82.07 to -79.51)	-5.74% (-5.93 to -5.55)
Middle SDI	33738 (25545 to 43928)	1071.53 (811.32 to 1395.17)	8779 (6969 to 11229)	362.88 (288.05 to 464.14)	-73.98% (-75.53 to -72.21)	-66.13% (-68.15 to -63.83)	-3.7% (-3.99 to -3.41)
Low-middle SDI	74001 (56955 to 95447)	2556.07 (1967.29 to 3296.85)	29731 (24031 to 37361)	1013.47 (819.17 to 1273.54)	-59.82% (-62.62 to -56.47)	-60.35% (-63.11 to -57.04)	-2.96% (-3.25 to -2.66)
Low SDI	110329 (85963 to 138881)	6678.89 (5203.86 to 8407.3)	75317 (61371 to 92436)	2780.22 (2265.41 to 3412.14)	-31.73% (-35.81 to -26.75)	-58.37% (-60.86 to -55.33)	-2.91% (-3.14 to -2.69)
**GBD regions**							
Andean Latin America	940 (743 to 1176)	1064.57 (840.71 to 1331.09)	154 (126 to 191)	164.29 (134.06 to 203.33)	-83.59% (-84.81 to -81.83)	-84.57% (-85.71 to -82.92)	-6.04% (-6.19 to -5.88)
Australasia	59 (48 to 74)	244.21 (197.74 to 306.75)	21 (17 to 25)	75.52 (62.6 to 93.24)	-65.09% (-68.09 to -61.29)	-69.08% (-71.73 to -65.7)	-4.47% (-4.9 to -4.03)
Caribbean	2880 (2283 to 3586)	4245.54 (3365.54 to 5285.85)	1216 (975 to 1482)	2026.28 (1623.72 to 2469.38)	-57.77% (-62.52 to -53.29)	-52.27% (-57.64 to -47.22)	-1.92% (-2.15 to -1.69)

	Incident cases in 1990	Rate in 1990 (per 100,000)	Incident cases in 2021	Rate in 2021 (per 100,000)	Changes of cases, 1990-2021	Changes of rates, 1990-2021	EAPC, 1990-2021
Central Asia	2241 (1878 to 2715)	1509.63 (1264.89 to 1828.89)	481 (396 to 598)	310.31 (255.18 to 385.83)	-78.53% (-80.05 to -76.9)	-79.44% (-80.9 to -77.89)	-5.8% (-6.25 to -5.35)
Central Europe	849 (686 to 1054)	651.24 (526.15 to 808.23)	69 (56 to 85)	86.23 (70.25 to 106.46)	-91.91% (-92.4 to -91.42)	-86.76% (-87.57 to -85.97)	-6.43% (-6.71 to -6.15)
Central Latin America	2852 (2274 to 3528)	756.62 (603.32 to 936.02)	466 (380 to 570)	157.19 (128.35 to 192.28)	-83.67% (-84.85 to -82.47)	-79.22% (-80.72 to -77.69)	-4.99% (-5.47 to -4.52)
Central Sub-Saharan Africa	13551 (10618 to 17142)	7049 (5523.27 to 8917.06)	7174 (5863 to 8859)	2136.17 (1745.74 to 2637.92)	-47.06% (-52.82 to -40.92)	-69.7% (-72.99 to -66.18)	-4.25% (-4.66 to -3.83)
East Asia	9715 (7147 to 12985)	537.19 (395.19 to 717.95)	564 (438 to 721)	64.39 (49.97 to 82.3)	-94.19% (-94.81 to -93.53)	-88.01% (-89.28 to -86.64)	-7.11% (-7.33 to -6.9)
Eastern Europe	2601 (1994 to 3430)	1145.53 (878.14 to 1510.89)	247 (194 to 317)	180.41 (142.26 to 232.15)	-90.52% (-91.19 to -89.57)	-84.25% (-85.38 to -82.68)	-6.39% (-6.65 to -6.13)
Eastern Sub-Saharan Africa	51547 (39968 to 65303)	7691.68 (5963.98 to 9744.39)	28006 (22988 to 34622)	2718.7 (2231.53 to 3360.92)	-45.67% (-49.69 to -40.69)	-64.65% (-67.27 to -61.42)	-3.48% (-3.74 to -3.23)
High-income Asia Pacific	259 (198 to 333)	172.15 (131.63 to 221.43)	30 (24 to 39)	33.29 (26.11 to 42.96)	-88.35% (-89.2 to -87.17)	-80.67% (-82.09 to -78.72)	-5.19% (-5.52 to -4.86)
High-income North America	491 (358 to 672)	141.35 (103.07 to 193.41)	136 (107 to 173)	44.05 (34.75 to 55.92)	-72.31% (-75.28 to -68.15)	-68.83% (-72.18 to -64.16)	-3.36% (-3.67 to -3.05)
North Africa and Middle East	12718 (9907 to 16027)	1545.28 (1203.68 to 1947.34)	3564 (2878 to 4372)	394.29 (318.4 to 483.69)	-71.97% (-74.13 to -69.19)	-74.48% (-76.45 to -71.95)	-4.91% (-5.38 to -4.43)
Oceania	262 (202 to 331)	1544.63 (1190.32 to 1955.45)	265 (206 to 329)	821.42 (636.48 to 1019.46)	1.35% (-10.26 to 14.72)	-46.82% (-52.91 to -39.81)	-3.33% (-3.77 to -2.9)
South Asia	49571 (37694 to 64931)	1933.76 (1470.45 to 2532.95)	19405 (15489 to 24380)	815.83 (651.19 to 1025)	-60.85% (-63.72 to -57.03)	-57.81% (-60.9 to -53.69)	-2.46% (-2.74 to -2.18)
Southeast Asia	13096 (9844 to 17253)	1407.98 (1058.37 to 1854.97)	3992 (3168 to 5076)	468.92 (372.19 to 596.31)	-69.52% (-71.66 to -67.05)	-66.7% (-69.04 to -63.99)	-3.64% (-3.81 to -3.47)
Southern Latin America	560 (476 to 674)	700.49 (595.74 to 843.32)	77 (65 to 89)	130.58 (110.16 to 150.57)	-86.27% (-87.43 to -84.98)	-81.36% (-82.94 to -79.61)	-5.44% (-5.63 to -5.24)
Southern Sub-Saharan Africa	1947 (1508 to 2498)	1597.65 (1237.88 to 2050.11)	961 (778 to 1187)	781.76 (632.7 to 965.14)	-50.63% (-54.71 to -44.63)	-51.07% (-55.11 to -45.12)	-2.52% (-2.76 to -2.27)
Tropical Latin America	2210 (1678 to 2833)	871.98 (661.99 to 1117.8)	315 (245 to 408)	120.64 (93.67 to 156.22)	-85.73% (-87.2 to -84.36)	-86.17% (-87.59 to -84.84)	-6.64% (-6.86 to -6.42)

	Incident cases in 1990	Rate in 1990 (per 100,000)	Incident cases in 2021	Rate in 2021 (per 100,000)	Changes of cases, 1990-2021	Changes of rates, 1990-2021	EAPC, 1990-2021
Western Europe	761 (611 to 946)	216.18 (173.47 to 268.86)	202 (164 to 250)	64.73 (52.64 to 79.91)	-73.44% (-74.38 to -72.35)	-70.06% (-71.11 to -68.83)	-3.97% (-4.17 to -3.77)
Western Sub-Saharan Africa	60529 (46739 to 76795)	9073.22 (7006.15 to 11511.39)	48137 (38999 to 59247)	3609.58 (2924.35 to 4442.7)	-20.47% (-25.49 to -14.29)	-60.22% (-62.73 to -57.12)	-3.13% (-3.41 to -2.85)

S13 Table. Incidence and death rate of meningitis among neonates in 204 countries, 1990 and 2021, with EAPC from 1990 to 2021.

Location	Incidence rate per 100,000 population			Death rate per 100,000 population		
	1990	2021	EAPC, 1990-2021	1990	2021	EAPC, 1990-2021
Afghanistan	10912.26 (8209.71 to 14045.11)	2202.74 (1719.46 to 2724.29)	-5.22% (-5.73% to -4.71%)	1496.17 (971.98 to 2260.18)	302.95 (176.49 to 510.48)	-5.22% (-5.72% to -4.71%)
Albania	748.6 (577.42 to 956.36)	143.26 (112.7 to 182.07)	-5.86% (-6.62% to -5.1%)	102.88 (69.97 to 151.84)	18.98 (10.36 to 32.39)	-5.84% (-6.59% to -5.09%)
Algeria	745.15 (563.18 to 953.54)	149.9 (117.81 to 193.37)	-5.01% (-5.36% to -4.67%)	102.31 (54.2 to 173.89)	14.68 (6.25 to 28.81)	-5.71% (-6.2% to -5.22%)
American Samoa	433.86 (339.84 to 575.29)	294.47 (229.28 to 381.62)	-1.44% (-1.54% to -1.33%)	39.95 (26.51 to 63.02)	23.91 (14.9 to 36.34)	-1.82% (-2.28% to -1.37%)
Andorra	72.95 (53.22 to 97.95)	24.03 (18.2 to 31.75)	-3.68% (-3.81% to -3.55%)	1.7 (0.96 to 2.6)	0.12 (0.06 to 0.2)	-7.31% (-7.82% to -6.79%)
Angola	9668.54 (7551.26 to 12325.47)	2542.96 (2079.97 to 3142.76)	-4.85% (-5.05% to -4.65%)	1327.81 (850.84 to 2068.23)	266.02 (164.63 to 423.42)	-5.55% (-5.84% to -5.27%)
Antigua and Barbuda	1016.43 (823.14 to 1261.52)	459.3 (366.99 to 581.51)	-3.19% (-3.46% to -2.92%)	138.83 (108.64 to 170.03)	30.37 (24.02 to 37.48)	-5.4% (-5.75% to -5.05%)
Argentina	818.23 (685.8 to 982.65)	139.27 (117.72 to 160.57)	-5.87% (-6.08% to -5.65%)	110.1 (101.29 to 119.09)	19.13 (14.52 to 24.74)	-5.84% (-6.06% to -5.62%)
Armenia	525.74 (408.55 to 675.33)	117.24 (87.8 to 155.84)	-5.37% (-5.73% to -5.01%)	68.86 (60.76 to 78.19)	4.76 (3.82 to 5.94)	-8.98% (-9.6% to -8.36%)
Australia	233.2 (189.46 to 289.07)	65.44 (54.35 to 79.72)	-4.56% (-4.96% to -4.16%)	32.03 (29.11 to 35.51)	7.5 (6.06 to 9.2)	-4.92% (-5.43% to -4.41%)
Austria	214.03 (176.55 to 262.45)	81.44 (68.13 to 97.63)	-3.09% (-3.44% to -2.74%)	29.38 (26.26 to 33.12)	5.76 (4.72 to 6.88)	-5.03% (-5.26% to -4.8%)
Azerbaijan	1224.34 (986.71 to 1491.36)	349.27 (286.5 to 433.42)	-4.55% (-5.2% to -3.89%)	168.21 (128.36 to 216.91)	40.08 (26.09 to 67.25)	-5.04% (-5.74% to -4.34%)
Bahamas	1017.93 (805.64 to 1278.9)	276.49 (218.48 to 354.67)	-4.28% (-4.47% to -4.09%)	139.84 (109.71 to 175.86)	20.95 (14.53 to 30.34)	-6.18% (-6.52% to -5.84%)
Bahrain	322.78 (255.79 to 414.93)	79.06 (62 to 100.83)	-4.93% (-5.16% to -4.69%)	35.67 (26.84 to 46)	3.46 (2.38 to 4.66)	-7.32% (-7.72% to -6.91%)
Bangladesh	1010.9 (793 to 1296.56)	421.75 (322.34 to 548.65)	-2.69% (-3.15% to -2.22%)	107.84 (38.56 to 196.55)	25.27 (2.17 to 56.47)	-3.76% (-4.4% to -3.11%)

Location	Incidence rate per 100,000 population			Death rate per 100,000 population		
	1990	2021	EAPC, 1990-2021	1990	2021	EAPC, 1990-2021
Barbados	1223.29 (999.76 to 1537.72)	398.55 (308.54 to 516.67)	-4.1% (-4.41% to -3.78%)	168.02 (139.53 to 201.92)	44.7 (30.1 to 62.41)	-4.79% (-5.21% to -4.36%)
Belarus	1009.35 (834.83 to 1229.45)	236.07 (190.22 to 294.19)	-5.36% (-5.73% to -4.99%)	131.98 (109.28 to 159.38)	25.41 (19.41 to 32.31)	-6.02% (-6.53% to -5.5%)
Belgium	173.72 (141.36 to 218.62)	70.5 (57.82 to 85.71)	-3.21% (-3.41% to -3.02%)	22.51 (20.16 to 25.11)	8.32 (6.59 to 10.18)	-3.63% (-4.03% to -3.23%)
Belize	1192.16 (992.14 to 1473.26)	291.36 (236.95 to 372.45)	-4.98% (-5.28% to -4.69%)	151.53 (130.92 to 177.71)	27.63 (22.22 to 33.83)	-5.53% (-5.93% to -5.12%)
Benin	7010.63 (5430.95 to 8885.13)	3081.86 (2482.84 to 3884.39)	-2.91% (-3.23% to -2.58%)	962.24 (655.46 to 1431.37)	266.4 (158.92 to 458.64)	-4.21% (-4.7% to -3.73%)
Bermuda	685.27 (530.01 to 882.11)	259.3 (196.58 to 332.85)	-3.29% (-3.53% to -3.04%)	42.65 (33.94 to 51.04)	5.18 (3.28 to 6.27)	-7.14% (-7.83% to -6.43%)
Bhutan	1989.96 (1506.67 to 2548.99)	642.42 (533 to 802.05)	-3.97% (-4.17% to -3.77%)	271.39 (163.18 to 458.85)	46.76 (26.55 to 81.17)	-5.85% (-6.2% to -5.49%)
Bolivia (Plurinational State of)	2076.86 (1561.92 to 2677.87)	318.13 (253.74 to 391.97)	-5.87% (-6.13% to -5.61%)	284.68 (178.15 to 513.25)	43.64 (25.3 to 75.33)	-5.87% (-6.13% to -5.61%)
Bosnia and Herzegovina	515.23 (409.06 to 632.73)	158.49 (128.29 to 196.86)	-5.04% (-5.78% to -4.3%)	61.93 (49.83 to 77.12)	16.68 (10.5 to 24.81)	-5.33% (-6.08% to -4.57%)
Botswana	1379.73 (1076.01 to 1769.57)	590.81 (485.18 to 744.85)	-2.91% (-3.37% to -2.45%)	176.73 (103.5 to 273.05)	61.75 (35.63 to 101.34)	-3.19% (-3.81% to -2.57%)
Brazil	867.24 (654.35 to 1117.23)	120.78 (93.54 to 157)	-6.64% (-6.86% to -6.41%)	118.84 (106.55 to 130.44)	7.88 (6.23 to 9.75)	-8.85% (-9.18% to -8.53%)
Brunei Darussalam	230.82 (180.69 to 291.01)	170.05 (138.53 to 208.98)	-0.9% (-1.17% to -0.63%)	31.65 (20.35 to 46.61)	23.32 (15.67 to 33.42)	-0.9% (-1.17% to -0.63%)
Bulgaria	608.76 (494.36 to 735.87)	115.11 (94.93 to 140.37)	-6.1% (-6.45% to -5.75%)	83.63 (74.14 to 94.34)	15.04 (12.05 to 18.23)	-6.18% (-6.53% to -5.82%)
Burkina Faso	12727.49 (9787.51 to 16127.58)	2897.08 (2250.68 to 3676.98)	-5.07% (-5.86% to -4.27%)	1748.42 (1211.01 to 2476.13)	392.95 (237.16 to 644.02)	-5.08% (-5.87% to -4.28%)
Burundi	6731.87 (5191.99 to 8413.93)	2071.77 (1668.96 to 2555.31)	-4.4% (-4.86% to -3.94%)	924.08 (565.91 to 1496.69)	232.14 (135.38 to 408.66)	-4.88% (-5.44% to -4.31%)
Cabo Verde	3778.75 (2759.74 to	970.95 (766.82 to	-4.88% (-5.6% to -	519.11 (248.82 to	64.75 (40.22 to	-6.61% (-7.5% to -

Location	Incidence rate per 100,000 population			Death rate per 100,000 population		
	1990	2021	EAPC, 1990-2021	1990	2021	EAPC, 1990-2021
	4984.8)	1243.43)	4.16%)	835.56)	99.13)	5.71%)
Cambodia	1013.2 (781.44 to 1306.16)	288.38 (230.47 to 361.22)	-4.57% (-5.05% to -4.09%)	138.83 (72.57 to 228.51)	31.19 (15.77 to 59.07)	-4.99% (-5.53% to -4.44%)
Cameroon	6527.11 (5133.12 to 8264.49)	2316.95 (1879.81 to 2947.07)	-3.37% (-3.66% to -3.08%)	895.15 (646.95 to 1242.36)	215.32 (143.2 to 342.33)	-4.63% (-5.02% to -4.25%)
Canada	116.95 (94.74 to 144.11)	68.34 (56.97 to 81.94)	-1.7% (-1.77% to -1.63%)	12.99 (11.42 to 14.45)	4.16 (3.39 to 5.14)	-3.12% (-3.42% to -2.82%)
Central African Republic	10045.71 (7886.78 to 12696.94)	3860.2 (3156.55 to 4742.12)	-2.89% (-3.25% to -2.52%)	1378.14 (998.78 to 2030.27)	469.18 (300.74 to 772.3)	-3.18% (-3.6% to -2.77%)
Chad	6717.35 (5146.54 to 8530.55)	4382.38 (3522.21 to 5371.22)	-1.73% (-2% to -1.46%)	921.92 (580.71 to 1504.05)	464.12 (295.42 to 755.52)	-2.6% (-2.92% to -2.27%)
Chile	444.85 (366.26 to 547.15)	110.66 (91.55 to 134.37)	-3.99% (-4.25% to -3.73%)	59.64 (53.85 to 66.31)	14.81 (12.28 to 17.78)	-3.93% (-4.21% to -3.65%)
China	545 (400.36 to 728.91)	62.3 (47.95 to 79.84)	-7.25% (-7.49% to -7.02%)	67.71 (45.42 to 92.9)	7.73 (5.44 to 9.94)	-7.03% (-7.48% to -6.58%)
Colombia	1240.17 (1003.74 to 1500.51)	132.41 (109.62 to 161.03)	-7.45% (-7.76% to -7.15%)	170.34 (148.22 to 196.49)	15.14 (10.49 to 20.69)	-7.65% (-8% to -7.3%)
Comoros	8139.3 (6396.25 to 10353.33)	3228.97 (2627.19 to 4020.56)	-3.05% (-3.25% to -2.85%)	1116.29 (761.19 to 1556.48)	363.63 (243.57 to 512.45)	-3.52% (-3.8% to -3.24%)
Congo	3896.21 (3022.97 to 4942.97)	1491.33 (1169.36 to 1892.67)	-3.5% (-4.01% to -3%)	499.07 (342.11 to 774.32)	158.22 (97 to 259.84)	-4.04% (-4.65% to -3.42%)
Cook Islands	242.83 (180.33 to 329.59)	126.59 (89.64 to 176.55)	-2.44% (-2.7% to -2.18%)	13 (8.74 to 19.49)	3.01 (1.81 to 4.8)	-7.23% (-8.18% to -6.27%)
Costa Rica	481.34 (386.79 to 587.17)	149.71 (123.52 to 182.74)	-4.09% (-4.34% to -3.85%)	66.12 (57.68 to 75.2)	18.46 (14.51 to 23.31)	-4.45% (-4.84% to -4.05%)
Coted'Ivoire	6908.65 (5244.66 to 8669.47)	2238.12 (1774.9 to 2798.44)	-3.83% (-4.31% to -3.34%)	947.97 (638.43 to 1369.49)	260.87 (166.03 to 421.87)	-4.08% (-4.64% to -3.52%)
Croatia	535.95 (434.1 to 666.33)	97.1 (75.06 to 123.26)	-4.93% (-5.43% to -4.43%)	73.62 (66.79 to 81.02)	13.12 (9.87 to 16.88)	-5.02% (-5.61% to -4.42%)
Cuba	1542.42 (1253.08 to 1972.65)	421.42 (328.49 to 529.74)	-4.2% (-4.56% to -3.84%)	166.16 (144.45 to 188.75)	17.72 (14.78 to 21.42)	-7.21% (-7.69% to -6.73%)

Location	Incidence rate per 100,000 population			Death rate per 100,000 population		
	1990	2021	EAPC, 1990-2021	1990	2021	EAPC, 1990-2021
Cyprus	240.45 (190.25 to 308.71)	25.56 (19.52 to 33.09)	-7.58% (-7.89% to -7.27%)	32.98 (21.21 to 49.84)	2.89 (1.89 to 4.24)	-8.04% (-8.25% to -7.83%)
Czechia	433.37 (358.28 to 531.48)	88.78 (70.08 to 113.49)	-5.09% (-5.7% to -4.48%)	59.53 (52.55 to 67.16)	7.1 (5.62 to 8.73)	-6.41% (-7% to -5.82%)
Democratic People's Republic of Korea	417.32 (319.32 to 539.36)	139.68 (113.22 to 173.22)	-3.7% (-4.02% to -3.38%)	40.45 (23.37 to 62.57)	12.36 (6.83 to 21.71)	-3.53% (-4.01% to -3.03%)
Democratic Republic of the Congo	6335.96 (4923.95 to 8101.09)	1884.23 (1513.59 to 2335.99)	-4.29% (-4.85% to -3.74%)	871.45 (501.62 to 1430.78)	195.7 (117.73 to 335.9)	-5.05% (-5.71% to -4.39%)
Denmark	406.41 (329.82 to 512.45)	123.85 (101.44 to 154.69)	-3.96% (-4.3% to -3.63%)	55.54 (48.81 to 61.53)	16.77 (13.57 to 20.26)	-3.96% (-4.3% to -3.62%)
Djibouti	4950.3 (3834.89 to 6206.48)	2104.54 (1692.53 to 2625.51)	-3.06% (-3.39% to -2.73%)	673.73 (427.5 to 978.12)	190.94 (123.86 to 298.9)	-4.35% (-4.78% to -3.91%)
Dominica	917.82 (708.66 to 1192.71)	621.1 (503.98 to 790.78)	-1.32% (-1.64% to -1%)	120.03 (83.78 to 164.48)	80.47 (44.83 to 133.66)	-1.39% (-1.86% to -0.92%)
Dominican Republic	2788.74 (2234.22 to 3496.32)	934.3 (734.63 to 1165.16)	-3.13% (-3.38% to -2.88%)	382.77 (268.24 to 546.96)	128.46 (77.75 to 207.1)	-3.12% (-3.37% to -2.87%)
Ecuador	834.86 (672.93 to 1031.42)	151.31 (124.89 to 184.43)	-5.56% (-5.97% to -5.14%)	114.68 (100.38 to 130.46)	13.54 (10.25 to 17.85)	-6.23% (-6.84% to -5.62%)
Egypt	1447.92 (1102.36 to 1906.64)	164.64 (134.05 to 208.95)	-7.47% (-7.81% to -7.13%)	198.92 (92.08 to 314.98)	18.31 (12.12 to 27.91)	-7.7% (-8.05% to -7.34%)
El Salvador	563.42 (442.59 to 712.28)	86.24 (69.67 to 106.94)	-6.33% (-6.51% to -6.15%)	77.37 (59.27 to 106.77)	9.1 (6.29 to 13.68)	-6.82% (-7% to -6.63%)
Equatorial Guinea	5634.5 (4311.17 to 7113.48)	2005.32 (1587.66 to 2527.1)	-3.75% (-3.9% to -3.6%)	773 (508.06 to 1169.98)	209.75 (122.53 to 349.53)	-4.71% (-4.94% to -4.47%)
Eritrea	5365.89 (4157.12 to 6795.72)	1755.01 (1380.75 to 2204.89)	-3.59% (-3.91% to -3.26%)	734.15 (443.86 to 1241.63)	188.39 (101.3 to 343.1)	-4.05% (-4.49% to -3.61%)
Estonia	908.61 (763.32 to 1108.53)	132.21 (105.15 to 173.19)	-6.63% (-6.94% to -6.31%)	119.22 (108.66 to 129.29)	12.05 (10.08 to 14.58)	-8.18% (-8.75% to -7.59%)
Eswatini	1295.14 (960.79 to 1651.05)	543.81 (449.89 to 650.89)	-2.53% (-2.81% to -2.25%)	177.78 (95.26 to 294.99)	58.3 (32.45 to 104.66)	-3.07% (-3.47% to -2.67%)
Ethiopia	9956.02	2937.09	-4.14%	1345.26	339.93	-4.5%

Location	Incidence rate per 100,000 population			Death rate per 100,000 population		
	1990	2021	EAPC, 1990-2021	1990	2021	EAPC, 1990-2021
	(7427.52 to 13045.16)	(2311.88 to 3744.6)	(-4.43% to -3.84%)	(1023.91 to 1816.27)	(249.73 to 452.96)	(-4.83% to -4.17%)
Fiji	717.73 (555.32 to 933.31)	273.01 (211.55 to 353.64)	-3.78% (-4.03% to -3.52%)	72.76 (40.85 to 123.43)	24.06 (12.85 to 40.39)	-3.92% (-4.52% to -3.32%)
Finland	196.18 (150.05 to 260.02)	55.59 (42.53 to 74.8)	-4.54% (-4.98% to -4.1%)	26.95 (23.93 to 30.44)	7.59 (6.19 to 9.05)	-4.61% (-5.09% to -4.12%)
France	253.93 (207.08 to 308.58)	77.84 (63.5 to 95.47)	-3.84% (-4.26% to -3.42%)	34.88 (32.14 to 38.32)	10.53 (8.22 to 13.01)	-3.93% (-4.4% to -3.44%)
Gabon	4549.7 (3550.43 to 5880)	1880.83 (1480.11 to 2386.08)	-2.53% (-2.81% to -2.24%)	592.09 (430.09 to 824.26)	201.86 (122.94 to 323.3)	-2.49% (-2.93% to -2.05%)
Gambia	5485.04 (4226.71 to 7023.92)	1955.16 (1528.32 to 2514.01)	-3.63% (-4% to -3.25%)	707.73 (503.83 to 924.18)	178.27 (108.25 to 304.77)	-4.48% (-4.98% to -3.98%)
Georgia	1005.07 (822.02 to 1265.37)	156.79 (130.91 to 188.79)	-7.01% (-7.59% to -6.44%)	117.9 (100.95 to 138.4)	7.13 (5.47 to 9.25)	-9.47% (-10.32% to -8.62%)
Germany	135.93 (110.57 to 171.75)	35.91 (28.4 to 45.67)	-3.87% (-4.11% to -3.64%)	18.67 (16.68 to 20.87)	4.81 (4.08 to 5.57)	-3.92% (-4.17% to -3.67%)
Ghana	6573.06 (5060.31 to 8484.77)	1962.53 (1591.54 to 2470.79)	-4.34% (-4.72% to -3.95%)	902.4 (591.58 to 1401.61)	202.18 (113.24 to 341.89)	-4.86% (-5.35% to -4.36%)
Greece	223.36 (182.4 to 278.16)	64.82 (52.18 to 79.63)	-4.17% (-4.32% to -4.03%)	29.24 (25.21 to 34.33)	8.13 (6.47 to 10.2)	-4.5% (-5.13% to -3.86%)
Greenland	747.31 (579.32 to 964.1)	147.05 (116.17 to 180.1)	-5.11% (-5.37% to -4.85%)	102.56 (64.79 to 167.14)	18.85 (13.1 to 27.82)	-5.16% (-5.43% to -4.9%)
Grenada	961.81 (779.75 to 1191.33)	254.57 (198.29 to 321.47)	-4.39% (-4.65% to -4.14%)	98.77 (80.81 to 122)	12.36 (9.64 to 15.7)	-6.03% (-6.63% to -5.42%)
Guam	405.87 (306.99 to 527.51)	348.93 (278.56 to 441.31)	-0.39% (-0.51% to -0.26%)	31.63 (20.79 to 42.76)	24.01 (17.27 to 31.9)	-0.56% (-0.92% to -0.2%)
Guatemala	673.77 (550.33 to 818.7)	163.4 (134.38 to 190.78)	-4.57% (-5.13% to -4.01%)	92.54 (82.05 to 104.47)	22.45 (16.52 to 29.53)	-4.57% (-5.13% to -4.01%)
Guinea	11633.28 (8920.28 to 14779.72)	5269.26 (4292.28 to 6444.14)	-2.9% (-3.16% to -2.64%)	1597.35 (1054.63 to 2331.25)	599.99 (375.72 to 935.27)	-3.24% (-3.52% to -2.95%)
Guinea-Bissau	9854.17 (7709.72 to 12242.95)	2880.26 (2341.46 to 3605.85)	-4.42% (-5.09% to -3.74%)	1353.14 (899.87 to 1966.45)	300.43 (171.36 to 508.66)	-5.04% (-5.8% to -4.26%)

Location	Incidence rate per 100,000 population			Death rate per 100,000 population		
	1990	2021	EAPC, 1990-2021	1990	2021	EAPC, 1990-2021
Guyana	1364.92 (1114.64 to 1687.22)	268.27 (217.09 to 336.86)	-5.56% (-5.75% to -5.36%)	178.26 (145.15 to 219.76)	27.12 (17.81 to 38.4)	-6.17% (-6.7% to -5.64%)
Haiti	10134.96 (7874.53 to 12776.06)	3742.35 (2947.87 to 4592.13)	-2.64% (-2.9% to -2.38%)	1392.17 (840.83 to 2337.65)	513.68 (305.78 to 892.31)	-2.64% (-2.9% to -2.38%)
Honduras	698.82 (533.29 to 899.2)	531.43 (417.01 to 668.18)	0.42% (-0.47% to 1.32%)	95.92 (66.22 to 138.36)	73.28 (14.19 to 134.15)	0.43% (-0.46% to 1.33%)
Hungary	736.3 (602.47 to 892.98)	99.54 (80.77 to 126.01)	-6.28% (-6.71% to -5.85%)	101.15 (89.95 to 112.55)	13.68 (10.45 to 17.1)	-6.28% (-6.71% to -5.85%)
Iceland	227.96 (178.54 to 288.99)	48.06 (37.39 to 61.08)	-5.12% (-5.32% to -4.93%)	31.31 (27.23 to 35.33)	6.57 (5.08 to 8.05)	-5.12% (-5.32% to -4.93%)
India	1504.62 (1147.66 to 1968.34)	659.26 (524.62 to 843.92)	-2.03% (-2.45% to -1.62%)	182.21 (142.31 to 260.68)	68.89 (45.92 to 98.28)	-1.96% (-2.51% to -1.4%)
Indonesia	2217.73 (1594.88 to 2999.69)	741.33 (567.77 to 962.49)	-3.53% (-3.68% to -3.37%)	283.24 (216.73 to 372.34)	96.4 (64.9 to 131.82)	-3.39% (-3.56% to -3.22%)
Iran (Islamic Republic of)	727.58 (546.71 to 956.7)	101.19 (78.82 to 130.89)	-6.07% (-6.55% to -5.58%)	92.83 (72.31 to 135.23)	6.86 (4.95 to 9.51)	-6.68% (-7.5% to -5.86%)
Iraq	1322.07 (1015.2 to 1692.1)	320.12 (256.22 to 398.81)	-4.46% (-4.89% to -4.02%)	181.47 (111.61 to 277.68)	41.13 (25.4 to 62.24)	-4.53% (-4.98% to -4.07%)
Ireland	223.66 (178 to 280.13)	64.01 (51.46 to 79.11)	-4.49% (-4.72% to -4.25%)	26.85 (23.41 to 30.46)	7.28 (5.85 to 8.92)	-4.58% (-4.99% to -4.16%)
Israel	285.78 (225.98 to 356.08)	44.88 (35.75 to 55.61)	-5.29% (-5.59% to -4.99%)	39.25 (35.22 to 42.71)	6.17 (4.94 to 7.52)	-5.29% (-5.59% to -4.99%)
Italy	123.21 (87.32 to 169.92)	40.02 (30.51 to 51.64)	-3.67% (-3.95% to -3.4%)	14.88 (14.01 to 15.66)	5.32 (4.23 to 6.41)	-3.39% (-3.62% to -3.15%)
Jamaica	1313.57 (1088.76 to 1630.38)	409.85 (332.54 to 516.47)	-4.33% (-4.69% to -3.96%)	180.46 (150.81 to 212.84)	43.08 (29.65 to 58.36)	-5.03% (-5.45% to -4.6%)
Japan	109.01 (77.59 to 150.54)	31.67 (24.04 to 41.76)	-3.85% (-4.21% to -3.49%)	14.9 (14.07 to 15.7)	2.73 (2.28 to 3.21)	-5.15% (-5.6% to -4.69%)
Jordan	621.17 (475.31 to 798.31)	196.04 (151.13 to 253.18)	-4.2% (-4.51% to -3.89%)	85.33 (58.2 to 124.28)	20.25 (15.52 to 26.56)	-4.97% (-5.23% to -4.71%)
Kazakhstan	1094.17 (919.56 to	189.88 (152.87 to	-6.93% (-7.55% to -	148.49 (123.17 to	7.8 (6.11 to	-10.53% (-11.57%

Location	Incidence rate per 100,000 population			Death rate per 100,000 population		
	1990	2021	EAPC, 1990-2021	1990	2021	EAPC, 1990-2021
	1339.58)	236.92)	6.31%)	177.37)	9.84)	to -9.48%)
Kenya	5138.94 (3838.35 to 6693.51)	1653.5 (1304.56 to 2126.16)	-3.72% (-4.21% to -3.21%)	698.58 (542.3 to 993.91)	196.36 (134.27 to 297.94)	-3.93% (-4.49% to -3.36%)
Kiribati	2625.77 (2017.09 to 3351.61)	1003.66 (762.85 to 1260.35)	-3.31% (-3.55% to -3.07%)	360.65 (256.4 to 535.41)	131.66 (85.72 to 219.53)	-3.43% (-3.69% to -3.17%)
Kuwait	326.4 (257.29 to 414.19)	141.85 (111.92 to 183.42)	-2.45% (-2.66% to -2.24%)	30.03 (25.53 to 35.2)	10.5 (8.29 to 13.3)	-2.14% (-2.8% to -1.47%)
Kyrgyzstan	2555.6 (2130.98 to 3147.68)	233.49 (190.94 to 285.15)	-8.34% (-8.69% to -7.99%)	340.6 (289.94 to 401.2)	15.93 (12.79 to 19.94)	-10.36% (-10.92% to -9.8%)
Lao People's Democratic Republic	1429.29 (1095.72 to 1788.46)	410.89 (332.7 to 497.48)	-4.62% (-4.9% to -4.35%)	133.96 (67.26 to 245.59)	35.77 (19.53 to 65.62)	-5.17% (-5.75% to -4.6%)
Latvia	1041.68 (867.28 to 1272.48)	183.47 (144.25 to 231.44)	-7.3% (-7.94% to -6.66%)	143.08 (129.75 to 155.96)	21.24 (17.71 to 24.93)	-7.58% (-8.16% to -6.99%)
Lebanon	552.66 (429.42 to 721.92)	102.33 (77.42 to 135.58)	-5.7% (-5.9% to -5.51%)	74.13 (44.53 to 112.4)	9.49 (5.65 to 15.08)	-6.59% (-6.87% to -6.3%)
Lesotho	1642.07 (1289.42 to 2125.54)	848.71 (698.27 to 1020.06)	-2.13% (-2.48% to -1.79%)	220.17 (129.23 to 353.95)	100.69 (56.74 to 168.44)	-2.31% (-2.76% to -1.87%)
Liberia	9850.11 (7685.74 to 12704.16)	1909.32 (1509.96 to 2379.82)	-5.29% (-5.55% to -5.04%)	1352.03 (913.11 to 2044.81)	222.07 (125.53 to 391.44)	-5.57% (-5.9% to -5.24%)
Libya	475.41 (369.04 to 599.23)	105.44 (83.12 to 134.2)	-4.88% (-5.1% to -4.66%)	65.14 (33.2 to 121.33)	14.08 (6.75 to 26.44)	-4.92% (-5.24% to -4.59%)
Lithuania	801.86 (658.67 to 988.79)	155.83 (124.81 to 193.24)	-5.52% (-5.88% to -5.15%)	96.12 (87.13 to 106.47)	21.41 (17.98 to 25.24)	-5.45% (-6.06% to -4.82%)
Luxembourg	178.66 (143.1 to 224.15)	45.45 (35.31 to 58.37)	-4.37% (-4.9% to -3.83%)	24.54 (21.64 to 28.45)	6.24 (5.01 to 7.64)	-4.43% (-5.19% to -3.67%)
Madagascar	6307.5 (4936.06 to 7981.51)	2590.19 (2084.15 to 3207.17)	-3.02% (-3.42% to -2.63%)	865.42 (553.55 to 1281.78)	322.88 (194.58 to 491.52)	-3.17% (-3.6% to -2.74%)
Malawi	11984.15 (9318.46 to 15102.58)	3118.18 (2541.17 to 3848.05)	-4.33% (-4.55% to -4.1%)	1645.59 (995.75 to 2491.46)	367.15 (230.83 to 625.76)	-4.73% (-4.93% to -4.53%)
Malaysia	482 (368.54 to 631.28)	232.94 (179.61 to 299.94)	-2.22% (-2.56% to -1.88%)	29.66 (18.9 to 43.33)	9.15 (5.9 to 13.24)	-2.09% (-2.87% to -1.3%)

Location	Incidence rate per 100,000 population			Death rate per 100,000 population		
	1990	2021	EAPC, 1990-2021	1990	2021	EAPC, 1990-2021
Maldives	382.89 (295.77 to 499.04)	141.84 (110.52 to 183.25)	-3.47% (-3.67% to -3.28%)	30.33 (17.99 to 57.47)	6.68 (4.39 to 9.88)	-4.66% (-5.11% to -4.21%)
Mali	16783.45 (12894.53 to 22036.87)	3241.14 (2581.44 to 4081.92)	-5.68% (-5.86% to -5.5%)	2299.38 (1179.85 to 3742.84)	331.7 (210.16 to 492.68)	-6.47% (-6.75% to -6.19%)
Malta	305.45 (244.25 to 377.69)	80.4 (65.7 to 98.38)	-3.89% (-4.23% to -3.56%)	41.96 (36.32 to 49.11)	10.67 (8.17 to 13.6)	-3.92% (-4.26% to -3.58%)
Marshall Islands	664.33 (507.35 to 864.49)	261.38 (205.11 to 336.97)	-3.36% (-3.59% to -3.14%)	65.02 (37.94 to 107.95)	22.37 (11.23 to 41.56)	-3.6% (-3.88% to -3.33%)
Mauritania	5288.84 (4080.75 to 6807.57)	1572.33 (1248.95 to 1989.03)	-4.09% (-4.6% to -3.58%)	726.15 (468.95 to 1052.65)	183.05 (119.78 to 283.8)	-4.44% (-5.05% to -3.84%)
Mauritius	571.55 (474.9 to 706.75)	219.62 (178.56 to 283.98)	-3.18% (-3.41% to -2.94%)	20.48 (18.1 to 23.26)	16.55 (12.6 to 21.12)	6.76% (4.3% to 9.28%)
Mexico	506.96 (367.38 to 681.63)	57.59 (44.45 to 73.97)	-6.96% (-7.62% to -6.3%)	69.01 (62.78 to 76.27)	7.91 (6.39 to 9.59)	-6.95% (-7.61% to -6.29%)
Micronesia (Federated States of)	728.57 (563.91 to 937.23)	199.91 (154.7 to 255.92)	-4.67% (-4.84% to -4.49%)	73.26 (43.14 to 124.17)	17.38 (8.64 to 32.75)	-4.99% (-5.18% to -4.8%)
Monaco	129.97 (95.22 to 174.82)	40.95 (31.44 to 54.13)	-4.03% (-4.36% to -3.7%)	14.48 (7.75 to 25.21)	4.01 (2.14 to 6.53)	-4.63% (-5.05% to -4.21%)
Mongolia	2028.78 (1562.65 to 2593.28)	250.97 (189.45 to 336.13)	-7.49% (-7.86% to -7.12%)	266.59 (120.54 to 462.4)	34.44 (20.79 to 52.46)	-7.34% (-7.72% to -6.96%)
Montenegro	229.17 (181.85 to 289.7)	68.53 (52.19 to 88.29)	-4.55% (-4.86% to -4.24%)	29.88 (21 to 42.62)	3.99 (2.7 to 6.08)	-7.13% (-7.76% to -6.5%)
Morocco	1553.73 (1192.13 to 1973.65)	247.03 (189.47 to 317.03)	-6.25% (-6.69% to -5.81%)	213.12 (110.35 to 390.42)	23.51 (10.51 to 49.29)	-7.21% (-7.74% to -6.68%)
Mozambique	5751.64 (4476.56 to 7217.56)	2140.63 (1721.96 to 2729.79)	-3.41% (-3.92% to -2.9%)	732.9 (441.74 to 1060.79)	140.45 (69.95 to 265.99)	-5.45% (-6.07% to -4.81%)
Myanmar	2129.98 (1546.48 to 2830)	610.7 (487.34 to 747.24)	-4.1% (-4.5% to -3.69%)	292.45 (166.42 to 551.53)	68.95 (41.54 to 113.66)	-4.38% (-4.88% to -3.88%)
Namibia	1322.24 (1028.3 to 1707.71)	516.02 (417.86 to 654.26)	-3.67% (-4.65% to -2.67%)	171.01 (95.43 to 279.76)	49.5 (27.55 to 84.62)	-4.34% (-5.41% to -3.25%)
Nauru	591.95 (444.86 to	379.39 (299.02 to	-1.79% (-2.13% to -	53.5 (30.61 to	32.01 (15.93 to	-2.03% (-2.7% to -

Location	Incidence rate per 100,000 population			Death rate per 100,000 population		
	1990	2021	EAPC, 1990-2021	1990	2021	EAPC, 1990-2021
	771.63)	478.36)	1.44%)	84.76)	51.15)	1.34%)
Nepal	1551.1 (1155.24 to 2066.01)	472.84 (370.71 to 605.54)	-3.31% (-3.66% to -2.95%)	203.1 (121.1 to 347.35)	60.11 (28.62 to 106.28)	-3.2% (-3.58% to -2.82%)
Netherlands	256.85 (205.9 to 311.67)	112.17 (89.15 to 137.88)	-3.07% (-3.36% to -2.77%)	35.28 (30.89 to 39.86)	15.41 (12.69 to 18.51)	-3.07% (-3.36% to -2.77%)
New Zealand	291.15 (209.5 to 403.94)	124.06 (94.76 to 162.7)	-4% (-4.62% to -3.38%)	34.92 (31.12 to 38.71)	16.97 (14.44 to 19.6)	-3.97% (-4.76% to -3.18%)
Nicaragua	1218.3 (946.18 to 1565.14)	212.85 (168.9 to 258.41)	-6.04% (-6.35% to -5.73%)	167.39 (123.96 to 231.5)	29.17 (17.93 to 45.39)	-6.05% (-6.35% to -5.73%)
Niger	11980.13 (9402.15 to 15416.83)	3496.61 (2776.19 to 4357.37)	-5.27% (-5.75% to -4.79%)	1643.8 (1006.83 to 2354.42)	432.38 (263.2 to 678.79)	-5.43% (-5.91% to -4.94%)
Nigeria	9024.46 (6827.42 to 11756.53)	4387.73 (3517.21 to 5522.49)	-2.18% (-2.53% to -1.84%)	1218.82 (937.12 to 1764.08)	472.16 (342.9 to 669.16)	-2.61% (-3.07% to -2.14%)
Niue	526.58 (405.57 to 679.64)	394.96 (309.68 to 507.76)	-2.97% (-3.62% to -2.32%)	46.46 (28.46 to 68.5)	54.16 (28.12 to 95.57)	-2.76% (-3.82% to -1.68%)
North Macedonia	878.79 (698.46 to 1100.23)	107.24 (87.02 to 132.47)	-5.91% (-6.34% to -5.48%)	120.64 (96.41 to 149.86)	11.62 (8.6 to 16.3)	-6.15% (-6.66% to -5.64%)
Northern Mariana Islands	281.38 (209.31 to 379.36)	242.7 (182.24 to 322.54)	-0.39% (-0.56% to -0.23%)	14.12 (8.64 to 20.83)	11.1 (6.72 to 18.21)	-0.01% (-0.47% to 0.45%)
Norway	304.04 (223.58 to 420.45)	71.78 (53.3 to 96.58)	-5.13% (-5.71% to -4.55%)	41.7 (38.37 to 45.61)	7.51 (6.37 to 8.61)	-5.39% (-6% to -4.77%)
Oman	419.52 (311 to 547.52)	108.63 (82.94 to 142.74)	-4.97% (-5.4% to -4.53%)	49.66 (30 to 81.52)	6.99 (4.45 to 10.39)	-6.2% (-6.97% to -5.43%)
Pakistan	5504.24 (4124.57 to 7247.9)	1583.82 (1265.8 to 1965.35)	-4.28% (-4.49% to -4.07%)	753.24 (518.6 to 1091.01)	171.57 (108.38 to 301.08)	-5.01% (-5.32% to -4.7%)
Palau	642.56 (494.26 to 841.73)	184.72 (141.72 to 240.54)	-4.25% (-4.42% to -4.09%)	63.22 (37.96 to 93.69)	17.4 (9.56 to 28.51)	-4.22% (-4.44% to -3.99%)
Palestine	627.75 (479.73 to 811.8)	122.08 (97.04 to 154.4)	-5.44% (-5.9% to -4.97%)	81.62 (49.92 to 135.36)	12.34 (7.28 to 20.46)	-5.99% (-6.52% to -5.46%)
Panama	900.81 (712.48 to 1115.62)	216.79 (174.45 to 262.68)	-4.8% (-5.2% to -4.39%)	123.76 (102.9 to 144.63)	29.79 (22.29 to 38.88)	-4.8% (-5.2% to -4.39%)
Papua New	1855.65	915.48	-3.72%	106.25	47.45	-5.97%

Location	Incidence rate per 100,000 population			Death rate per 100,000 population		
	1990	2021	EAPC, 1990-2021	1990	2021	EAPC, 1990-2021
Guinea	(1427.41 to 2351.53)	(704.49 to 1130.19)	(-4.22% to -3.22%)	(65.44 to 160.76)	(21.7 to 92.13)	(-7.4% to -4.51%)
Paraguay	990.31 (783.76 to 1233.55)	116.96 (94.26 to 144.28)	-6.73% (-6.91% to -6.55%)	133.76 (77.93 to 191)	11.26 (7.14 to 16.99)	-7.85% (-8.08% to -7.62%)
Peru	814.98 (646.54 to 1022.15)	114.91 (90.28 to 146.69)	-6.6% (-6.83% to -6.37%)	111.72 (80.38 to 152.34)	10.14 (5.68 to 16.36)	-7.66% (-7.92% to -7.41%)
Philippines	491.59 (369 to 644.1)	196.49 (156.48 to 252.48)	-3.12% (-3.42% to -2.81%)	50.7 (39.64 to 69.2)	21.93 (16.15 to 29.43)	-2.47% (-2.87% to -2.08%)
Poland	621.35 (438.54 to 845.36)	57.47 (43.46 to 74.12)	-6.48% (-7.31% to -5.65%)	85.35 (80.26 to 90.38)	7.9 (6.01 to 10.65)	-6.4% (-7.32% to -5.47%)
Portugal	349.03 (282.75 to 432.11)	57.58 (45.85 to 71.86)	-5.64% (-6.2% to -5.08%)	47.94 (42.9 to 53.88)	7.91 (6.37 to 10.11)	-5.64% (-6.2% to -5.08%)
Puerto Rico	649.87 (518.34 to 818.41)	225.7 (177.35 to 291.07)	-3.57% (-3.75% to -3.38%)	68.33 (60.55 to 76.19)	4.39 (3.53 to 5.38)	-8.3% (-9.1% to -7.49%)
Qatar	264.12 (192.3 to 346.92)	94.66 (70.49 to 122.61)	-3.63% (-3.9% to -3.37%)	26.43 (14.93 to 47.55)	4 (2.57 to 5.97)	-5.88% (-6.18% to -5.57%)
Republic of Korea	282.44 (218.85 to 362.79)	35.26 (27.35 to 44)	-6.6% (-6.88% to -6.31%)	38.75 (27.37 to 50.65)	3.14 (2.02 to 4.64)	-7.14% (-7.4% to -6.89%)
Republic of Moldova	1980.83 (1690.03 to 2382.44)	513.56 (425.77 to 623.75)	-3.57% (-3.89% to -3.24%)	272.09 (242.6 to 308.51)	70.57 (51.21 to 96.5)	-3.44% (-3.82% to -3.07%)
Romania	961.21 (788.45 to 1185.03)	110.78 (89.47 to 136.37)	-6.79% (-7.49% to -6.09%)	132.01 (116.73 to 147.25)	13.17 (10.7 to 17.38)	-6.98% (-7.69% to -6.25%)
Russian Federation	1087.55 (826.95 to 1446.54)	158.46 (122.57 to 205.33)	-6.58% (-6.9% to -6.27%)	145.32 (140.08 to 151.14)	18.73 (16.3 to 21.58)	-6.85% (-7.18% to -6.53%)
Rwanda	7604.86 (5828.52 to 9632.8)	1794.65 (1453.04 to 2248.08)	-5.23% (-5.51% to -4.94%)	1044.52 (646.1 to 1692.29)	191.01 (102.97 to 323.95)	-5.9% (-6.27% to -5.52%)
Saint Kitts and Nevis	1587.36 (1304.14 to 1931.16)	408.6 (325.5 to 512.01)	-4.77% (-5.25% to -4.29%)	204.46 (180.4 to 232.01)	48.26 (37.08 to 61.46)	-5.03% (-5.56% to -4.5%)
Saint Lucia	1179.05 (967.5 to 1481.2)	406.58 (321.42 to 518.27)	-3.8% (-4.02% to -3.57%)	150.31 (124.89 to 179.74)	38.02 (27.02 to 52.61)	-4.86% (-5.19% to -4.54%)
Saint Vincent and the Grenadines	1510.78 (1239.97 to 1894.44)	438.75 (348.83 to 543.15)	-4.99% (-5.38% to -4.61%)	190.06 (154.8 to 233.77)	37.24 (27.34 to 49.93)	-6.08% (-6.55% to -5.61%)

Location	Incidence rate per 100,000 population			Death rate per 100,000 population		
	1990	2021	EAPC, 1990-2021	1990	2021	EAPC, 1990-2021
Samoa	639.86 (499.07 to 831.51)	271.98 (214.22 to 358.75)	-2.85% (-2.95% to -2.76%)	58.54 (34.56 to 92.66)	20.07 (11.03 to 33.23)	-3.41% (-3.6% to -3.22%)
San Marino	117.38 (85.31 to 156.85)	34.68 (27.3 to 45.77)	-3.93% (-4.18% to -3.68%)	11.93 (7.26 to 19.69)	0.92 (0.41 to 1.75)	-6.78% (-7.17% to -6.38%)
Sao Tome and Principe	2568.98 (1994.85 to 3358.92)	497.97 (375.81 to 643.81)	-5.86% (-6.6% to -5.1%)	344.19 (208.84 to 548.24)	56.21 (31.77 to 99.58)	-6.16% (-7% to -5.32%)
Saudi Arabia	425.44 (328.54 to 562.93)	43.07 (31.43 to 59.73)	-8.31% (-8.88% to -7.73%)	58.42 (35.98 to 89.25)	3.03 (1.87 to 5.14)	-9.84% (-10.49% to -9.18%)
Senegal	6769.9 (5199.61 to 8540.64)	2101.36 (1690.16 to 2631.19)	-4.29% (-4.77% to -3.8%)	929.49 (645.02 to 1246.32)	224.18 (132.31 to 379.11)	-4.74% (-5.34% to -4.14%)
Serbia	475.3 (374.61 to 599.94)	72.2 (57.43 to 90.38)	-6.78% (-7.32% to -6.24%)	65.2 (50.69 to 84.28)	7.25 (5.26 to 10.1)	-7.18% (-7.8% to -6.54%)
Seychelles	2536.15 (2113.52 to 3027.66)	288.34 (226.51 to 368.5)	-6.64% (-8.25% to -5.01%)	348.32 (318.74 to 381.32)	32.94 (22.15 to 49.32)	-5.64% (-8.03% to -3.18%)
Sierra Leone	9156.24 (7118.05 to 11657.74)	2768.37 (2154.81 to 3411.35)	-4.47% (-4.72% to -4.23%)	1257.55 (871.97 to 1933.92)	236.71 (159.5 to 349.31)	-5.99% (-6.43% to -5.54%)
Singapore	258.19 (204.31 to 322.7)	33.76 (26.66 to 41.28)	-5.99% (-6.76% to -5.21%)	35.47 (31.26 to 39.73)	4.64 (3.55 to 5.84)	-6.01% (-6.78% to -5.23%)
Slovakia	405.7 (330.44 to 505.68)	76.29 (61.56 to 94.84)	-6.35% (-6.75% to -5.94%)	55.75 (43.05 to 71.08)	8.54 (5.87 to 12.29)	-6.64% (-7% to -6.28%)
Slovenia	289.9 (227.25 to 364.21)	63.71 (48.22 to 81.37)	-4.3% (-4.63% to -3.98%)	39.82 (35.47 to 44.31)	5.76 (4.7 to 7.06)	-5.41% (-5.88% to -4.94%)
Solomon Islands	718.73 (552.85 to 925.58)	259.88 (205.44 to 324.75)	-3.32% (-3.74% to -2.9%)	80.91 (45.3 to 128.19)	24.5 (13.44 to 38.69)	-3.42% (-4.01% to -2.82%)
Somalia	8013.52 (6142.06 to 10129.39)	4876.38 (3984.51 to 5989.39)	-1.48% (-1.65% to -1.3%)	1098.62 (628.91 to 1906.08)	666.95 (395.44 to 1512.67)	-1.47% (-1.64% to -1.3%)
South Africa	1457.77 (1098.99 to 1902.29)	592.84 (467.18 to 767.24)	-3.33% (-3.5% to -3.17%)	198.38 (151.74 to 267.42)	69.83 (51.78 to 92.02)	-3.67% (-3.82% to -3.52%)
South Sudan	8029.41 (6079.01 to 10185.33)	6786.3 (5449.79 to 8543.01)	-0.42% (-0.49% to -0.34%)	1016.46 (661.17 to 1465.86)	717.41 (439.05 to 1129.55)	-0.89% (-1.03% to -0.75%)
Spain	319.6 (254.9 to	54.97 (44.42 to	-4.91% (-5.15% to -	43.9 (39.19 to	7.56 (5.58 to	-4.91% (-5.14% to

Location	Incidence rate per 100,000 population			Death rate per 100,000 population		
	1990	2021	EAPC, 1990-2021	1990	2021	EAPC, 1990-2021
	398.97)	67.62)	4.67%)	48.55)	11.1)	-4.67%)
Sri Lanka	575.27 (448.64 to 743.1)	275.86 (211.39 to 349.43)	-2.71% (-2.9% to -2.52%)	48.55 (36.08 to 68.58)	23.13 (13.96 to 33.79)	-2.05% (-2.54% to -1.55%)
Sudan	2359.94 (1777.82 to 3018.73)	336.73 (273.02 to 422.97)	-5.55% (-6.47% to -4.61%)	323.95 (233.32 to 464.89)	32.97 (16 to 61.46)	-6.26% (-7.37% to -5.13%)
Suriname	2398.32 (1883.25 to 2968.42)	658.52 (543.41 to 808.76)	-4.52% (-4.83% to -4.2%)	329.36 (230.86 to 463.94)	84.01 (45.44 to 135.48)	-4.79% (-5.16% to -4.42%)
Sweden	104.41 (76.09 to 143.44)	50.88 (38.13 to 68.83)	-3.08% (-3.82% to -2.33%)	13.68 (12.15 to 15.44)	6.91 (5.38 to 8.52)	-3.14% (-4.02% to -2.26%)
Switzerland	337.3 (268.73 to 437.55)	119.08 (89.61 to 155.76)	-3.35% (-3.55% to -3.15%)	46.32 (41.71 to 51.85)	16.36 (13.21 to 19.74)	-3.35% (-3.55% to -3.15%)
Syrian Arab Republic	1475.29 (1125.76 to 1859.73)	259.96 (208.27 to 331.63)	-5.17% (-5.98% to -4.36%)	202.93 (128.92 to 307.32)	27.41 (16.91 to 39.92)	-5.54% (-6.46% to -4.6%)
Taiwan (Province of China)	184.54 (147.21 to 228.69)	71.12 (55.15 to 91.58)	-3.58% (-3.82% to -3.35%)	13.55 (11.76 to 15.54)	7.08 (5.69 to 8.42)	-3.06% (-3.85% to -2.27%)
Tajikistan	2497.48 (2027.09 to 3047.88)	992.75 (793.61 to 1248.11)	-3.09% (-3.38% to -2.8%)	343.42 (253.26 to 444.81)	114.73 (61.8 to 178.11)	-3.56% (-3.97% to -3.16%)
Thailand	637.36 (486.04 to 818.42)	171.58 (132.03 to 218.6)	-4.82% (-5.06% to -4.57%)	85.02 (44.23 to 151.36)	8.51 (5.04 to 12.73)	-7.5% (-7.95% to -7.04%)
Timor-Leste	768.16 (595.88 to 982.48)	435.29 (359.38 to 522.31)	-2% (-2.07% to -1.94%)	88.16 (45.78 to 158.98)	34.5 (19.85 to 59.19)	-2.92% (-3.12% to -2.72%)
Togo	5773.88 (4452.86 to 7434)	1885.16 (1509.8 to 2422.73)	-3.98% (-4.57% to -3.39%)	765.07 (506.66 to 1165.13)	195.54 (114.17 to 342.02)	-4.55% (-5.24% to -3.85%)
Tokelau	539.73 (403.24 to 714.68)	1084.33 (814.75 to 1431.21)	-1.85% (-3.35% to -0.32%)	48.33 (27.48 to 86.49)	148.36 (76.97 to 251.85)	-1.44% (-3.28% to 0.43%)
Tonga	2047.71 (1563.09 to 2618.74)	951.89 (766.58 to 1190.45)	-2.5% (-2.72% to -2.28%)	280.62 (187.86 to 417.75)	109.85 (66.4 to 177.94)	-3% (-3.25% to -2.76%)
Trinidad and Tobago	1532.67 (1256.1 to 1905.12)	303.93 (237.73 to 385.78)	-5.64% (-5.91% to -5.36%)	210.48 (175.28 to 250.25)	41.81 (27.05 to 67.6)	-5.63% (-5.9% to -5.36%)
Tunisia	752.67 (573.12 to 958.54)	134.47 (106.33 to 172.31)	-5.49% (-5.78% to -5.2%)	103.39 (55.75 to 177.61)	14.23 (7.16 to 24.64)	-5.72% (-6.09% to -5.36%)

Location	Incidence rate per 100,000 population			Death rate per 100,000 population		
	1990	2021	EAPC, 1990-2021	1990	2021	EAPC, 1990-2021
Turkey	892.63 (681.9 to 1155.86)	70.9 (57.37 to 88.72)	-8.19% (-8.57% to -7.8%)	122.37 (76.09 to 197.56)	9.72 (6.07 to 13.82)	-8.18% (-8.56% to -7.8%)
Turkmenistan	1645.39 (1357.38 to 2002.19)	344.82 (287.44 to 416.42)	-5.58% (-6.14% to -5.02%)	206.69 (172.26 to 247.31)	22.19 (16.36 to 31.53)	-7.12% (-7.98% to -6.24%)
Tuvalu	1026.15 (779.91 to 1321.94)	276.49 (210.84 to 348.32)	-4.59% (-4.75% to -4.43%)	126.57 (72.89 to 199.66)	28.7 (15.13 to 52.42)	-4.91% (-5.13% to -4.69%)
Uganda	7056.04 (5556.05 to 8890.11)	2716.46 (2237.16 to 3352.48)	-3.21% (-3.47% to -2.94%)	963.21 (621.97 to 1570.16)	285.29 (168.87 to 458.21)	-3.85% (-4.19% to -3.5%)
Ukraine	1289.57 (941.19 to 1757.56)	241.84 (188.56 to 312.28)	-6.1% (-6.4% to -5.8%)	176.81 (151.97 to 205.81)	21.78 (17.71 to 26.83)	-7.13% (-7.43% to -6.83%)
United Arab Emirates	712.66 (536.77 to 944.82)	74.92 (57.14 to 100.69)	-6.57% (-6.89% to -6.24%)	97.79 (61.8 to 142.96)	8.58 (4.89 to 13.47)	-6.77% (-7.16% to -6.38%)
United Kingdom	232.97 (174.18 to 314.26)	85.54 (66.97 to 110.09)	-3.84% (-4.17% to -3.52%)	30.53 (28.97 to 32.5)	10.59 (8.8 to 12.12)	-3.93% (-4.32% to -3.53%)
United Republic of Tanzania	5397.76 (4174.17 to 6853.24)	1959.35 (1590.2 to 2433.01)	-3.24% (-3.67% to -2.8%)	742.53 (468.17 to 1210.43)	187.7 (102.84 to 340.1)	-4.25% (-4.78% to -3.72%)
United States of America	143.55 (102.79 to 198.2)	41.6 (32.37 to 53.28)	-3.56% (-3.9% to -3.21%)	19.22 (18.24 to 20.72)	5.64 (4.98 to 6.34)	-3.44% (-3.77% to -3.12%)
United States Virgin Islands	449.59 (334.85 to 593.52)	215.75 (163.43 to 290.29)	-2.49% (-2.66% to -2.32%)	43.3 (26.83 to 70.19)	7.47 (4.42 to 11.55)	-5.36% (-5.85% to -4.87%)
Uruguay	637.01 (516.79 to 764.17)	113.4 (92 to 141.83)	-5.65% (-5.92% to -5.38%)	87.49 (79.6 to 95.2)	15.58 (12.03 to 19.56)	-5.65% (-5.92% to -5.38%)
Uzbekistan	1406.63 (1171.18 to 1713.93)	156.75 (123.57 to 200.13)	-7.92% (-8.58% to -7.26%)	180.2 (156.66 to 210.35)	15.54 (12.2 to 19.45)	-8.56% (-9.4% to -7.7%)
Vanuatu	672.34 (514.55 to 877.61)	383.28 (302.24 to 483.57)	-1.98% (-2.16% to -1.79%)	69.01 (38.07 to 122.8)	32.75 (16.5 to 63.82)	-2.68% (-3.13% to -2.22%)
Venezuela (Bolivarian Republic of)	1135.84 (930.34 to 1387.73)	416.93 (340.17 to 494.41)	-3.09% (-4.18% to -1.98%)	156.02 (136.35 to 178.46)	57.27 (41.65 to 76.04)	-3.06% (-4.2% to -1.92%)
Viet Nam	915.29 (691.5 to 1180.84)	263.01 (207.53 to 326.13)	-4.36% (-4.52% to -4.2%)	125.63 (78.3 to 187.03)	28.26 (15.39 to 45.6)	-5.02% (-5.19% to -4.84%)
Yemen	1219.4 (929.13 to	313.42 (247.25 to	-4.86% (-5.18% to -	167.15 (86.39 to	36.53 (15.38 to	-5.29% (-5.67% to

Location	Incidence rate per 100,000 population			Death rate per 100,000 population		
	1990	2021	EAPC, 1990-2021	1990	2021	EAPC, 1990-2021
	1560.73)	398.11)	4.54%)	294.55)	74.83)	-4.9%)
Zambia	9261.13 (7119.69 to 11725.9)	2205.49 (1766.86 to 2761.66)	-4.3% (-4.74% to - 3.85%)	1271.09 (943.51 to 1779.17)	238.98 (135.69 to 398.63)	-4.8% (-5.38% to -4.22%)
Zimbabwe	2057.04 (1630.29 to 2626.39)	1242.52 (1017.14 to 1477.73)	-1.51% (-2.03% to - 0.99%)	282.49 (195.54 to 413.79)	146.09 (84.45 to 273.44)	-1.94% (-2.58% to -1.3%)

S14 Table. Deaths of meningitis among neonates (0-27 days) in 1990 and 2021, with EAPC from 1990 to 2021.

	Deaths in 1990	Rate in 1990 (per 100,000)	Deaths in 2021	Rate in 2021 (per 100,000)	Changes of deaths, 1990-2021	Changes of death rates, 1990-2021	EAPC 1990-2021
Global	30464 (26319 to 39236)	303.44 (262.15 to 390.81)	12639 (9819 to 16643)	129.69 (100.76 to 170.78)	-58.51% (-67.16 to -44.71)	-57.26% (-66.17 to -43.04)	-2.8% (-3.16 to -2.44)
**Age**							
0-6 days	12134 (10186 to 16870)	477.9 (401.17 to 664.44)	6849 (5261 to 9318)	279.42 (214.61 to 380.14)	-43.55% (-56.81 to -21.89)	-41.53% (-55.26 to -19.09)	-1.93% (-2.37 to -1.49)
7-27 days	18330 (15834 to 22378)	244.38 (211.11 to 298.36)	5790 (4475 to 7771)	79.38 (61.36 to 106.54)	-68.41% (-74.82 to -58.48)	-67.52% (-74.11 to -57.31)	-3.56% (-3.87 to -3.24)
**Sex**							
Male	17958 (14582 to 22908)	345.7 (280.71 to 440.98)	7428 (5333 to 10812)	147.49 (105.89 to 214.67)	-58.64% (-68.96 to -36.64)	-57.34% (-67.98 to -34.65)	-2.92% (-3.27 to -2.56)
Female	12506 (10536 to 16025)	258.12 (217.46 to 330.77)	5211 (4151 to 7052)	110.66 (88.16 to 149.76)	-58.33% (-66.47 to -49.35)	-57.13% (-65.5 to -47.89)	-2.63% (-3.01 to -2.25)
**5 SDI quintiles regions**							
High SDI	272 (259 to 290)	28.66 (27.23 to 30.5)	50 (44 to 56)	6.38 (5.64 to 7.13)	-81.63% (-83.68 to -79.61)	-77.73% (-80.21 to -75.27)	-4.72% (-5 to -4.44)
High-middle SDI	1173 (1037 to 1428)	84.67 (74.83 to 103.06)	118 (97 to 141)	13.28 (10.89 to 15.82)	-89.9% (-91.86 to -87.68)	-84.31% (-87.35 to -80.86)	-6.12% (-6.3 to -5.94)
Middle SDI	4305 (3736 to 5471)	136.74 (118.66 to 173.75)	931 (730 to 1207)	38.5 (30.16 to 49.88)	-78.37% (-83.23 to -70.94)	-71.84% (-78.18 to -62.18)	-4.02% (-4.35 to -3.69)
Low-middle SDI	9777 (8480 to 12412)	337.69 (292.91 to 428.74)	3203 (2444 to 4348)	109.2 (83.31 to 148.22)	-67.23% (-75.14 to -54.02)	-67.66% (-75.46 to -54.63)	-3.36% (-3.74 to -2.98)
Low SDI	14912 (12424 to 19461)	902.69 (752.09 to 1178.07)	8326 (6434 to 11337)	307.32 (237.51 to 418.48)	-44.17% (-56.14 to -24.23)	-65.95% (-73.26 to -53.8)	-3.38% (-3.69 to -3.06)
**GBD regions**							
Andean Latin America	129 (103 to 171)	145.99 (116.03 to 193.66)	17 (11 to 25)	17.61 (11.84 to 26.62)	-87.18% (-91.54 to -79.9)	-87.94% (-92.04 to -81.1)	-6.56% (-6.79 to -6.32)
Australasia	8 (7 to 9)	32.58 (29.79 to 35.67)	2 (2 to 3)	9.13 (7.58 to 10.89)	-68.37% (-74.23 to -61.23)	-71.97% (-77.17 to -65.66)	-4.73% (-5.29 to -4.18)
Caribbean	388 (267 to 578)	571.37 (393.91 to 852.57)	163 (102 to 275)	270.98 (170.59 to 457.9)	-58.03% (-75.99 to -27.32)	-52.57% (-72.87 to -17.87)	-1.94% (-2.18 to -1.71)

	Deaths in 1990	Rate in 1990 (per 100,000)	Deaths in 2021	Rate in 2021 (per 100,000)	Changes of deaths, 1990-2021	Changes of death rates, 1990-2021	EAPC 1990-2021
Central Asia	295 (261 to 329)	198.97 (175.56 to 221.93)	47 (33 to 64)	30.02 (21.5 to 41.09)	-84.24% (-89.03 to -78.27)	-84.91% (-89.5 to -79.19)	-6.69% (-7.28 to -6.1)
Central Europe	116 (110 to 123)	89.09 (84.32 to 94.49)	8 (7 to 10)	10.34 (8.57 to 12.63)	-92.91% (-94.19 to -91.32)	-88.4% (-90.5 to -85.8)	-6.64% (-6.91 to -6.38)
Central Latin America	391 (366 to 419)	103.61 (97.2 to 111.21)	62 (45 to 84)	20.97 (15.1 to 28.21)	-84.09% (-88.6 to -78.78)	-79.76% (-85.49 to -73)	-5.02% (-5.5 to -4.53)
Central Sub-Saharan Africa	1859 (1231 to 2693)	967.16 (640.3 to 1400.83)	757 (520 to 1104)	225.44 (154.71 to 328.64)	-59.28% (-74.8 to -37.19)	-76.69% (-85.58 to -64.05)	-4.94% (-5.46 to -4.42)
East Asia	1200 (811 to 1638)	66.36 (44.82 to 90.56)	69 (50 to 87)	7.84 (5.66 to 9.99)	-94.28% (-96.11 to -92.08)	-88.18% (-91.98 to -83.66)	-6.91% (-7.34 to -6.48)
Eastern Europe	350 (332 to 368)	153.96 (146.44 to 162.08)	28 (25 to 32)	20.34 (17.98 to 23.06)	-92.05% (-93.04 to -90.86)	-86.79% (-88.44 to -84.82)	-6.81% (-7.09 to -6.53)
Eastern Sub-Saharan Africa	6987 (5510 to 9604)	1042.56 (822.21 to 1433.12)	3120 (2341 to 4501)	302.88 (227.21 to 436.89)	-55.34% (-66.58 to -36.79)	-70.95% (-78.26 to -58.88)	-3.99% (-4.29 to -3.69)
High-income Asia Pacific	35 (30 to 42)	23.58 (19.68 to 27.67)	3 (2 to 3)	3.01 (2.59 to 3.46)	-92.3% (-93.72 to -90.67)	-87.22% (-89.59 to -84.52)	-6.16% (-6.49 to -5.83)
High-income North America	65 (62 to 70)	18.69 (17.74 to 20.03)	17 (15 to 19)	5.51 (4.9 to 6.18)	-73.82% (-77.09 to -70.57)	-70.54% (-74.22 to -66.88)	-3.43% (-3.73 to -3.13)
North Africa and Middle East	1736 (1407 to 2406)	210.88 (170.97 to 292.38)	443 (295 to 674)	48.99 (32.62 to 74.58)	-74.49% (-82.89 to -60.86)	-76.77% (-84.42 to -64.36)	-5.09% (-5.59 to -4.58)
Oceania	17 (12 to 25)	101.68 (69.84 to 150.05)	15 (7 to 27)	45.08 (22.64 to 83.36)	-15.5% (-56.38 to 34.7)	-55.67% (-77.11 to -29.33)	-5.47% (-6.66 to -4.27)
South Asia	6238 (5277 to 7437)	243.34 (205.87 to 290.12)	2021 (1432 to 2955)	84.98 (60.21 to 124.25)	-67.6% (-76.73 to -51.49)	-65.08% (-74.92 to -47.72)	-2.71% (-3.1 to -2.32)
Southeast Asia	1663 (1334 to 2275)	178.84 (143.42 to 244.61)	476 (345 to 639)	55.94 (40.57 to 75.02)	-71.38% (-79.76 to -59.85)	-68.72% (-77.88 to -56.13)	-3.67% (-3.88 to -3.47)
Southern Latin America	75 (71 to 80)	94.29 (88.47 to 100.38)	11 (8 to 13)	17.84 (13.91 to 22.44)	-86.06% (-89.13 to -82.44)	-81.08% (-85.25 to -76.17)	-5.41% (-5.61 to -5.21)
Southern Sub-Saharan Africa	265 (208 to 336)	217.35 (171.02 to 275.45)	112 (79 to 165)	91.29 (64.09 to 133.81)	-57.62% (-70.7 to -32.98)	-58% (-70.96 to -33.58)	-2.89% (-3.19 to -2.58)
Tropical Latin America	303 (274 to 332)	119.42 (108.02 to 131.04)	21 (17 to 26)	8.01 (6.32 to 9.88)	-93.08% (-94.6 to -91.37)	-93.29% (-94.76 to -91.63)	-8.81% (-9.12 to -8.51)

	Deaths in 1990	Rate in 1990 (per 100,000)	Deaths in 2021	Rate in 2021 (per 100,000)	Changes of deaths, 1990-2021	Changes of death rates, 1990-2021	EAPC 1990-2021
Western Europe	102 (98 to 107)	29.05 (27.97 to 30.34)	26 (23 to 30)	8.4 (7.23 to 9.65)	-74.36% (-77.87 to -70.32)	-71.1% (-75.06 to -66.54)	-4.05% (-4.29 to -3.82)
Western Sub-Saharan Africa	8242 (6939 to 11070)	1235.42 (1040.15 to 1659.42)	5222 (3960 to 7081)	391.58 (296.95 to 530.94)	-36.64% (-51.44 to -15.54)	-68.3% (-75.71 to -57.75)	-3.61% (-4 to -3.22)

S15 Table. Death of childhood meningitis attributable to risk factors, in 1990 and 2021, with EAPC from 1990 to 2021

Risk factors	Number in 1990	Death Rate in 1990	Number in 2021	Death rate in 2021	EAPC, 1990-2021
**All risk factors**	23689 (20321 to 30187)	235.96 (202.41 to 300.68)	10098 (7798 to 13329)	103.62 (80.02 to 136.77)	-2.73% (-3.11 to -2.35)
**Environmental/occupational risks**	7954 (6669 to 10128)	79.23 (66.42 to 100.88)	3434 (2619 to 4551)	35.24 (26.88 to 46.7)	-2.7% (-3.1 to -2.31)
**Air pollution**	7954 (6669 to 10128)	79.23 (66.42 to 100.88)	3434 (2619 to 4551)	35.24 (26.88 to 46.7)	-2.7% (-3.1 to -2.31)
**Particulate matter pollution**	7954 (6669 to 10128)	79.23 (66.42 to 100.88)	3434 (2619 to 4551)	35.24 (26.88 to 46.7)	-2.7% (-3.1 to -2.31)
Ambient particulate matter pollution	1283 (837 to 1861)	12.78 (8.33 to 18.53)	738 (429 to 1214)	7.57 (4.4 to 12.45)	-1.13% (-1.45 to -0.8)
Household air pollution from solid fuels	6670 (5464 to 8607)	66.44 (54.42 to 85.73)	2696 (2047 to 3740)	27.66 (21.01 to 38.38)	-3.07% (-3.52 to -2.62)
**Behavioral risks**	21554 (18545 to 27524)	214.69 (184.72 to 274.16)	9222 (7113 to 12210)	94.63 (72.99 to 125.29)	-2.72% (-3.1 to -2.34)
**Child and maternal malnutrition**	21554 (18545 to 27524)	214.69 (184.72 to 274.16)	9222 (7113 to 12210)	94.63 (72.99 to 125.29)	-2.72% (-3.1 to -2.34)
**Low birth weight and short gestation**	21554 (18545 to 27524)	214.69 (184.72 to 274.16)	9222 (7113 to 12210)	94.63 (72.99 to 125.29)	-2.72% (-3.1 to -2.34)
**Short gestation**	8529 (7137 to 10829)	84.95 (71.09 to 107.86)	3819 (2886 to 5235)	39.19 (29.61 to 53.72)	-2.6% (-3.02 to -2.17)
Low birth weight	19906 (17027 to 25371)	198.28 (169.6 to 252.71)	8455 (6559 to 11192)	86.76 (67.31 to 114.85)	-2.74% (-3.12 to -2.36)
